# Supplementary material for: Direct-to-consumer tests advertised online in Australia and their implications for medical overuse: systematic online review and a typology of clinical utility
Source: BMJ Open. 2023 Dec 27;13(12):e074205. doi: 10.1136/bmjopen-2023-074205 (PMC10759116; doi:10.1136/bmjopen-2023-074205)
Supplement: Supplementary data [file bmjopen-2023-074205supp001.pdf]

Supplement 1

The following table presents the evidence used to support the inclusion of the tests into respective Categories and Subgroups, and the search and inclusion strategy used.

1. Category 1: Tests with potential clinical utility

Evidence to support inclusion

The test is recommended for asymptomatic screening of specific population groups by a recognised professional medical organisation, determined by a reported evidence-based medicine (EBM) approach.

Search & inclusion strategy

1. For each test, a Google Advanced search using key words: (“*medical condition or test purpose*, *analyte and/or testing method – see column 1”*) AND Screen* OR test* AND Recommend* OR Guide* AND Australia.
2. The first 30 records were screened for relevance. We prefaced Australian and Australasian professional organisations first, and then international professional organisations. The country or region of origin of the recommendations/guidelines is reported in Column 2.

**Medical condition or test purpose; analyte and/or testing method**	**Reason for inclusion & Type of supporting evidence**	**Recommendation or summary of evidence**	**Reference**
Bowel cancer (faecal occult blood test)	Recommended as an asymptomatic screening test for certain population groups; Government organisation (Australia)	Bowel and colorectal cancer screening is recommended for Australians aged 50 - 74 by the National Bowel Cancer Screening Program (every two years).	Australian Government, Department of Health and Aged Care (2022) National Bowel Cancer Screening Program, https://www.health.gov.au/initiatives-and-programs/national-bowel-cancer-screening-program. Accessed 19 June 2022.
Cardiovascular disease risk factors (Lipoproteins)	Recommended as an asymptomatic screening test for certain population groups; Recognised professional group (Australasia)	Adults should have their blood lipids assessed every five years starting at 45 years of age. Lipid levels should be interpreted in the context of an absolute CVD risk assessment after 45 years of age (35 years of age for Aboriginal and Torres Strait Islander peoples). Aboriginal and Torres Strait Islander adults should have lipid tests performed every five years from 35 years of age.	Royal Australasian College of General Practitioners (RACGP) (2021) Guidelines for preventive activities in general practice https://www.racgp.org.au/getattachment/1ad1a26f-9c8b-4e3c-b45b-3237272b3a04/Guidelines-for-preventive-activities-in-general-practice.aspx. Accessed 12 June 2022.
Chlamydia (Chlamydia trachomatis antigen)	Recommended as an asymptomatic screening test for certain population groups; Recognised professional groups (Australasia)	Chlamydia testing should be included in a standard STI check-up (gonorrhoea, chlamydia, HIV, syphilis) for asymptomatic sexually active populations. Routine screening is also recommended for men who have sex with men, young (heterosexual) Aboriginal and Torres Strait Islander people living in remote and very	RACGP (2021) Guidelines for preventive activities in general practice https://www.racgp.org.au/clinical-resources/clinical-guidelines/key-racgp-guidelines/view-all-racgp-guidelines/guidelines-for-

Shih P, *et al. BMJ Open* 2023; 13:e074205. doi: 10.1136/bmjopen-2023-074205

		remote areas, and travellers returning from high prevalence areas overseas.	preventive-activities-in-general-pr/communicable-diseases/sexually-transmissible-infections. Accessed 12 June 2022.<br>Australasian Society for HIV, Viral Hepatitis and Sexual Health Medicine (ASHM), Australian STI Management Guidelines (2022)<br>https://sti.guidelines.org.au/standard-asymptomatic-checkup/<br>. Accessed 12 June 2022.
Cholesterol level (Blood Cholesterol)	Recommended as an asymptomatic screening test for certain population groups; Recognised professional group (Australasia)	Adults 45 and over have their blood cholesterol level assessed every five years. Lipid levels should be interpreted in the context of an absolute CVD risk assessment after 45 years of age (35 years of age for Aboriginal and Torres Strait Islander peoples; Aboriginal and Torres Strait Islander adults should have lipid tests performed every five years from 35 years of age. For those with established CHD, or those at high risk, screening should be yearly.	RACGP (2022) Guidelines for prevention activities in general practice, Cholesterol and other lipids,<br>https://www.racgp.org.au/clinical-resources/clinical-guidelines/key-racgp-guidelines/view-all-racgp-guidelines/guidelines-for-preventive-activities-in-general-pr/prevention-of-vascular-and-metabolic-disease/cholesterol-and-other-lipids. Accessed 31 May 2022.
Diabetes (HbA1c, glucose)	Recommended as an asymptomatic screening test for certain population groups; Recognised professional group (Australasia)	Fasting glucose or glycated Hb is recommended every 3 years for people at high risk of Type 2 Diabetes (40+ and being overweight or obese, AUSDRISK score (questionnaire based risk calculator) of 12 or more. Consider screening for populations with increased risk, e.g. first-degree relative with diabetes, high-risk ethnicity, history of a previous cardiovascular event, women with history of gestational diabetes mellitus and polycystic ovary syndrome, patients on antipsychotic drugs); Those with impaired glucose tolerance test or impaired fasting glucose (not limited by age) should have fasting blood glucose tested every 12 months.	RACGP (2021) Guidelines for preventive activities in general practice<br>https://www.racgp.org.au/getattachment/1ad1a26f-9c8b-4e3c-b45b-3237272b3a04/Guidelines-for-preventive-activities-in-general-practice.aspx. Accessed 12 June 2022.
Gonorrhoea (Neisseria gonorrhoea antigen)	Recommended as an asymptomatic screening test for certain population groups; Recognised professional group (Australasia)	Gonorrhoea should be included in a standard STI check-up (gonorrhoea, chlamydia, HIV, syphilis) for asymptomatic sexually active populations. Routine screening is also recommended for men who have sex with men, young (heterosexual) Aboriginal and Torres Strait Islander people living in remote and very remote areas, and travellers returning from high prevalence areas overseas.	RACGP (2021) Guidelines for preventive activities in general practice<br>https://www.racgp.org.au/clinical-resources/clinical-guidelines/key-racgp-guidelines/view-all-racgp-guidelines/guidelines-for-preventive-activities-in-general-pr/communicable-diseases/sexually-transmissible-infections. Accessed 12 June

			2022. Australasian Society for HIV, Viral Hepatitis and Sexual Health Medicine (ASHM), Australian STI Management Guidelines (2022) https://sti.guidelines.org.au/standard-asymptomatic-checkup/. Accessed 12 June 2022.
Hepatitis B (Hepatitis B surface antigen)	Recommended as an asymptomatic screening test for certain population groups; Recognised professional group (Australasia)	Most people with chronic hepatitis B are asymptomatic unless their disease progresses (latent Hepatitis B). Opportunistic screening is recommended 1for high-risk groups (pp. 20) including people born in countries with a high prevalence, and Aboriginal and Torres Strait Island peoples.	ASHM, Testing Policy - Hepatitis B (2022) https://testingportal.ashm.org.au/files/ASHM_TestingPolicy_2020_HepatitisB_07_2.pdf. Accessed 12 June 2022.
Hepatitis C (Hepatitis C virus antibody)	Recommended as an asymptomatic screening test for certain population groups; Recognised professional group (Australasia)	Hepatitis C virus (HCV) testing should be performed as part of STI testing in people living with HIV, current HIV pre-exposure prophylaxis (PrEP) use, history of injecting drug use, anal sex with a partner with HCV infection, incarceration, non-professional tattoos or body piercings or receipt of organs or blood products before 1990. Asymptomatic screening is *not* recommended for other populations.	ASHM, Australian STI Management Guidelines (2022) https://sti.guidelines.org.au/standard-asymptomatic-checkup/. Accessed 12 June 2022.
HIV (HIV-1 & HIV-2 antibody)	Recommended as an asymptomatic screening test for certain population groups; Recognised professional group (Australasia)	HIV should be included in a standard STI check-up (gonorrhoea, chlamydia, HIV, syphilis) for asymptomatic sexually active populations. Routine screening is also recommended for men who have sex with men, young (heterosexual) Aboriginal and Torres Strait Islander people living in remote and very remote areas, and travellers returning from high prevalence areas overseas.	RACGP (2021) Guidelines for preventive activities in general practice https://www.racgp.org.au/clinical-resources/clinical-guidelines/key-racgp-guidelines/view-all-racgp-guidelines/guidelines-for-preventive-activities-in-general-pr/communicable-diseases/sexually-transmissible-infections. Accessed 12 June 2022. Australasian Society for HIV, Viral Hepatitis and Sexual Health Medicine (ASHM), Australian STI Management Guidelines (2022) https://sti.guidelines.org.au/standard-asymptomatic-checkup/. Accessed 12 June 2022.
Syphilis (Treponemal antibody)	Recommended as an asymptomatic screening test for certain population groups; Recognised professional group (Australasia)	Syphilis screening should be included in a standard STI check-up (gonorrhoea, chlamydia, HIV, syphilis) for asymptomatic sexually active populations. Routine screening is also recommended for men who have sex with men, young	RACGP (2021) Guidelines for preventive activities in general practice https://www.racgp.org.au/clinical-resources/clinical-guidelines/key-racgp-

		(heterosexual) Aboriginal and Torres Strait Islander people living in remote and very remote areas, and travellers returning from high prevalence areas overseas.	guidelines/view-all-racgp-guidelines/guidelines-for-preventive-activities-in-general-pr/communicable-diseases/sexually-transmissible-infections. Accessed 12 June 2022.<br>Australasian Society for HIV, Viral Hepatitis and Sexual Health Medicine (ASHM), Australian STI Management Guidelines (2022)<br>https://sti.guidelines.org.au/standard-asymptomatic-checkup/. Accessed 12 June 2022.
Trichomoniasis infection (Trichomonas vaginalis lipophosphoglycan antibody)	Recommended as an asymptomatic screening test for certain population groups; Recognised professional group (Australasia)	Asymptomatic screening for trichomoniasis is only recommended in certain population groups and situations, e.g. in older people, people from regional and remote areas, especially Aboriginal and Torres Strait Islander people and street-based sex workers. Trichomoniasis is endemic in some Aboriginal and Torres Strait Islander populations in regional/remote areas and people should be tested according to local guidelines.	Australasian Society for HIV, Viral Hepatitis and Sexual Health Medicine (ASHM), Australian STI Management Guidelines (2022)<br>https://sti.guidelines.org.au/sexually-transmissible-infections/trichomoniasis/. Accessed 12 June 2022.

2. Category 2, Subgroup 2A: Tests with limited clinical utility - identified by experts as potentially contributing to medical overuse

Evidence to support inclusion

The test is identified as potentially contributing to medical overuse in certain populations by professionally recognised and EBM recommendations that aim to prevent medical overuse (e.g. Choosing Wisely, NICE 'Do Not Do' Recommendations, other recommendations against testing), as tests clinicians and consumers should question or avoid

Search & inclusion strategy:

1. Search terms for each test: "Do Not Do" OR "Choosing Wisely" AND recommend AND (*analyte and/or testing method - see column 1*) OR (*medical condition or test purpose - see column 1)*
2. Manual search of other known recommendations for and against screening (e.g. RACGP Guidelines for preventative activities in general practice)
3. The first 30 records were screened for relevance. We prefaced Australian and Australasian professional organisations first, and then international professional organisations. The country of origin of the recommendations/guidelines is reported in Column 2.

**Medical condition or test purpose; analyte and/or testing method**	**Reason for inclusion & Type of supporting evidence**	**Recommendation or summary of evidence**	**Reference**
Alzheimer's disease (Apolipoprotein E genotype)	Consumers advised to question the test by a Choosing Wisely Recommendation	Don't undertake genetic testing for methylenetetrahydrofolate reductase (MTHFR), apolipoprotein E (APOE) and other such tests where the clinical utility for	Choosing Wisely Recommendations, Human Genetics Society of Australasia (2022)

	(Australia)	diagnostic purposes is extremely low.	https://www.choosingwisely.org.au/recommendations/hgsa3. Accessed 23 May 2022.
Arthritis (Rheumatoid Factor)	Consumers advised to question the test by a Choosing Wisely Recommendation (United States (US))	Do not order rheumatoid factor (RF) alone, or as part of a 'panel' or 'cascade' in children to evaluate for rheumatologic disease such as juvenile idiopathic arthritis. due to musculoskeletal complaints. Do not let laboratory results guide referral.<br>The rheumatoid factor test is commonly used to help diagnose rheumatoid arthritis. However, a positive RF test does not always mean a person has rheumatoid arthritis, as there are several conditions that give positive rheumatoid factor results. Healthy people without rheumatoid arthritis can also test positive for rheumatoid factor, particularly older people. This does not mean they will develop the condition.	Choosing Wisely Recommendations, American Academy of Paediatrics (2022)<br>https://www.choosingwisely.org/clinician-lists/aap-sorh-rheumatoid-factor-to-evaluate-rheumatologic-disease/. Accessed 18 June 2022.<br>Arthritis Australia (2022)<br>https://arthritisaustralia.com.au/managing-arthritis/medical-management/blood-test-for-arthritis/. Accessed 18 June 2022.
Autoimmune disease (HLA-B27 antibody)	Consumers advised to question the test by a Choosing Wisely Recommendation (Canada)	Don't order an HLA-B27 unless spondyloarthritis is suspected based on specific signs or symptoms. HLA-B27 testing is not useful as a single diagnostic test in a patient with low back pain without further spondyloarthropathy, signs or symptoms because the diagnosis of spondyloarthropathy in these patients is of low probability. There is no clinical utility to ordering an HLA-B27 in the absence of positive imaging or the minimally required SpA signs or symptoms.	Choosing Wisely Recommendations, Canadian Rheumatology Association (2022) https://choosingwiselycanada.org/recommendation/rheumatology/. Accessed 18 June 2022.
Cancer markers (Carcino-embryonic antigen (CEA))	Consumers advised to question the test by a Choosing Wisely Recommendation (Australia)	Do not perform serum tumour marker tests except to evaluate or monitor a cancer known to produce these markers. In patients with non-specific symptoms, testing for a panel of tumour markers to try and diagnose an underlying cancer is not supported by evidence given the low sensitivity and specificity of these tests.	Choosing Wisely Recommendations, Medical Oncology Group of Australia (2022) https://www.choosingwisely.org.au/recommendations/moga4#. Accessed 23 May 2022.
Cancer risk factors (Glutathione S-transferases Genes test)	Consumers advised to question the test by a Choosing Wisely Recommendation (Australia)	Don't undertake genetic testing when clinical diagnostic criteria exist and there are no reproductive or predictive testing implications. Genetic tests do not have inherent utility. It is the adoption of therapeutic or preventive interventions that influences health outcomes.	Choosing Wisely Recommendations, Human Genetics Society of Australasia (2022) https://www.choosingwisely.org.au/recommendations/hgsa5. Accessed 25 August 2022.
Cardiovascular disease risk factors (Homocysteine test)	Consumers advised to question the test by a Choosing Wisely Recommendation (Australia)	Serum homocysteine as a screening test not recommended for low-risk populations. Its value as a risk factor for coronary heart disease is uncertain, and published RCTs show no evidence of benefit by lowering levels.	RCAGP Guidelines for preventative activities in general practice (2022) https://www.racgp.org.au/getattachment/1ad1a26f-9c8b-4e3c-b45b-3237272b3a04/Guidelines-for-preventive-activities-in-general-practice.aspx. Accessed 12 May

			2022.
Cardiovascular disease risk factors (MTHFR & APOE genetic test)	Consumers advised to question the test by a Choosing Wisely Recommendation (Australia)	Don't undertake genetic testing for methylenetetrahydrofolate reductase (MTHFR), apolipoprotein E (APOE) and other such tests where the clinical utility for diagnostic purposes is extremely low.	Choosing Wisely Recommendations, Human Genetics Society of Australasia (2022) https://www.choosingwisely.org.au/recommendations/hgsa3. Accessed 23 May 2022.
Coeliac disease (HLA gene typing)	Consumers advised to question the test by a Choosing Wisely Recommendation (Australia)	Do not undertake genetic testing for coeliac genes as a screening test for coeliac disease." The value of testing for coeliac genes is primarily as a negative test – if the gene test is negative then coeliac disease may be excluded. However as a coeliac gene can be found in approximately one third of the population, a positive result does not make coeliac disease a certainty. Serological testing, in a patient consuming an appropriate amount of gluten, is the appropriate first line screening test for coeliac disease. A small bowel biopsy is then required if serology is positive.	Choosing Wisely Recommendation, Gastroenterological Society of Australia (2022) https://www.choosingwisely.org.au/recommendations/gesa4#. Accessed 23 May 2022.
Food allergy (IgE)	Consumers advised to question the test by a Choosing Wisely Recommendation (Australia)	Food specific IgE testing should not be performed without a clinical history suggestive of IgE-mediated food allergy. While specific Immunoglobulin E (IgE) antibodies can be identified for a wide range of target allergens, the clinical significance of such tests is critically dependent on history. Since specific IgE antibodies are identifiable in many individuals with no evidence of allergic disease, the predictive value of these tests is strongly dependent on a history suggestive of underlying atopic disease with symptoms for which the detected IgE specificity is a plausible trigger.	Choosing Wisely Recommendation, Australasian Society of Clinical Immunology and Allergy (2022) https://www.choosingwisely.org.au/recommendations/ascia4. Accessed 12 May 2022. Australasian Society of Clinical Immunology and Allergy (2022) Laboratory Investigation of Allergic Diseases, https://www.allergy.org.au/hp/papers/tests-in-the-diagnosis-of-allergic-diseases. Accessed 12 May 2022.
Genital Herpes (antibody)	Consumers advised to question the test by a Choosing Wisely Recommendation (Australia)	Do not order herpes serology tests unless there is a clear clinical indication. Herpes serology is not an appropriate screening test in asymptomatic patients and does not accurately confirm whether the person is infected or is a transmission risk to others from asymptomatic shedding. Clinicians also need to consider whether test results will influence treatment or outcomes because, if they do not, then testing is a waste of finite health resources and is not indicated. Herpes serology tests only have good sensitivity and specificity in high prevalence populations. However, selective use of herpes serological tests may be justified for particular groups, such as those at high risk for STIs and human immunodeficiency virus (HIV) infection who are motivated to reduce their sexual risk behaviour; HIV-infected	Choosing Wisely Recommendations, Australasian Chapter of Sexual Health Medicine (2022) https://www.choosingwisely.org.au/recommendations/achshm1. Accessed 25 August 2022

		patients; patients with sexual partners with genital herpes; and in cases where a woman appears to have a first episode of herpes simplex virus (HSV) during pregnancy.	
Haemochromatosis (HFE gene mutation)	Consumers are advised to question the test by a Choosing Wisely Recommendation (US)	Don't order HFE genetic testing for a patient without iron overload or a family history of HFE-associated hereditary hemochromatosis. The majority of hereditary hemochromatosis is due to inheritance of HFE gene mutations. HFE gene mutations are common among individuals of European ancestry; however, only a small proportion of individuals with these mutations develop clinical disease. Other genetic and non-genetic factors contribute to disease expression. HFE genotyping should only be performed among individuals with iron overload or a known family history of HFE-associated hereditary hemochromatosis.	Choosing Wisely Recommendations, American College of Medical Genetics (2022) https://www.choosingwisely.org/clinician-lists/american-college-medical-genetics-genomics-hfe-genetic-testing/. Accessed 1 June 2022.
Heavy metal exposure (Heavy metal in blood)	Consumers are advised to question the test by a Choosing Wisely Recommendation (US)	Don't order heavy metal screening tests to assess non-specific symptoms in the absence of excessive exposure to metals. Individuals are constantly exposed to metals in the environment and often have detectable levels without being poisoned. Indiscriminate testing leads to needless concern when a test returns outside of a 'normal' range. Diagnosis of any metal poisoning requires an appropriate exposure history and clinical findings consistent with poisoning by that metal. A patient should only undergo specific metal testing if there is concern for a specific poisoning based on history and physical examination findings.	Choosing Wisely Recommendations, American College of Medical Toxicology and The American Academy of Clinical Toxicology (2022) https://www.choosingwisely.org/clinician-lists/american-college-academy-medical-toxicology-heavy-metal-screening-tests/. Accessed 10 October 2022
Immune disorders (Immunogenetics)	Consumers advised to question the test by a Choosing Wisely Recommendation (Australia)	Don't undertake genetic testing when clinical diagnostic criteria exist and there are no reproductive or predictive testing implications. Genetic tests do not have inherent utility. It is the adoption of therapeutic or preventive interventions that influences health outcomes.	Choosing Wisely Recommendations, Human Genetics Society of Australasia (2022) https://www.choosingwisely.org.au/recommendations/hgsa5. Accessed 25 August 2022
Insulin resistance (fasting glucose, fasting Insulin)	Consumers advised to question the test by a Choosing Wisely Recommendation (Australia)	Do not measure insulin concentration in the fasting state or during an oral glucose tolerance test to assess insulin sensitivity. Measurement of insulin either in the fasting state or during an oral glucose tolerance test is not a clinically useful method (and may be costly because of the insulin assay) to estimate insulin sensitivity.	Choosing Wisely Recommendations, The Endocrine Society of Australia (2022) https://www.choosingwisely.org.au/recommendations/esa3. Accessed 25 August 2022
Iron deficiency (Iron studies)	The test is not recommended by	Iron studies or serum iron should not be requested to diagnose iron deficiency:	Royal Children's Hospital Melbourne (2022) Clinical

	professional practice guidelines (Australia)	Serum iron reflects recent iron intake and does not provide a measure of the iron stores; Serum ferritin is an acute phase reactant and a normal result does not exclude iron deficiency in the presence of coexisting infection, inflammation or liver disease.	Practice Guidelines – Iron deficiency https://www.rch.org.au/clinicalguide/guideline_index/Iron_deficiency/. Accessed 11 October 2022.
Low-grade Inflammation (high sensitivity C-reactive protein (hsCRP))	Professional guidelines recommend against screening for this condition in certain populations (Australasia)	hsCRP test is a screening test not recommended by the RACGP Guideline for preventative practice, as there is insufficient evidence to support the role of hsCRP in preventive screening of asymptomatic patients.	RACGP (2021) Guidelines for preventive activities in general practice https://www.racgp.org.au/clinical-resources/clinical-guidelines/key-racgp-guidelines/view-all-racgp-guidelines/guidelines-for-preventive-activities-in-general-pr/communicable-diseases/sexually-transmissible-infections. Accessed 12 May 2022.
Neuro genetic disorders (Neurogenetic test)	Consumers advised to question the test by a Choosing Wisely Recommendation (Australia)	Don't undertake genetic testing when clinical diagnostic criteria exist and there are no reproductive or predictive testing implications. Like other screening or diagnostic tests, genetic tests do not have inherent utility. It is the adoption of therapeutic or preventive interventions that influences health outcomes. If clinical diagnostic criteria already exist for the condition in question and there are no reproductive or other predictive testing implications as a result of definitively identifying a genetic cause for the condition, then this renders genetic testing unnecessary.	Choosing Wisely Recommendations, Human Genetics Society of Australasia https://www.choosingwisely.org.au/recommendations/hgsa5. Accessed 25 August 2022
Prostate cancer (Prostate Specific Antigen (PSA))	Professional guidelines recommend against screening for this condition in certain populations (Australasia)	Do not perform PSA testing for prostate cancer screening in men with no symptoms and whose life expectancy is less than 7 years. Screening of asymptomatic (low-risk) men for prostate cancer by PSA testing is not recommended because the benefits have not clearly been shown to outweigh the harms. Pre-test discussion to address the benefits and harms (from overdiagnosis and overtreatment) of prostate cancer screening is recommended.	RACGP Guidelines for Preventative Activities in General Practice (2022) https://www.racgp.org.au/clinical-resources/clinical-guidelines/key-racgp-guidelines/view-all-racgp-guidelines/guidelines-for-preventive-activities-in-general-pr. Accessed 12 May 2022.
Thyroid disease (Thyroid hormones)	Consumers advised to question the test by a Choosing Wisely Recommendation (Australia)	Do not test thyroid function as population screening for asymptomatic patients. Clear evidence on the benefits and harms of screening is unavailable, and recommended against population-based screening. In the absence of evidence that early treatment reduces symptoms, lipid levels, or the risk of cardiovascular disease in patients with mild thyroid dysfunction detected by screening,	Choosing Wisely Recommendations, RACGP https://www.choosingwisely.org.au/recommendations/racgp10. Accessed 21 May 2022. RACGP Guidelines for Preventative Activities in General Practice (2022) https://www.racgp.org.au/clini

		screening for thyroid disease in asymptomatic populations is not recommended. This screening recommendation does not apply to people with symptoms suggestive of thyroid disease.	cal-resources/clinical-guidelines/key-racgp-guidelines/view-all-racgp-guidelines/guidelines-for-preventive-activities-in-general-pr. Accessed 12 May 2022.
Urinary tract infection (urine culture & microscopy)	Consumers advised to question the test by a Choosing Wisely Recommendation (US)	Don't obtain a urine culture unless there are clear signs and symptoms that localise to the urinary tract.	American Family Physician Choosing Wisely Recommendations (2022) https://www.aafp.org/afp/recommendations/viewRecommendation.htm?recommendationId=96. Accessed 21 May 2022.
Vitamin B deficiency (Vitamin B12, B6, B9)	Consumers advised to question the test by a NICE 'Do Not Do' Recommendation (United Kingdom (UK))	Tests for vitamin B12 deficiency & folate (B6) levels should not be carried out unless a full blood count and mean cell volume show a macrocytosis. Vitamin testing should be limited to patients with high-risk for deficiency, e.g. malabsorption or malnutrition	NICE 'Do Not Do Recommendation' (2022) https://www.nice.org.uk/donotdo/tests-for-vitamin-b12-deficiency-should-not-be-carried-out-unless-a-full-blood-count-and-mean-cell-volume-show-a-macrocytosis & https://www.nice.org.uk/donotdo/tests-for-folate-levels-should-not-be-carried-out-unless-a-full-blood-count-and-mean-cell-volume-show-a-macrocytosis. Accessed 31 May 2022.
Vitamin D deficiency (Vitamin D level)	Consumers advised to question the test by a Choosing Wisely Recommendation (Australia)	Do not perform population-based screening for Vitamin D deficiency; Clinicians should be aware that current vitamin D assay methods, may give different results and should understand the limitations of the method used in the laboratory that most of their patients attend.	Choosing Wisely Recommendation, Royal College of Pathologists Australasia (2022) https://www.choosingwisely.org.au/recommendations/rcpa3. Accessed 31 May 2022.
Vitamin K deficiency (Vitamin K level)	Consumers advised to question the test by a Choosing Wisely Recommendation (US)	Vitamin K deficiency is very rare. Measurements of the level of vitamin K in the blood are rarely used to determine if a deficiency exists. A diagnosis of Vitamin K deficiency is typically made by observing the prothrombin time correction following administration of vitamin K, plus the presence of clinical risk factors for vitamin K deficiency.	Choosing Wisely Recommendation, American Family Physician (2022) https://www.aafp.org/afp/recommendations/viewRecommendation.htm?recommendationId=244. Accessed 12 May 2022.

3. Category 2 Subgroup 2B: Tests with limited clinical utility - method has limited accuracy

Evidence to support inclusion

There is credible evidence to suggest the test or testing method has limited accuracy when used on its own

Search & inclusion strategy

1. PubMed and Google Advanced: (*"analyte and/or test method – see column 1")* AND diagnos* AND accuracy OR sensitivity OR specificity.
2. The first 30 records in each database were screened for relevance. i) We first prefaced EBM guidelines issued by Australian, Australasian and international professional or governmental organisations. The country or region of origin of the recommendations/guidelines is reported in Column 2. ii) If none were included, we included the peer-reviewed publication with the highest level of evidence (using Oxford Centre for Evidence-Based Medicine (CEBM): Levels of Evidence Grading[i]) were included. The type of evidence and CEBM grade level used is reported in Column 2. iii) If none were included, we then prefaced web-based consumer information issued by recognised expert health information organisations). A second or third publication may be included if the first publication included is insufficient to support the inclusion criteria.

**Medical condition or test purpose; analyte and/or testing method**	**Reason for inclusion & Type of supporting evidence**	**Recommendation or summary of evidence**	**Reference**
Blood clotting (Prothrombin Time)	Test has limited sensitivity or specificity (Peer-reviewed literature, CEBM Grade Level 1 – Systematic review)	The prothrombin time (PT) test measures the time it takes for blood to form a clot. It is usually used for monitoring patients already under clinical care, e.g. using warfarin and other Vitamin K antagonists to monitor treatment and adjust doses, and for preoperative testing. PT is limited by biological variability, insensitivity to other bleeding disorders, variability in results due to differences in reagents and coagulation. Routine preoperative haemostasis testing with PT is not useful in asymptomatic patients with no known risk factors. High rate of false-positive and false-negative results may lead to inappropriate precautionary measures or false reassurance.	Levy, J. H., Szlam, F., Wolberg, A. S., & Winkler, A. (2014). Clinical use of the activated partial thromboplastin time and prothrombin time for screening: a review of the literature and current guidelines for testing. Clinics in laboratory medicine, 34(3), 453-477.
Chronic fatigue (Iron & Vitamin B12, thyroid hormones)	Test method has limited accuracy when used on its own (Consumer information issued by recognised expert organisation)	Iron and vitamin B12 and thyroid hormones are important biomarkers of chronic fatigue and may play a role in treatment, diagnosis requires comprehensive assessment. Abnormal iron, Vitamin B12 and thyroid hormone levels may be an indicator of a range of other conditions, such as anaemia and thyroid disease.	Health Direct (2022) Chronic fatigue syndrome, https://www.healthdirect.gov.au/chronic-fatigue-syndrome-cfs. Accessed 12 May 2022
Coeliac disease (IgA, IgG antibody)	Test method has limited accuracy when used on its own (Peer-reviewed literature, CEBM Grade Level 5 – Expert opinion based on 'first principles')	Coeliac disease is an immune reaction to eating gluten, and creates inflammation that damages the small intestine's lining. Blood tests that check for antibodies (IgA & IgG) is typically the first step in diagnosing celiac disease, however, serological investigation alone is insufficient to make the diagnosis. A definitive diagnosis of coeliac disease	Lewis, D., Haridy, J., & Newnham, E. D. (2017). Testing for coeliac disease. *Australian prescriber*, *40*(3), 105

		requires gastroscopy and duodenal biopsy. Up to 5% of patients with coeliac disease can have negative serology.	
Gastrointestinal candida (IgG, IgM antibody)	Test method has limited accuracy when used on its own (Peer-reviewed literature, CEBM Grade Level 2 – level 2 evidence synthesis)	The candida fungus is part of the normal gastrointestinal track of healthy populations, and its presence is not sufficient to produce clinically significant candidiasis infection. The standard diagnostic test for gastrointestinal candidiasis is a blood culture. Antibody tests are nonculture diagnostic tests are non-invasive and have the potential to identify patients with currently unrecognised candidiasis and shorten the time to diagnosis. However, no nonculture *Candida* diagnostic tests have been shown to reduce mortality and morbidity, shorten hospital stays, or restrain the emergence of antifungal resistance.	Clancy, C. J., & Nguyen, M. H. (2018). Diagnosing invasive candidiasis. *Journal of Clinical Microbiology*, *56*(5), e01909-17.
Giardia (Rapid antigen test)	Test method has limited accuracy when used on its own (Peer-reviewed literature, CEBM Grade Level 3 – level 3 evidence synthesis)	Several immunodiagnostic tests of rapid diagnosis of giardiasis have been developed in decades, mainly based on the detection of Giardia antigens in faecal specimens. While many of these methods have high sensitivity, they should only serve as a complementary test to the gold standard traditional microscopy stool examination performed in the routine medical laboratory.	Hooshyar, H., Rostamkhani, P., Arbabi, M., & Delavari, M. (2019). Giardia lamblia infection: review of current diagnostic strategies. *Gastroenterology and hepatology from bed to bench*, *12*(1), 3–12.
Helicobacter pylori (Stool antigen)	Test method has limited accuracy when used on its own (Peer-reviewed literature, CEBM Grade Level 1 - Systematic review)	In people with no history of gastrectomy and those who have not recently had antibiotics or proton pump inhibitors, urea breath tests had high diagnostic accuracy while serology and stool antigen tests had lower accuracy to detect H pylori infection (Best et al, 2018).	Best, L. M., Takwoingi, Y., Siddique, S., Selladurai, A., Gandhi, A., Low, B., ... & Gurusamy, K. S. (2018). Non-invasive diagnostic tests for Helicobacter pylori infection. *Cochrane Database of Systematic Reviews*, (3).
Inflammatory bowel disease (Faecal calprotectin test)	The test should only be used based on assessing symptoms and medical history (Consumer information issued by recognised expert organisation and government organisation)	The faecal calprotectin test is used to distinguish between Inflammatory bowel disease (IBD) and irritable bowel syndrome (IBS), which is a functional condition of altered bowel habits with no inflammation. As a screening tool, faecal calprotectin tests reduce the need for invasive diagnostic procedures (such as colonoscopy), potentially leading to earlier diagnosis of IBD and IBS and subsequently improving patient management. However, these tests should only be limited to those necessary, based on symptoms and medical history.	Australian Government, Medical Services Advisory Committee (2022) Medical Measurement of Calprotectin as a marker of bowel inflammation http://www.msac.gov.au/internet/msac/publishing.nsf/Content/1353-public. Accessed 13 October 2022
Kidney disease (Creatinine	Test method has limited accuracy when used on its	Urine dipstick is an inexpensive, non-invasive and accessible test for	White, S. L., Yu, R., Craig, J. C., Polkinghorne, K. R., Atkins, R.

Albumin Ratio)	own (Peer-reviewed literature, CEBM Grade Level 3 – Cross sectional cohort study)	measuring albumin concentration in urine for the purpose of detecting kidney disease. However, this method has high false-positive rates, emphasising the need for laboratory confirmation of positive results. Laboratory Creatinine/Albumin Ratio is a recommended screening test for kidney disease, and is useful in managing diabetes and assessing the risk and progression of diabetic nephropathy. However, the tests should only be limited to those necessary, based on symptoms and medical history.	C., & Chadban, S. J. (2011). Diagnostic accuracy of urine dipsticks for detection of albuminuria in the general community. American Journal of Kidney Diseases, 58(1), 19-28.
Liver function (Bilirubin dipstick)	Test method has limited accuracy when used on its own (Peer-reviewed literature, CEBM Grade Level 4 - Retrospective review)	A urine dipstick measuring urine-bilirubin is a common and non-invasive measure of bilirubin, however, it is known to yield a high rate of false-positive results. In patients with an unexpected positive urine bilirubin test result, 85% had abnormal liver function test results after their positive urine bilirubin result. However, unexpected positive results amounted to only 0.13% of all test results. Urine bilirubin does not appear to add significant information toward the diagnosis of liver disease in most patients.	Foley, K. F., & Wasserman, J. (2014). Are unexpected positive dipstick urine bilirubin results clinically significant? A retrospective review. *Laboratory Medicine*, *45*(1), 59-61.
Measles, mumps, rubella serology (Antibodies)	Test method has limited accuracy when used on its own (Guideline issued by recognised professional organisation)	Routine serological testing for varicella does not provide a reliable measure of vaccine-induced immunity. Although the test can indicate whether natural immunity has occurred due to prior infection, it does not reliably guide diagnosis of clinically significant disease.	RACGP Guidelines for Preventative Activities in General Practice (2022) https://www.racgp.org.au/clinical-resources/clinical-guidelines/key-racgp-guidelines/view-all-racgp-guidelines/guidelines-for-preventive-activities-in-general-pr . Accessed 13 October 2022
Pancreatic insufficiency (pancreatic elastase 1)	Test method has limited accuracy when used on its own (Guideline issued by recognised professional organisation)	Pancreatic insufficiency is the inability of the pancreas to produce and/or transport enough digestive enzymes to break down food in the intestine and allow its absorption. The pancreatic elastase 1: test on its own may lead to false positives or misattribution of diagnosis.	Nikfarjam, M., Wilson, J. S., & Smith, R. C. (2017). Diagnosis and management of pancreatic exocrine insufficiency: Australasian Pancreatic Club Pancreatic Enzyme Replacement Therapy Guidelines Working Group. *Medical Journal of Australia*, *207*(4), 161-165.
Pancreatitis (lipase, amylase)	Test method has limited accuracy when used on its own (Peer-reviewed literature, CEBM Grade Level 3 – level 3 evidence synthesis)	Blood test for amylase and lipase rise within hours of an episode of acute pancreatitis are key components of the diagnostic criteria of acute pancreatitis. However, radiological findings are needed to confirm a diagnosis. Other conditions can also cause slightly	Basnayake C, Ratnam D. (2015) Blood tests for acute pancreatitis. Aust Prescr 2015;38:128-30.https://doi.org/10.18773/austprescr.043

		increased lipase levels. Using the test on its own may lead to false positives or misattribution of diagnosis.	
Urinary tract infection (Nitrite and leukocyte esterase dipstick)	The test method is not reliable on its own as a diagnostic test (Peer-reviewed literature, CEBM Grade Level 5 – Expert opinion based on 'first principles')	Urinary tract infections (UTI) or cystitis is a common bacterial infection. Nitrite and leukocyte esterase dipsticks are useful for ruling out and ruling in cystitis, but a positive urine culture with pyuria confirms the diagnosis.	Colgan R, Williams M. (2011) Diagnosis and treatment of acute uncomplicated cystitis. American Family Physician. 84(7):771-6.
Vaginal infection (pH)	The test has limited sensitivity or specificity (Peer-reviewed literature, CEBM Grade Level 3 – Cross sectional cohort study)	Vaginal bacterial infection is common among women of reproductive age. Vaginal pH determination with a swab is relatively sensitive, but less specific in detecting women with bacterial infection. The pH test may be suitable for home screening and direct patients towards the use of over-the-counter (OTC) antifungal medication, however, complicated vaginal infections should be diagnosed by healthcare providers.	Hemalatha, R., Ramalaxmi, B. A., Swetha, E., Balakrishna, N., & Mastromarino, P. (2013). Evaluation of vaginal pH for detection of bacterial vaginosis. *The Indian journal of medical research*, *138*(3), 354.

4. Category 2, Subgroup 2C: Tests with limited clinical utility – condition tested for has low pre-test probability

Evidence to support inclusion (2C)

There is credible evidence to suggests the condition tested for has low pre-test probability among the healthy general population.

Search & inclusion strategy

1. For each test: PubMed (with MeSH terms) and Google Advanced: (*"medical condition or test purpose" – see column 1)* OR ("*testing method and/or analyte" – see column 1*) AND Australia OR prevalence OR diagnos* OR test* OR screen*
2. The first 30 records in each database were screened for relevance. i) We first prefaced EBM guidelines issued by Australian, Australasian and international professional or governmental organisations. The country or region of origin of the recommendations/guidelines is reported in Column 2. ii) If none were included, we included the peer-reviewed publication with the highest level of evidence (using CEBM Grading) were included. The type of evidence and CEBM grade level used is reported in Column 2. iii) If none were included, we then prefaced web-based consumer information issued by recognised expert health information organisations).

Medical condition or test purpose; analyte and/or testing method	Reason for inclusion & Type of supporting evidence	Recommendation or summary of evidence	Reference
Cytomegalovirus (CMV) (IgC & IgM)	The test is for a condition with low pre-test probability in the general population (Consumer information issued by recognised health information organisation)	By the age of 20 years, around 50% of adults would have been infected with CMV, but it rarely causes symptoms. Most people with CMV infection may have a self-limiting flu-like or glandular fever-like illness. CMV tests are only needed to help diagnose a current,	MedlinePlus (2022) Cytomegalovirus (CMV) Tests, https://medlineplus.gov/lab-tests/cytomegalovirus-cmv-tests/ Accessed 31 May 2022

		reactivated, or past CMV infection in people at risk for health complications.	
Epstein-Barr virus (EBV) (EBV antibody)	The test is for a condition with low pre-test probability in the general population (Consumer information issued by recognised professional organisation & government organisation)	Approximately 95% of the world's adult population have been infected by EBV, but it rarely causes symptoms. An EBV antibody test may be needed in non-typical cases of mononucleosis or if another illness caused by EBV infection is suspected, or used to investigate flu-like symptoms in pregnancy.	Pathology Tests Explained (2022) Epstein Barr Virus Antibodies https://pathologytestsexplained.org.au/learning/test-index/epstein-barr-virus-antibodies. Accessed 21 May 2022<br>WebMD (2022) What to Know About the Epstein-Barr Virus Test https://www.webmd.com/a-to-z-guides/what-to-know-epstein-barr-virus-test. Accessed 21 May 2022
Glucose-6-Phosphate-Dehydrogenase deficiency (G6PD) (G6PD enzyme test)	The test is for a condition with low pre-test probability in the general population (Consumer information issued by recognised professional organisation, international)	G6PD deficiency is one of the most common forms of enzyme deficiency, affect more than 400 million people worldwide. However, the vast majority of people remain clinically asymptomatic throughout their lives. G6PD deficiency does not seem to affect life expectancy, quality of life, or the activity of affected individuals. Testing for G6PD deficiency should be considered when an acute haemolytic reaction triggered by exposure to a known oxidative drug, infection, or ingestion of fava beans; and prior to commencing treatment with certain medications such as primaquine, tafenoquine and rasburicase. It is not a routine test. Screening would only be justified for newborns in families with disease and in populations with higher risk for malaria.	Rare Diseases (2022) https://rarediseases.org/rare-diseases/glucose-6-phosphate-dehydrogenase-deficiency/. Accessed 21 October 2022
Lyme disease (Borrelia antibody)	The test is for a condition with low pre-test probability in the general population (Peer-reviewed literature, CEBM Grade Level 3 – level 3 evidence synthesis)	Lyme disease is caused by bacteria transmitted in a tick bite. The usefulness of the serological tests for Lyme disease depends on the pre-test probability and subsequent predictive values in the setting where the tests are being used. The types of tick that carry the bacteria are not native to Australia therefore it is unlikely to transmit Lyme disease. Treating false positive results, or treating patients with "Lyme disease-like illness" can lead to prolonged antibiotic therapy that is unjustified, and is likely to do much more harm than good.	Collignon, P. J., Lum, G. D., & Robson, J. M. (2016). Does Lyme disease exist in Australia? *Medical Journal of Australia*, *205*(9), 413-417.
Rotavirus (Rapid antigen test)	The test is for a condition with low pre-test probability in the general population (Consumer information	Rotavirus is an infectious disease most commonly among children under 5. The symptoms are diarrhoea, vomiting and stomach pains, and can cause	The Australian Government Department of Health (2022) Rotavirus Laboratory Case Definition (LCD)

	issued by government organisation)	severe gastroenteritis. There is no treatment for Rotavirus, but severe symptoms require hospitalisation. Commercially available rapid antigen detections kits are based on the VP6 antigen of group A rotaviruses, which means this is the only rotavirus serogroup detected. Recent experience in Australia has found a high false-positive rate for antigen detection tests compared with PCR, especially in vaccinated children. Confirmation of positive results should be done where there is a low pre-test probability that the person has rotavirus infection.	https://www1.health.gov.au/internet/main/publishing.nsf/Content/cda-phlncd-rotavirus.htm. Accessed 28/5/2022.

5. Category 3: Commercial 'health checks' with low potential clinical utility

Evidence to support inclusion

1. There is insufficient evidence to support clinical utility of the test as a routine assessment of health for low-risk and asymptomatic general practice populations; OR
2. There is literature to suggest the lack of clinical validity as a testing method for the named medical condition.

Search & inclusion strategy

1. For each test: a Google Advanced search using key words: (*"medical condition or test purpose - see column* 1) OR (*"analyte and/or testing method – see column 1"*) AND test* OR Recommend* OR Guide*. The first 30 records were screened for relevance. If no relevant sources were included, then PubMed (with MeSH terms): *(test or analyte – reported in column 1*) AND utility OR test* OR screen*
2. The first 30 records in each database were screened for relevance. i) We first prefaced EBM guidelines issued by Australian, Australasian and international professional or governmental organisations. The country or region of origin of the recommendations/guidelines is reported in Column 2. ii) If none were included, we included the peer-reviewed publication with the highest level of evidence (using CEBM Grading) were included. The type of evidence and CEBM grade level used is reported in Column 2. iii) If none were included, we then prefaced web-based consumer information issued by recognised expert health information organisations).

Medical condition or test purpose; analyte and/or testing method	Reason for inclusion & Type of supporting evidence	Recommendation or summary of evidence	Reference
Biological age (Glycans)	Insufficient evidence to support clinical utility of the test as an indicator of health status among healthy populations (Peer-reviewed literature, CEBM Grade Level 3 – level 3 evidence synthesis)	There is insufficient evidence to demonstrate clinical utility of measuring 'biological age', which is a hypothetical measure of the biological aging process, as opposed to chronological age as a marker of passage of time. There is still limited understanding about the mechanisms by which biological age predictors work. It remains unclear how best to	Jylhävä, J., Pedersen, N. L., & Hägg, S. (2017). Biological age predictors. EBioMedicine, 21, 29-36.<br>Nwanaji-Enwerem, J. C., & Mair, W. B. (2022). Redefining age-based screening and diagnostic guidelines: an opportunity for biological aging clocks in clinical medicine?. The Lancet Healthy

		incorporate measures of biological age into clinical medicine, and there is a need for trials that show modifying epigenetic age acceleration impacts human morbidity and mortality.	Longevity, 3(6), e376-e377.\
Bone health (serum alkaline phosphatase, calcium, potassium)	Insufficient evidence to support clinical utility of the test as an indicator of health status among healthy populations (Consumer information issued by recognised professional organisation, Australia)	An elevated level of bone markers may be seen in conditions such as osteoporosis or Paget disease. However, bone marker testing is typically only indicated in people who have been diagnosed with or are at risk of bone loss. The tests are not intended to be used to screen the general public.	Pathology Tests Explained (2022) Bone Markers, https://pathologytestsexplained.org.au/learning/test-index/bone-markers. Accessed 12 October 2022
Coenzyme Q10 (CoQ10) profile (Coenzyme Q10 level)	Insufficient evidence to support clinical utility of the test as an indicator of health status among healthy populations (Official information issued by government organisation, US)	CoQ10 has important functions in the body, people with some diseases may have reduced levels. However, low or high levels of CoQ10 do not necessarily indicate a problem in a person without symptoms of deficiency. Clinically significant coenzyme deficiency needs to be further diagnosed and not rely on blood plasma CoQ10 measures alone.	US Department of Health, National Centre for Complementary and Integrative Health (2022) Health Information – Coenzyme Q10 https://www.nccih.nih.gov/health/coenzyme-q10. Accessed 12 October 2022
Cytokine profile (Cytokine level)	Insufficient evidence to support clinical utility of the test as an indicator of health status among healthy populations (Guideline issued by recognised professional organisation, Australia)	Cytokines have been shown to regulate immunologic responses, hematopoietic development, and cell-to-cell communication as well as host responses to infectious agents and inflammatory stimuli. However, the clinical usefulness of measuring cytokine levels in plasma remains unclear. There is no definite diagnostic application for cytokine profile test at present.	RCPA Manual (2022) Cytokine Profile Test https://www.rcpa.edu.au/Manuals/RCPA-Manual/Pathology-Tests/C/Cytokine-profile-test. Accessed 6 October 2022
Fertility status - male (Semen analysis)	Insufficient evidence to support clinical utility of the test as an indicator of health status among healthy populations (Peer-reviewed literature, CEBM Grade Level 5 – Expert opinion based on 'first principles')	Semen analysis is subject to considerable interobserver variability and has been difficult to standardise, particularly with regard to the quantification of concentration and morphology. The results of semen analysis are often nuanced, making self-interpretation of results difficult without the guidance of a reproductive physician. Individual semen analysis parameters, even if abnormal, may be weak indicators of a man's fertility potential.	Stanczyk, F. Z., Mandelbaum, R. S., & Lobo, R. A. (2022). Potential pitfalls of reproductive direct-to-consumer testing. *F&S Reports*, *3*(1), 3-7.
Gastrointestinal function (Microbiome analysis)	Insufficient evidence to support clinical utility of the test as an indicator of health status among healthy populations (Guideline issued by recognised professional organisation,	The British Dieticians Association does not advise commercial gut microbiota testing and lifestyle change programs based on the test results. Research to date indicates there is no standard microbiome composition that can be used as a baseline for health and few	British Dieticians' Association, Commercial Gut Microbiome (2021) https://www.bda.uk.com/resource/commercial-gut-microbiome-testing.html. Accessed 11 July 2022.

	UK)	instances where there is a definitive association between any gut microbe and disease risk.	
Gastrointestinal function (Stool Analysis)	Insufficient evidence to support clinical utility of the test as an indicator of health status among healthy populations (Consumer information issued by recognised professional organisation, Australasia)	Laboratory analysis of stools can be useful , especially in gastroenterology. However, commercial stool analysis marketed as ‘functional’ assessment of gastrointestinal health may vary in protocol and the analysis, and do not have clinical utility for healthy populations.	Friends of Science In Medicine (FSM) Recommendations For Pathology Tests In Australia (2013) https://pathologytestsexplained.org.au/lto_au/media/images/FSM-pathologyrecommendations.pdf. Accessed 11 July 2022.
Health & Wellness profile (Panel consisting of tests for more than one of: full blood count, hormones, organ function, vitamins & minerals)	Insufficient evidence to support clinical utility of the test as an indicator of health status among healthy populations (Peer-reviewed literature, CEBM Grade Level 1 - Systematic review of level 1 studies)	General health checks are screening test for more than one disease or risk factor in more than one organ system in a person who does not feel ill. These aim to detect disease and risk factors for disease with the purpose of reducing morbidity and mortality. However, evidence does not suggest general health checks reduces morbidity or mortality, neither overall nor for cardiovascular or cancer causes, although the number of new diagnoses was increased. Important harmful outcomes, such as the number of follow-up diagnostic procedures or short-term psychological effects, were often not studied or reported and many trials had methodological problems.	Krogsbøll, L. T., Jørgensen, K. J., Larsen, C. G., & Gøtzsche, P. C. (2012). General health checks in adults for reducing morbidity and mortality from disease: Cochrane systematic review and meta-analysis. *BMJ*, *345*.
Hormone profile – female fertility status (Panel consisting of tests for more than one of: Follicle Stimulating Hormone, luteinising hormone, estradiol, progesterone, testosterone)	Insufficient evidence to support clinical utility of the test as an indicator of health status among healthy populations (Guideline issued by recognised professional organisation, Canada)	Tests for female reproductive hormone are used to investigate primary or secondary hypogonadism, infertility, prediction of ovulation, and for monitoring patients undergoing fertility treatment. The tests do not indicate the fertility status of healthy populations not under clinical care.	British Columbia Medical Association (2022) Hormone Testing – Indicators and Appropriate Use, https://www2.gov.bc.ca/gov/content/health/practitioner-professional-resources/bc-guidelines/special-endocrine-testing. Accessed 22 May 2022
Hormone profile – female health (Panel consisting of tests for more than one of: Follicle Stimulating Hormone, luteinising hormone, estradiol, progesterone,	Insufficient evidence to support clinical utility of the test as an indicator of health status among healthy populations (Guideline issued by recognised professional organisation, Canada)	Tests for female reproductive hormone are used to investigate primary or secondary hypogonadism, infertility, prediction of ovulation, and for monitoring patients undergoing fertility treatment. The tests do not indicate the general health status of healthy populations not under clinical care.	British Columbia Medical Association (2022) Hormone Testing – Indicators and Appropriate Use, https://www2.gov.bc.ca/gov/content/health/practitioner-professional-resources/bc-guidelines/special-endocrine-testing. Accessed 22 May 2022

testosterone)			
Hormone profile – general health (Panel consisting of tests for more than one of: adrenal, growth, thyroid & reproductive hormones)	Insufficient evidence to support clinical utility of the test as an indicator of health status among healthy populations (Guideline issued by recognised professional organisation, Canada)	Testosterone and luteinising hormone tests have indications for investigating primary or secondary hypogonadism. Sex hormone binding globulin (SHBG) is used to clarify borderline low total testosterone. Estradiaol, dehydroepiandrosterone (DHEA), androstenedione are not indicated in routine clinical practice. The tests do not indicate the general health status of healthy populations not under clinical care.	British Columbia Medical Association (2022) Hormone Testing – Indicators and Appropriate Use, https://www2.gov.bc.ca/gov/content/health/practitioner-professional-resources/bc-guidelines/special-endocrine-testing. Accessed 22 May 2022
Hormone profile – male health (Panel consisting of tests for more than one of: DHEA, estrone, estradiol, testosterone, SHBG, androstenedione)	Insufficient evidence to support clinical utility of the test as an indicator of health status among healthy populations (Guideline issued by recognised professional organisation, Canada)	Testosterone and luteinising hormone tests have indications for investigating primary or secondary hypogonadism. SHBG – clarification of borderline low total testosterone. Estradiaol, DHEA, androstenedione are not indicated in routine clinical practice. The tests do not indicate the general health status of healthy populations not under clinical care.	British Columbia Medical Association (2022) Hormone Testing – Indicators and Appropriate Use, https://www2.gov.bc.ca/gov/content/health/practitioner-professional-resources/bc-guidelines/special-endocrine-testing. Accessed 22 May 2022
Hormone profile – Menopause (Panel consisting of tests for more than one of: estradiol, estriol, estrone, DHEA)	Insufficient evidence to support clinical utility of the test as an indicator of health status among healthy populations (Consumer information issued by recognised professional organisation, US)	Testing hormone levels is not required to determine whether a woman has the “right amount” of hormones. The optimal hormone levels in postmenopausal women have not been established. How symptoms respond to a particular dose of hormones or non-hormonal menopause medication is the only reliable guide. Because hormone levels vary day to day as well as throughout the day, even a blood and saliva test cannot accurately reflect the body’s hormone levels.	The North American Menopause Society (2022) What is hormone testing https://www.menopause.org/publications/clinical-practice-materials/bioidentical-hormone-therapy/what-is-hormone-testing-. Accessed 12 May 2022.
Hormone profile – ovarian reserve (Anti-Mullerian Hormone (AMH)	Insufficient evidence to support clinical utility of the test as an indicator of health status among healthy populations (Peer-reviewed literature, CEBM Grade Level 3 - cohort study)	AMH are used in the clinical setting for fertility treatments. In the general population and those not undergoing treatment for infertility, AMH does not predict future fertility potential, the likelihood of unassisted pregnancy, the time to pregnancy, or the timing of menopause.	Depmann M., Broer S.L., Eijkemans M.J.C., van Rooij I.A.J., Scheffer G.J., Heimensem J., et al. (2017) Anti-Müllerian hormone does not predict time to pregnancy: results of a prospective cohort study. Gynecol Endocrinol. ;33:644–648.
Hormone profile – sleep quality (Cortisol & Melatonin)	Insufficient evidence to support clinical utility of the test as an indicator of health status among healthy populations (Guideline issued by recognised professional organisation, Canada)	Cortisol (24-hour urine free) is used for targeted screening for Cushing Syndrome caused by endogenous cortisol excess only. Cortisol (serum/plasma) is not useful in the investigation of non-specific fatigue and lethargy unless accompanied by clinical syndromes of Addison’s Disease or Cushing Syndrome.	British Columbia Medical Association (2022) Hormone Testing – Indicators and Appropriate Use, https://www2.gov.bc.ca/gov/content/health/practitioner-professional-resources/bc-guidelines/special-endocrine-

		Measurements of morning and afternoon cortisol (for diurnal variation) is not useful.	testing. Accessed 22 May 2022
Hormone profile – Sports & fitness profile (Growth & reproductive hormones)	Insufficient evidence to support clinical utility of the test as an indicator of health status among healthy populations (Guideline issued by recognised professional organisation, Canada)	Screening for growth hormone related disorders with insulin-like growth factor 1 (IGF1) is only indicated in patients demonstrating symptoms of growth hormone excess (acromegaly) or deficiency. The reference intervals are highly dependent on age. Tests for reproductive hormones are used to investigate primary or secondary hypogonadism and other endocrine disease or monitoring patients undergoing fertility treatment. Estradiaol, DHEA, androstenedione are not indicated in routine clinical practice. The tests do not indicate fitness or sports performance level in healthy populations.	British Columbia Medical Association (2022) Hormone Testing – Indicators and Appropriate Use, https://www2.gov.bc.ca/gov/content/health/practitioner-professional-resources/bc-guidelines/special-endocrine-testing. Accessed 22 May 2022.
Hormone profile – stress (salivary cortisol)	Insufficient evidence to support clinical utility of the test as an indicator of health status among healthy populations (Guideline issued by recognised professional organisation, Canada)	Salivary cortisol is used to investigate the cortisol response to stress in a research setting. In a clinical context, the utility of salivary hormone testing is limited to Cushing Syndrome screening using late night salivary cortisol.	Inder, W. J., Dimeski, G., & Russell, A. (2012). Measurement of salivary cortisol in 2012–laboratory techniques and clinical indications. *Clinical endocrinology*, *77*(5), 645-651.
Nutrigenomic profile (genetic)	Insufficient evidence to support clinical utility of the test as an indicator of health status among healthy populations (Guideline issued by recognised professional organisation, international)	The Consensus Report of the Academy of Nutrition and Dietetics suggest there is insufficient evidence regarding the effectiveness of incorporating nutrigenetic testing into nutrition counselling or care and reporting dietary or clinical outcomes at present. Research on the application of nutritional genomics to practice is in its infancy.	Braakhuis, A., Monnard, C. R., Ellis, A., & Rozga, M. (2021). Consensus report of the Academy of Nutrition and Dietetics: incorporating genetic testing into nutrition care. *Journal of the Academy of Nutrition and Dietetics*, *121*(3), 545-552.
Nutritional status (Amino acids)	Insufficient evidence to support clinical utility of the test as an indicator of health status among healthy populations (Guideline issued by recognised professional organisation, US.)	Assessing amino acid alone is not a reliable indication of nutritional status, because the reference range of amino acid concentrations vary with age. Interpretations of amino acid results are based upon relative amino acid levels, pattern recognition, and correlation of positive and negative findings, rather than on individual amino acids levels alone. Amino acid abnormalities or overall profiles should also be considered in the context of clinical findings and/or additional test results.	Sharer, J. D., De Biase, I., Matern, D., Young, S., Bennett, M. J., & Tolun, A. A. (2018). Laboratory analysis of amino acids, 2018 revision: a technical standard of the American College of Medical Genetics and Genomics (ACMG). *Genetics in Medicine*, *20*(12), 1499-1507.
Nutritional status (Ammonia)	Insufficient evidence to support clinical utility of the test as an indicator of health status among healthy	In healthy populations, there is a normal range of ammonia in the blood but this will vary depending on the age of a person. A test for ammonia is not	Tietze KJ. (2012) Review of Laboratory and Diagnostic Tests. In: Tietze KJ, ed. *Clinical Skills for Pharmacists (Third Edition).* Saint

	populations (Peer-reviewed literature, CEBM level 2 – level 2 evidence synthesis)	used as a routine screening test. It is used to diagnose and/or monitor conditions that cause high ammonia levels, such as liver disease, urea cycle disorders**.**	Louis: Mosby:86-122
Nutritional status (Chromium)	Insufficient evidence to support clinical utility of the test as an indicator of health status among healthy populations (Peer-reviewed literature,	Chromium deficiency has not been reported in healthy populations, and no definitive deficiency symptoms have been established. There is a lack of an accepted biomarker of chromium nutritional status.	Vincent JB, Lukaski HC. Chromium. (2018) Adv Nutr. Jul 1;9(4):505-506. doi: 10.1093/advances/nmx021. PMID: 30032219; PMCID: PMC6054252.
Nutritional status (Copper)	Insufficient evidence to support clinical utility of the test as an indicator of health status among healthy populations (Guideline issued by recognised professional organisation, Australasia)	Plasma copper is not a valid indicator of general health and nutritional status. The clinical purpose of a copper test is to diagnose Wilson's disease, Menkes disease and, occasionally, investigation of copper status in parenteral alimentation, and investigation of suspected copper toxicity.	RCPA Manual (2022) https://www.rcpa.edu.au/Manuals/RCPA-Manual/Pathology-Tests/C/Copper. Accessed 31 May 2022.
Nutritional Status (Essential Fatty Acids)	Insufficient evidence to support clinical utility as valid indicator of general health (Consumer information issued by recognised professional organisation)	Essential fatty acid testing or fatty acid profiling involves analysing specific types of fats in the blood stream. Commonly advertised are from fatty acids of the Omega-3 and Omega-6 groups.-Whilst there is good evidence that modifying diet to include more 'good' fats (i.e. omega-3) is beneficial, there is no evidence that testing fatty acids is able to provide any further information or health benefit.	LabTests Online UK (2022) https://labtestsonline.org.uk/tests/unvalidated-or-misleading-laboratory-tests. Accessed 31 May 2022
Nutritional status (Glutathione)	Insufficient evidence to support clinical utility as valid indicator of general health (Peer-reviewed literature, CEBM level 2 – level 2 evidence synthesis)	Measuring glutathione levels can help to determine if a person is deficient and may benefit from supplements or an increased diet of glutathione-rich foods. However, while optimizing glutathione levels has been proposed as a strategy for health promotion and disease prevention, clear, causal relationships between glutathione status and disease risk or treatment remain to be clarified.	Minich, D. M., & Brown, B. I. (2019) A review of dietary (phyto) nutrients for glutathione support. *Nutrients*, *11*(9), 2073.
Nutritional status (Iodine)	Insufficient evidence to prove clinical utility as valid indicator of general health (Peer-reviewed literature, CEBM level 3 – level 3 evidence synthesis)	There are limitations of assessing iodine status in the general population. Urine iodine is highly variable from day to day in a given patient and has low predictive value for iodine deficiency. Median urine iodine concentrations used in epidemiological studies should not be applied to individuals; if they are then iodine deficiency will be overdiagnosed. The need for multiple samples and a long sampling period severely limits the clinical utility of	Wainwright, P., & Cook, P. (2019). The assessment of iodine status– populations, individuals and limitations. *Annals of Clinical Biochemistry*, *56*(1), 7-14.

		such tests.	
Nutritional status (Magnesium test)	Insufficient evidence to prove clinical utility as valid indicator of general health (Peer-reviewed literature, CEBM level 3 – level 3 evidence synthesis)	There is no standardised laboratory test that accurately describes the status of magnesium. Due to the way in which magnesium is compartmentalised, typical blood and urine analytics may not provide an accurate proxy of magnesium status.	Workinger, Jayme L., Robert P. Doyle, and Jonathan Bortz. (2018) Challenges in the diagnosis of magnesium status. *Nutrients* 10, no. 9: 1202.
Nutritional status (Organic acids)	Insufficient evidence to prove clinical utility as valid indicator of general health (Guidelines issued a recognised professional organisation, Australia.	Nutritional status is not an indicator of an organic acid test. The normal range of organic acid levels in healthy asymptomatic individuals is varied. Further studies with well-defined patient groups exhibiting specific symptoms or diseases are warranted in order to discern between normal and pathological values.	RCPA Manual (2022) Organic acids https://www.rcpa.edu.au/Manuals/RCPA-Manual/Pathology-Tests/O/Organic-acids-urine. Accessed 31 May 2022.
Nutritional status (Selenium)	Insufficient evidence to prove clinical utility as valid indicator of general health (Guideline from a recognised professional organisation, Australia; consumer information from a recognised professional organisation, UK)	A selenium test is used for the detection of selenium deficiency in patients with dietary deficiency, especially patients on a non-supplemented synthetic amino acid diet, and the detection of selenium toxicity. Clinically significant selenium deficiency is manifest primarily as cardiomyopathy. This correlates poorly with plasma selenium levels and rarely occurs in selenium deficient patients on total parenteral nutrition. A nutrient test for selenium is useful but only in rare circumstances because clinically relevant deficiency is unlikely	RACP Manual (2022) Selenium https://www.rcpa.edu.au/Manuals/RCPA-Manual/Pathology-Tests/S/Selenium Accessed 11 October 2022. LabTests Online UK (2022) https://labtestsonline.org.uk/tests/unvalidated-or-misleading-laboratory-tests. Accessed 31 May 2022.
Nutritional status (Zinc)	Insufficient evidence to support clinical utility as valid indicator of general health (Guideline from professional and government organisation, Australia, New Zealand)	In a clinical setting, zinc plasma test is used to indicate zinc toxicity, or to assess people at risk of zinc deficiency (e.g. malnutrition, gastrointestinal disease). However, the zinc blood test is not very reliable for people with mild or low-level deficiency, because zinc is only present in small amounts in the body's cells.	RACP Manual (2022) Zinc https://www.rcpa.edu.au/Manuals/RCPA-Manual/Pathology-Tests/Z/Zinc. Accessed 11 October 2022. Auckland District Health Board (2022) Test Guide https://testguide.adhb.govt.nz/EGuide/. Accessed 11 October 2022.
Secretor status (Blood typing) (Antigens)	Insufficient evidence to prove clinical utility as valid indicator of general health (Peer-reviewed literature, CEBM level 1 - Systematic review)	Secretor status describes whether a person's blood type antigens (ABO blood type) are secreted in other body fluid such as saliva and mucus. Secretor status is determined by a FUT2 gene test. Secretor status test has been used to promote the 'Blood-Type Diet', based on the claim that each ABO blood type processes food differently, and adherence to a diet specific to an individual's ABO blood group could improve health, wellbeing, and energy levels and reduce risk of developing diseases such as cancer	Wang, J., García-Bailo, B., Nielsen, D. E., & El-Sohemy, A. (2014). ABO genotype,'blood-type'diet and cardiometabolic risk factors. *PloS one*, *9*(1), e84749.

		and cardiovascular disease. However, no evidence currently exists to validate the purported health benefits of blood type diets.	

6. Category 4, Subgroup 4A: Non-evidence-based tests with no potential clinical utility (Testing method is not recognised by the general medical community)

Description

The test method or condition tested for is not recognised by the general medical community. Current evidence does not support the validity of the test or the condition tested for. The results of non-evidence-based tests does not provide reliable clinical information to guide effective and beneficial management.

Evidence to support inclusion (4A):

There is insufficient evidence to support the clinical validity of the testing method for the condition it aims to test for.

Search & inclusion strategy

1. Google Advanced search terms: (*"test method and/or analyte" - see column 1)* AND diagnos* OR test* OR evidence*
2. PubMed and Google Advanced: (*"test method and/or analyte"- see column 1)* AND diagnos* OR test* OR evidence*

The first 30 records in each database were screened for relevance. i) We first prefaced EBM guidelines issued by Australian, Australasian and international professional or governmental organisations. The country or region of origin of the recommendations/guidelines is reported in Column 2. ii) If none were included, we included the peer-reviewed publication with the highest level of evidence (using CEBM Grading) were included. The type of evidence and CEBM grade level used is reported in Column 2. iii) If none were included, we then prefaced web-based consumer information issued by recognised expert health information organisations).

**Medical condition or test purpose; analyte and/or testing method**	**Reason for inclusion & Type of supporting evidence**	**Recommendation or summary of evidence**	**Reference**
Environmental toxins (Hair metal & mineral analysis)	Insufficient evidence to support the validity of the test method in the general medical community (Official information from government organisation, US)	A number of scientific issues need to be resolved before hair analysis can become a useful tool to understand environmental exposures. Although hair analysis may answer some questions about environmental exposure to certain substances, hair analysis often raises more questions than they answer. With very few exceptions, hair analysis results provide no insights as to whether an individual will develop health effects.	Agency for Toxic Substances and Disease Registry (2022) Analysis of Hair Samples: How Do Hair Sampling Results Relate to Environmental Exposures? https://www.atsdr.cdc.gov/HAC/hair_analysis/03-0330HairSampleTesting-Scientific.pdf. Accessed 10 October 2022.
Environmental toxins (Mycotoxin test)	Insufficient evidence to support the validity of the test method in the general medical community (Guideline issued by recognised intergovernmental organisation,	Mycotoxins are metabolites of some fungi that can cause illness primarily after ingestion of contaminated foods. Low levels of mycotoxins are found in many foods; therefore, mycotoxins are found in the urine of healthy persons. Mycotoxin levels that predict disease have not been established. Although	World Health Organizataion (2009) WHO guidelines for indoor air quality: Dampness and mould. http://www.who.int/indoorair/publications/7989289041683/en/. Accessed 10 October 2022.

	international)	mycotoxins can induce a wide range of adverse health effects in both animals and human beings, the evidence that they play a role in health problems related to indoor air is extremely weak	
Food allergy (Hair analysis, ALCAT, IgA, IgG)	Insufficient evidence to support the validity of the test method in the general medical community (Guideline issued by recognised professional organisation, Australasian)	Hair analysis, ALCAT, Immunoglobulin G (IgG) to foods are unproven, non evidence-based allergy 'tests' and 'treatments' that are provided by some unorthodox/alternative practitioners. There is currently no stringent government regulation of these methods. Consumers are strongly advised against the use of these tests.	Australasian Society of Clinical Immunology and Allergy (2021) Evidence-based vs Non-evidence based allergy tests and treatments https://www.allergy.org.au/images/pcc/ASCIA_PCC_Evidence-Based_vs_Non_Evidence-Based_Allergy_Tests-Treatments_FAQ_2021.pdf. Accessed 17 May 2022.
Hair loss (hormone tests)	Insufficient evidence to support the validity of the test method in the general medical community (Guideline issued by recognised professional organisation, Australia)	Hormone potentially contributes to hair health, but testing these analytes is not part of the diagnostic pathway for hair loss. Most cases of hair loss do not require pathology tests. Alopecia may be the result of a number of systemic or dermatological condition. Skin biopsy (from the active margin) may be helpful for lesions associated with inflammation or scarring.	RCPA Manual (2022) Alopecia https://www.rcpa.edu.au/Manuals/RCPA-Manual/Clinical-Problems/A/Alopecia. Accessed 17 May 2022.
Heavy metal chelation test (urine analysis)	Insufficient evidence to support the validity of the test method in the general medical community (Guideline issued by recognised professional organisation, US)	Post-chelator challenge urinary metal testing has not been scientifically validated, has no demonstrated benefit, and may be harmful when applied in the assessment and treatment of patients in whom there is concern for metal poisoning. The post-challenge urinary metal test and its application to assess of metal poisoning often leads to unwarranted and prolonged oral and/or intravenous administration of chelating agents. Chelation therapy based on such laboratory values, in addition to being of no benefit to patient outcome, may actually prove harmful.	American College of Medical Toxicology (2010). American College of Medical Toxicology position statement on post-chelator challenge urinary metal testing. *Journal of Medical Toxicology*, *6*(1), 74-75.
Histamine intolerance (diamine oxidase level)	Insufficient evidence to support the validity of the test method in the general medical community (Peer-reviewed literature, CEBM Grade Level 2 – level 2 evidence synthesis)	Histamine intolerance is a disorder associated with an impaired ability to metabolise ingested histamine, causing sensitive response to histamine in food. However, the symptoms are nonspecific an there is currently no consensus or validation of diagnostic tests for this condition. Currently available studies indicate an etiological relationship between diamine oxidase (DAO) deficiency and certain symptoms or disorders related to histamine intolerance. However, the evidence for the validity of blood DAO activity measurements for the diagnosis of histamine intolerance is neither	Comas-Basté, O., Sánchez-Pérez, S., Veciana-Nogués, M. T., Latorre-Moratalla, M., & Vidal-Carou, M. D. C. (2020). Histamine intolerance: The current state of the art. *Biomolecules*, *10*(8), 1181.

		abundant nor conclusive.	
Liver detoxification test (urine metabolites)	Insufficient evidence to support the validity of the test method among the general medical community (Guideline issued by recognised professional organisation, UK)	Liver detoxification tests, used mostly in complementary and alternative medicine (CAM), involves taking several common drugs e.g. paracetamol, aspirin and caffeine and measuring their metabolites in blood and urine afterwards. The amount and ratios of metabolites present are used as an indication of the amount of metabolism taking place in the liver. There is no evidence that the results of liver detoxification tests have any clinical utility in patients who do not have advanced liver disease. Genuine liver damage can be diagnosed using clinical examinations, imaging and other laboratory tests.	Lab Tests Online UK (2022) Unvalidated or misleading laboratory tests, https://labtestsonline.org.uk/tests/unvalidated-or-misleading-laboratory-tests. Accessed 21 May 2022
Mental health nutrition ('Pfeiffer Test')	Insufficient evidence to support the validity of the test method among the general medical community (Peer-reviewed literature, CEBM Grade Level 3 – level 3 evidence synthesis)	The Pfeiffer Test (to be distinguished from Pfeiffer disease) is a test used in CAM to analyse nutritional elements including zinc, copper and zinc-copper ratio in blood, promoted as an indicator of mental disorders such as attention deficit disorder (ADD), attention deficit hyperactivity disorder (ADHD), schizophrenia, Autism, learning disorders and mental disorders. While there is evidence to suggest that people with mental illness are likely to have lower or deficient levels of zinc or a copper-zinc imbalance, the causal link between low zinc and zinc-copper ratio imbalance to mental disorders is not established.	Robberecht, H., Verlaet, A. A., Breynaert, A., De Bruyne, T., & Hermans, N. (2020). Magnesium, iron, zinc, copper and selenium status in attention-deficit/hyperactivity disorder (ADHD). Molecules, 25(19), 4440.
Nagalase test	Insufficient evidence to support the validity of the test method among the general medical community (Guideline issued by recognised professional organisation, international & Australia)	Nagalase is an enzyme produced by cancerous cells. It is an indicator of early or potential cancer. A test for the Nagalase enzyme is most notably used as an indicator of a patients' response to a controversial cancer treatment using Gc Macrophage Activating Factor (GcMAF). There is no credible evidence to show that Gc-MAF can rebuild the immune system or eradicate early stage cancer. Studies that claim successful treatment using GcMAF have since been retracted. The Anticancer Fund and Cancer Council Australia have issued public warnings against using GcMAF as a cancer treatment	Cancer Council Australia (2022) Can GcMAF rebuild the immune system to eradicate early stage cancer? https://www.cancer.org.au/iheard/can-gcmaf-rebuild-the-immune-system-to-eradicate-early-stage-cancer. Accessed 17 May 2022<br>Ugarte, A., Bouche, G., & Meheus, L. [Anticancer Fund] (2014). Inconsistencies and questionable reliability of the publication "immunotherapy of metastatic colorectal cancer with vitamin D-binding protein-derived macrophages-activating, GcMAF" by Yamamoto et al. Cancer Immunology Immunotherapy, 63(12), 1347-8. doi: 10.1007/s00262-014-1587-y

Neurotransmitters urine test (Neurotransmitters in urine)	Insufficient evidence to support the validity of the test method among the general medical community (Peer-reviewed literature, CEBM Grade Level 2 – level 2 evidence synthesis)	Neurotransmitters excreted in the urine may have a place in clinical practice as a biomarker of nervous system function assess disturbances and monitor treatment efficacy for depression, ADHD, and inflammation. However, Urinary neurotransmitter assessment is yet to be accepted as diagnostic for depression or any particular disease or condition. There is variability of baseline measurements. The fraction of monoamines from the nervous system that appear in the urine, and under what conditions, is unclear.	Marc, D. T., Ailts, J. W., Campeau, D. C. A., Bull, M. J., & Olson, K. L. (2011). Neurotransmitters excreted in the urine as biomarkers of nervous system activity: validity and clinical applicability. *Neuroscience & Biobehavioral Reviews*, *35*(3), 635-644.
Small intestinal bacterial overgrowth (SIBO) - Hydrogen & methane breath test	Insufficient evidence to support the validity of the test method among the general medical community (Peer-reviewed literature, CEBM Grade Level 1 - Systematic review)	The lactulose hydrogen breath test is not a standard diagnostic test for SIBO. There is no validated breath test for the diagnosis of SIBO.	Khoshini, R., Dai, S. C., Lezcano, S., & Pimentel, M. (2008). A systematic review of diagnostic tests for small intestinal bacterial overgrowth. *Digestive diseases and sciences*, *53*(6), 1443-1454.

7. Category 4, Subgroup 4B: Non-evidence-based tests with no potential clinical utility (condition tested for is not recognised by the general medical community)

Evidence to support inclusion

There is insufficient evidence to support the condition as established and recognised by the general medical community

Search & inclusion strategy

1. Google Advanced search terms: (*condition - see column 1)* AND diagnos* OR test* OR evidence* The first 30 records were screened for relevance.
2. PubMed and Google Advanced: (*condition - see column 1)* AND diagnos* OR test* OR evidence* The first 30 records in each database were screened for relevance. i) We first prefaced EBM guidelines issued by Australian, Australasian and international professional or governmental organisations. The country or region of origin of the recommendations/guidelines is reported in Column 2. ii) If none were included, we included the peer-reviewed publication with the highest level of evidence (using CEBM Grading) were included. The type of evidence and CEBM grade level used is reported in Column 2. iii) If none were included, we then prefaced web-based consumer information issued by recognised expert health information organisations).

**Medical condition or test purpose; analyte and/or testing method**	**Reason for inclusion & Type of supporting evidence**	**Recommendation or summary of evidence**	**Reference**
Adrenal fatigue (DHEA & Cortisol)	Insufficient evidence to support the condition as established and recognised among the general medical community (Peer-reviewed literature, CEBM	The term 'adrenal fatigue' has been used by some doctors, healthcare providers, and the general media to describe an alleged condition caused by chronic exposure to stressful situations. Despite this, adrenal fatigue has not been recognised by any Endocrinology	Cadegiani, F. A., & Kater, C. E. (2016). Adrenal fatigue does not exist: a systematic review. *BMC endocrine disorders*, *16*(1), 1-16.

	Grade Level 1 - Systematic review)	society, as there is no sufficient evidence for its existence (Cadagiani & Kater, 2016). A systematic review by Cadagiani & Kater (2016) of 58 validated studies show that there is no substantiation that adrenal fatigue is an actual medical condition.	
Intestinal permeability (Lactulose & mannitol; stool analysis)	Insufficient evidence to support the condition as established and recognised among the general medical community (Peer-reviewed literature, CEBM Grade Level 3 – level 3 evidence synthesis)	Intestinal permeability (Leaky gut syndrome) describes a condition in which bacteria and toxins are able to pass through the intestinal wall into the bloodstream. Although barrier dysfunction has been described in a number of disorders, its role in their pathogenesis remains to be defined.	Quigley, E. M. (2016). Leaky gut–concept or clinical entity? *Current opinion in gastroenterology*, *32*(2), 74-79.
Paediatric autoimmune neuropsychiatric disorders (PANDAS) syndrome (Antibody test)	Insufficient evidence to support the condition as established and recognised among the general medical community (Peer-reviewed literature, CEBM Grade Level 2 – level 2 evidence synthesis)	Pediatric autoimmune neuropsychiatric disorders associated with streptococcal infections (PANDAS) is a hypothetical for a subset of children with rapid onset of obsessive-compulsive disorder (OCD) or tic disorders. Although there is a body of evidence that supports the existence of PANDAS and related conditions, it remains a controversial diagnosis. A number of studies investigated the PANDAS hypothesis do support the existence of these conditions. Further studies failed to identify significant differences in specific serum autoantibodies between PANDAS patients and healthy controls. Currently, none of the studied diagnostic approaches of PANDAS is sufficient to confirm the diagnosis.	Wilbur, C., Bitnun, A., Kronenberg, S., Laxer, R. M., Levy, D. M., Logan, W. J., ... & Yeh, E. A. (2019). PANDAS/PANS in childhood: Controversies and evidence. *Paediatrics & child health*, *24*(2), 85-91.
Pyrrole disorder (HPL & neurotoxin)	Insufficient evidence to support the condition as established and recognised among the general medical community (Peer-reviewed literature, CEBM Grade Level 1 - Systematic review)	Pyrrole disorder refers to a mood disorder attributed to elevated levels of pyrroles in the urine, also known as hydroxyhemepyrrolin-2-one (HPL). Hypothetically, the raised pyrrole levels lead to depletions in zinc and vitamin B_6, which, in turn, result in a range of psychiatric disorders, such as schizophrenia, anxiety, and depression.<br>However, elevated HPL is a clinically observed, but poorly researched biomarker with unclear associations with mental disorders.	Warren B, Sarris J, Mulder RT, Rucklidge JJ. Pyroluria: Fact or Fiction? J Altern Complement Med. 2021 May;27(5):407-415. doi: 10.1089/acm.2020.0151. Epub 2021 Apr 27. PMID: 33902305.

[i] Oxford Centre for Evidence-Based Medicine (CEBM): Levels of Evidence (2009) https://www.cebm.ox.ac.uk/resources/levels-of-evidence/oxford-centre-for-evidence-based-medicine-levels-of-evidence-march-2009 . Accessed 2 May 2022
