# Supplementary figures and images for: Direct-to-consumer tests advertised online in Australia and their implications for medical overuse: systematic online review and a typology of clinical utility
Source: BMJ Open. 2023 Dec 27;13(12):e074205. doi: 10.1136/bmjopen-2023-074205 (PMC10759116; doi:10.1136/bmjopen-2023-074205)

Supplemental Figure 1. Levels of distinctions in analysis

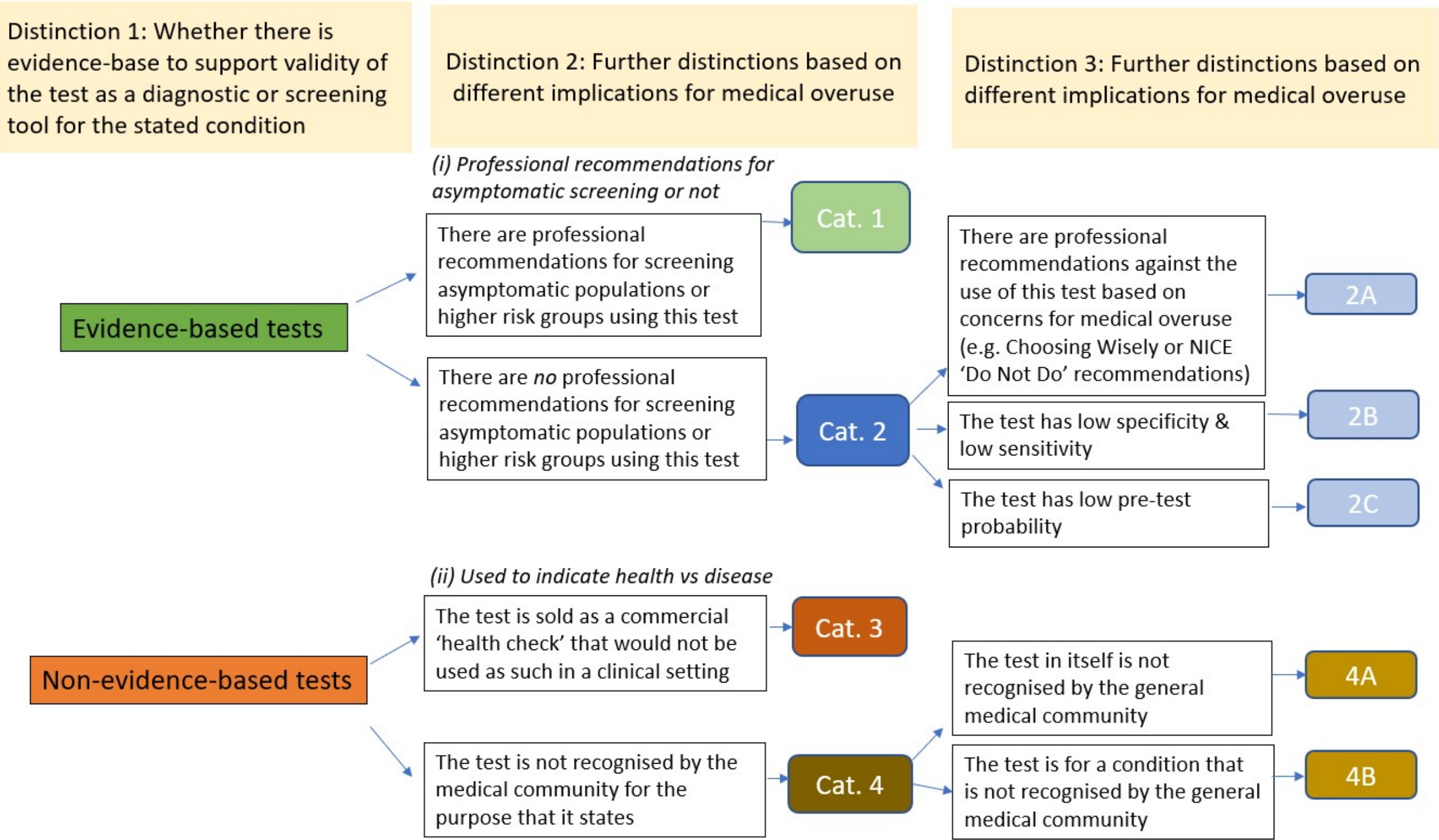

Supplement: Supplementary data [file bmjopen-2023-074205supp002.pdf]

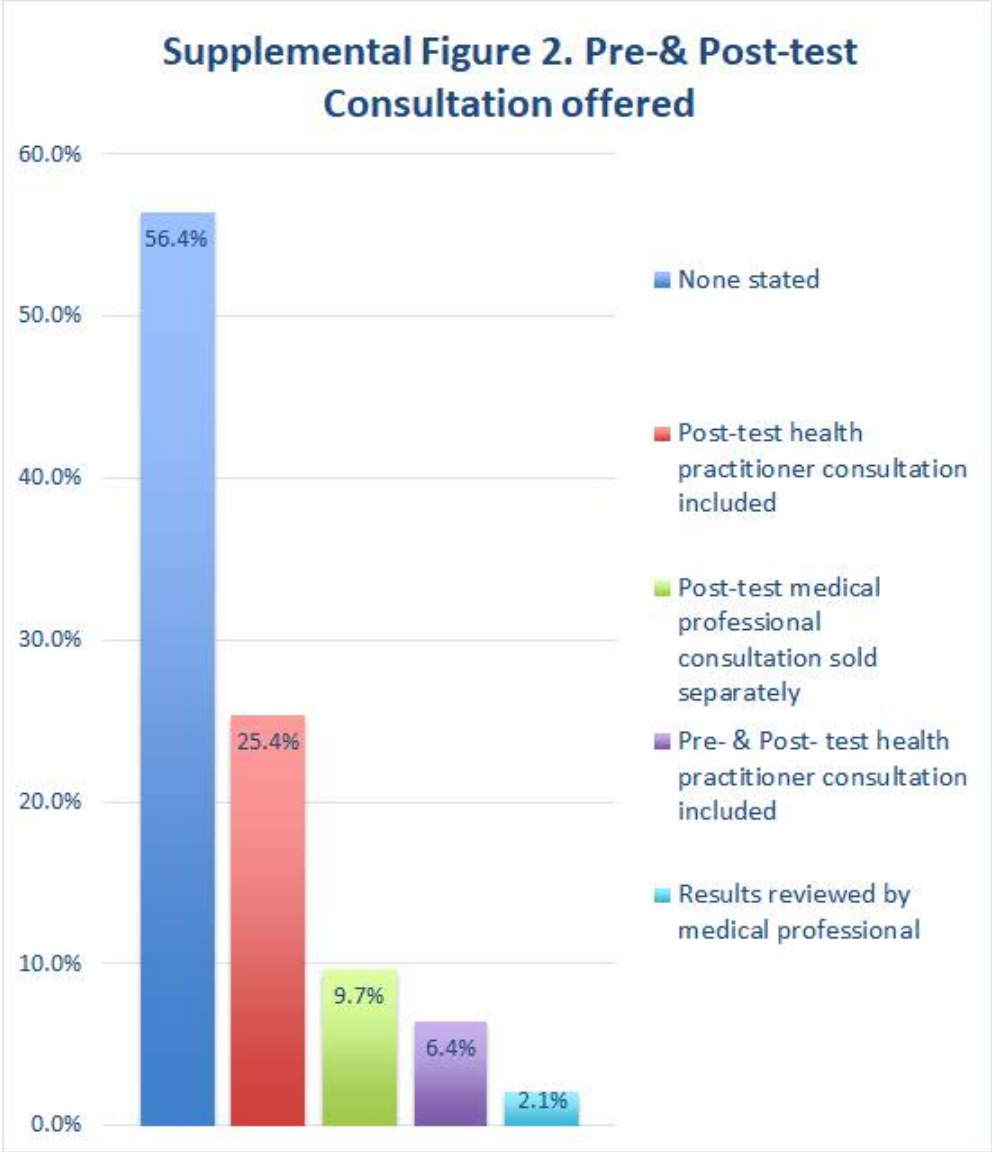

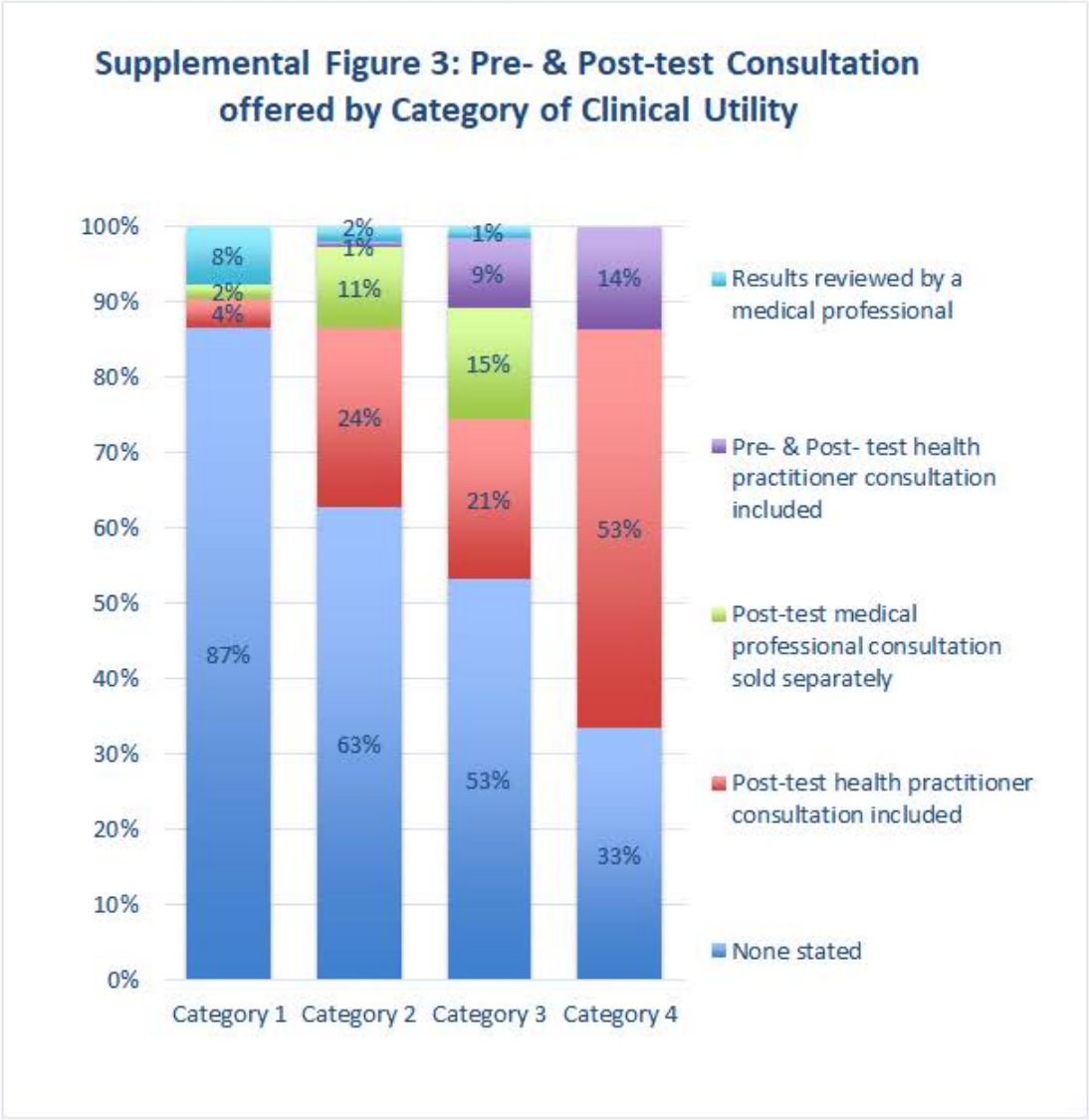

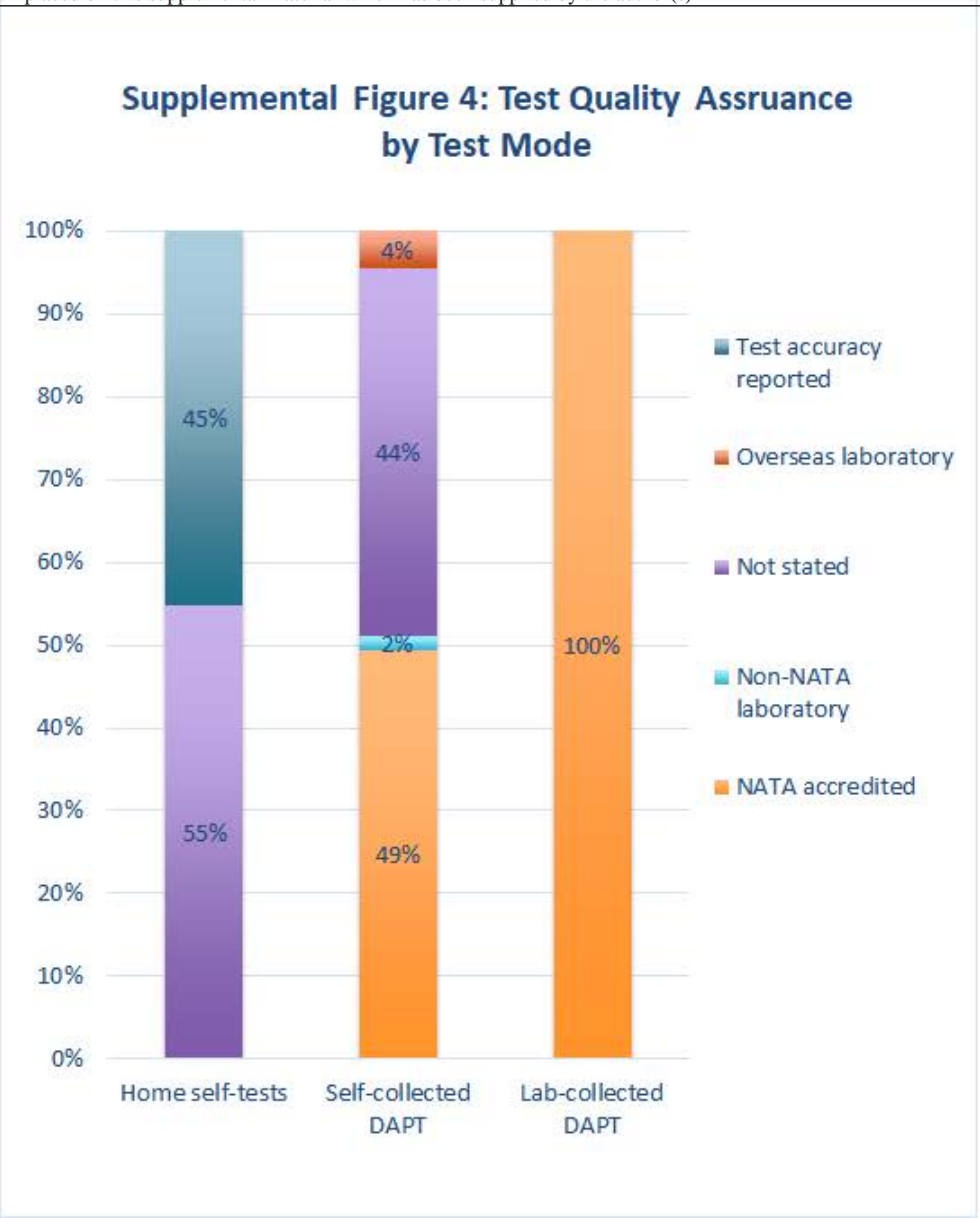

Supplement: Supplementary data [file bmjopen-2023-074205supp003.pdf]
