# Supplementary material for: Direct-to-consumer tests advertised online in Australia and their implications for medical overuse: systematic online review and a typology of clinical utility
Source: BMJ Open. 2023 Dec 27;13(12):e074205. doi: 10.1136/bmjopen-2023-074205 (PMC10759116; doi:10.1136/bmjopen-2023-074205)
Supplement: Author's manuscript [file bmjopen-2023-074205.draft_revisions.pdf]

BMJ Open is committed to open peer review. As part of this commitment we make the peer review history of every article we publish publicly available.

When an article is published we post the peer reviewers' comments and the authors' responses online. We also post the versions of the paper that were used during peer review. These are the versions that the peer review comments apply to.

The versions of the paper that follow are the versions that were submitted during the peer review process. They are not the versions of record or the final published versions. They should not be cited or distributed as the published version of this manuscript.

BMJ Open is an open access journal and the full, final, typeset and author-corrected version of record of the manuscript is available on our site with no access controls, subscription charges or pay-per-view fees (http://bmjopen.bmj.com).

If you have any questions on BMJ Open's open peer review process please email info.bmjopen@bmj.com

BMJ Open

Direct-to-consumer tests advertised online in Australia: Clinical utility and implications for medical overuse

Journal:	*BMJ Open*
Manuscript ID	bmjopen-2023-074205
Article Type:	Original research
Date Submitted by the Author:	04-Apr-2023
Complete List of Authors:	Shih, Patti; University of Wollongong, School of Health & Society<br>O'Shaughnessy, Pauline; University of Wollongong, School of Mathematics and Applied Statistics<br>Carter, Stacy M; University of Wollongong,<br>Stanaway, Fiona; University of Sydney, School of Public Health<br>Horvath, Andrea ; NSW Health Pathology; University of New South Wales<br>Langguth, Daman; Wesley Hospital, Sullivan Nicolaides Pathology<br>Saad, Mirette; Australian Clinical Labs Victorian Central Laboratory Headquarters<br>St John, Andrew; Drajon Health, ; ARC Consulting,<br>Bell, Katy; The University of Sydney Faculty of Medicine and Health, School of Public Health; The University of Sydney
Keywords:	HEALTH SERVICES ADMINISTRATION & MANAGEMENT, PATHOLOGY, Decision Making, Health Services Accessibility

SCHOLARONE™
Manuscripts

I, the Submitting Author has the right to grant and does grant on behalf of all authors of the Work (as defined in the below author licence), an exclusive licence and/or a non-exclusive licence for contributions from authors who are: i) UK Crown employees; ii) where BMJ has agreed a CC-BY licence shall apply, and/or iii) in accordance with the terms applicable for US Federal Government officers or employees acting as part of their official duties; on a worldwide, perpetual, irrevocable, royalty-free basis to BMJ Publishing Group Ltd ("BMJ") its licensees and where the relevant Journal is co-owned by BMJ to the co-owners of the Journal, to publish the Work in this journal and any other BMJ products and to exploit all rights, as set out in our licence.

The Submitting Author accepts and understands that any supply made under these terms is made by BMJ to the Submitting Author unless you are acting as an employee on behalf of your employer or a postgraduate student of an affiliated institution which is paying any applicable article publishing charge ("APC") for Open Access articles. Where the Submitting Author wishes to make the Work available on an Open Access basis (and intends to pay the relevant APC), the terms of reuse of such Open Access shall be governed by a Creative Commons licence – details of these licences and which Creative Commons licence will apply to this Work are set out in our licence referred to above.

Other than as permitted in any relevant BMJ Author's Self Archiving Policies, I confirm this Work has not been accepted for publication elsewhere, is not being considered for publication elsewhere and does not duplicate material already published. I confirm all authors consent to publication of this Work and authorise the granting of this licence.

For peer review only

Direct-to-consumer tests advertised online in Australia: Clinical utility and implications for medical overuse

Abstract

Objectives: To map the range and variety of direct-to-consumer (DTC) tests advertised online in Australia and analyse their potential clinical utility and implications for medical overuse.

Design: Systematic online search of DTC test products in Google and Google Shopping. DTC test advertisements data were collected, and analysed to develop a typology of potential clinical utility of the tests at population level, assessing their potential benefits and harms using available evidence, informed by concepts of medical overuse.

Results: We identified 484 DTC tests (103 unique products), ranging from AU$12.99 to AU$1947 in cost. Using our typology, we assigned the tests into one of four categories: tests with potential clinical utility (10.7%); tests with limited clinical utility (30.6%); non-evidence-based commercial 'health checks' (41.9%); and tests whose methods and/or target conditions are not recognised by the general medical community (16.7%). Of the products identified, 56% did not state that they offered pre- or post-test consultation, and 51% did not report analytical performance of the test or laboratory accreditation.

Conclusions: This first-in-Australia study shows most DTC tests sold online have low potential clinical utility, with healthy consumers constituting the main target market. Harms may be caused by overdiagnosis, high rates of false positives, and treatment decisions led by non-evidence-based tests, as well as financial costs of unnecessary and inappropriate testing. Regulatory mechanisms should demand a higher standard of evidence of clinical utility and efficacy for DTC tests. Better transparency and reporting of health outcomes, and the development of decision-support resources for consumers are needed.

For peer review only - http://bmjopen.bmj.com/site/about/guidelines.xhtml

Strengths & limitations to this study

- This is the first-in-Australia study providing a comprehensive review and analysis of DTC tests advertised online
- The conceptual focus on the clinical utility of DTC tests provides a nuanced and critical angle on their implications for medical overuse and potential benefits and harms for consumers
- The DTC tests included in this time-specific search may not be exhaustive, as product availability may change over time, and others may be advertised in different platforms or sold in pharmacies.
- DTC tests offered by private commercial clinics but which are not advertised as a directly-purchasable item online, as is often the case for genetic and fertility tests, were also not included.
- This is an overview of products available; data extraction did not include an in-depth qualitative assessment of the textual information in each advertisement.

For peer review only - http://bmjopen.bmj.com/site/about/guidelines.xhtml

1. Introduction

Direct-to-consumer (DTC) tests are commercially marketed pathology tests, purchased by members of the public,[1, 2] who pay for and decide on which test to access and when. DTC testing does not require the recommendation or referral from a consulting doctor, nor the facilitation of a public health program. This makes them distinct from home collection kits that are part of screening programs (e.g. bowel cancer screening), pandemic responses (e.g. COVID-19 rapid antigen tests (RATs)), or self-monitoring tests for patients already diagnosed with a condition or under clinical management (e.g. diabetes self-monitoring).

The size of the DTC test market is rapidly growing, expanding from a reported US$15.28 million per annum in 2010 to over US$352 million in 2020.[3] As part of a greater shift towards more individualised and consumerised models of healthcare, DTC testing promises the opportunity for more proactive self-care and personal autonomy.[4, 5] No longer just reliant on screening programs or doctors' recommendations, consumers can initiate and obtain pathology tests quickly and conveniently to inform early disease prevention or enable timely management decisions. Bypassing clinical visits can allow more privacy and confidentiality around testing, and avoids the additional cost and logistics of travel and administration.[5]

Studies of DTC tests in the US and UK show a wide variety of diagnostic, screening and risk monitoring tests being available for direct purchase, suggesting consumers with and without symptoms are targeted.[6, 7 8] However, only 15% of the DTC screening tests found in the US were supported by evidence-based recommendations, and in the UK, only one out of 24 companies offering allergy testing used approved methods from an accredited laboratory.[8] These findings suggest the need to scrutinise the *clinical utility* of DTC tests: whether the product leads to useful clinical decision-making and improved health outcomes. Clinical utility depends on getting the right test for the right person at the right time.[9] This is

For peer review only - http://bmjopen.bmj.com/site/about/guidelines.xhtml

determined by a variety of factors, such as an individual's medical history, symptoms (if any), and pre-test probability for the target condition. Equally relevant are the reliability and validity of the testing method, the quality of the in-vitro device, and the credibility of the laboratory. When products are directly-marketed, consumers may be making test decisions without the adequate opportunity for discussion and guidance from their doctors.[10] Indeed, several studies show that the quality and reliability of information on websites promoting tests varies,[6, 11, 12] which can expose consumers to the harms of choosing inappropriate and/or unnecessary tests.

An often under-analysed harm related to pathology tests of limited clinical utility is medical overuse: healthcare that is either harmful or does not lead to improved health outcomes.[13, 14] This includes using tests inappropriately or unnecessarily, such as testing low-risk and asymptomatic populations outside of clinical recommendations. This can drive the unnecessary diagnosis of early or slow-progressing diseases that would not have harmed the patient in their lifetime (overdiagnosis), and instead lead to treatments that produce no significant benefit (overtreatment).[13, 14] Testing, whether using home testing kits or at a laboratory, is resource intensive and produces medical waste.[15] Testing demands further clinical resources such as analysis and interpretation, and decision-making based on results, whether positive or negative.[16, 17] Test results can also trigger downstream testing and unjustified treatments that generate further costs and resource-use in the health system, such as unnecessary General Practitioner (GP) visits.[18, 19] When testing decisions are not clinically justified or do not lead to improvement in health outcomes, they are a form of 'low-value care', creating waste and additional burden to the healthcare system.[20] Although it is well-recognised that medical overuse occurs even in clinician-guided settings,[13, 21] it may be exacerbated in a DTC context where consumers may be making testing decisions with little support or balanced information.[17]

For peer review only

Considerable critical attention has already been paid to the questionable clinical utility of the growing DTC genetic testing and fertility treatment industry.[22-24] For example, while genetic tests for conditions such as Alzheimer's disease produce data that predict a person's risk of eventually developing the condition, where prevention strategies are limited, testing can cause psychosocial distress to healthy people.[25] The results of the anti-Mullerian hormone ('Egg Timer') test measuring ovarian reserve are unreliable as a basis for making family planning decisions in the future, as the test is only useful for patients undergoing a current fertility treatment.[12] Typically, these better-known DTC genetic and fertility tests are offered by private clinics that include a suite of services and consultations before tests are chosen and performed 'in-house'.

An emerging trend in DTC testing that has had limited exploration is the 'direct purchase' online testing market: pathology test products and services advertised widely on the internet, and immediately purchasable in a single online transaction. Broadly, there are three types of directly-purchased DTC tests: 1) *Home self-tests* - tests kits used by consumers themselves producing immediate results for their own interpretation; 2) *Self-collected direct accessed pathology tests (DAPTs)* - a pathology test requested from a commercial company on behalf of a consumer who purchased the test, rather than requested by a doctor after consultation and discussion, with samples collected at home and mailed into a laboratory; or 3) *Lab-collected DAPTs*, again purchased directly by a consumer, but with samples collected in a laboratory. Home self-tests and DAPTs signal different levels of involvement of laboratory professionals and have different regulatory requirements depending on the jurisdiction. For example, in Australia, home self-tests are regulated by the Therapeutic Goods Administration (TGA), whereas laboratory-tested DAPTs are regulated by the National Association of Testing Authorities (NATA]). The commonality is that consumers are the primary decision-makers in testing and purchasing decisions. While some DTC testing services do offer professional consultations as an inclusion or as an add-on purchase, testing

For peer review only - http://bmjopen.bmj.com/site/about/guidelines.xhtml

decisions are already made before an opportunity to discuss and deliberate, if at all, about the personal values and expectations of the consumer in relation to taking a specific test.[26]

Currently, there is limited research available on DTC tests available in Australia, nor has there been much assessment of the potential benefits and harms of the different products available. This first-in-Australia study reviews DTC tests advertised online, with a focus on their clinical utility and implications for medical overuse. The research questions for this study are: 1) How many and what variety of products are advertised and available to Australian consumers online? 2) What are their implications for medical overuse?

2. Methods

The study took place in two stages. Firstly, a systematic search of DTC test products advertised online was conducted; Secondly, the data collected was developed into a typology of potential clinical utility of the product, which was analysed based on the potential benefits and harms of the test at a population level, informed by concepts of medical overuse.

Stage 1 - Systematic online search: A pilot search was conducted in Google and Google Shopping by two researchers independently in October 2020. These engines were chosen because the majority of internet searches for product purchases in Australia are conducted on these platforms. The pilot search identified the three modes of DTC tests (home self-tests, self-collected DAPT and lab-collected DAPT) and a range of medical conditions and/or types of tests. This helped refine the search terms used in an updated search (Table 1), taking place between June and August 2021. We allowed snowball inclusion of new items between August - December 2021, whereby additional products that met the study inclusion criteria were added if they appeared on the same websites advertising already-included products. We reviewed the first 20 pages (10 results per page, totalling 200 results) from each search. We excluded sites that were not relevant, and any duplicate results. We created a data extraction table to chart data relevant for the assessment of clinical utility and medical overuse, and collected these data from the website of each included product: name of product, condition

For peer review only - http://bmjopen.bmj.com/site/about/guidelines.xhtml

and/or analyte tested for, specimen sampled, mode of the test, cost in Australian dollars (including shipping), whether the product offers any pre- or post-test consultations; and any indicators of the quality assurance of the tests (for home self-tests, this is indicated by information about the diagnostic accuracy of the test, expressed as clinical sensitivity and specificity metrics; and for DAPT, this is indicated by any information on the accreditation of nominated laboratories or individual tests).

Table 1: Data search and selection strategy

Search strategy	Description	
Search string 1	Home self-tests & Self-collected pathology tests	Search: "self test", "self diagnosis","home test", "home diagnosis", “home screen”, “self screen”,
Search string 2	Direct access pathology & Self-collected pathology tests	Search string 2: “direct access test*”, “self request* pathology”, “private blood test*”, “private medical test”, “online blood test”.
Search inclusions & exclusions		The published region was set in Australia. The search excluded the term COVID, and .gov and.edu domains, to omit any screening tests that are offered by government screening programs.
Selection inclusion	To be included in the study:<br>For all tests:<br>i. The tests are intended to indicate the presence of disease or conditions (e.g. diagnose a disease), or for general well-being purposes (e.g. tests that purport to indicate level of hydration, vitamins, hormones)<br>ii. The product can be shipped to an Australian address<br>iii. A test must be a purchasable product, itemised and displayed in AUD that is able to be purchased via an online transaction<br>• Home self-tests: must include an in-vitro device that collects a human tissue sample (e.g. a lancet or container for blood, urine, saliva, faecal material); samples are processed at home (e.g. testing strip)<br>• DAPT self-collected: must include an in-vitro device that collects a human tissue sample (e.g. a lancet or container for blood, urine, saliva, faecal material); samples are sent to be processed at a laboratory<br>• DAPT lab-collected services: must be a pathology request for a named biomarker or analyte that is tested in a laboratory	

For peer review only - http://bmjopen.bmj.com/site/about/guidelines.xhtml

Selection exclusion	The product is excluded if it is:<br>i. Not for testing human samples (e.g. animals, soil, water)<br>ii. Intended for use by professionals or clinics, e.g. doctors, hospitals or laboratories<br>iii. Used or purchased with a separate product (e.g. used with a meter or reader or as part of a health coaching program)<br>iv. Funded as part of a government or health group screening program<br>v. For indicating the presence of illicit drugs or alcohol, and non-health related purposes (e.g. paternity and ancestry)<br>vi. For indicating pregnancy, monitoring a pre-existing condition, testing for SARS-CoV-2 infection

Stage 2 - Developing a typology of clinical utility: As an emerging new area of research, there were no available or comparable typologies of clinical utility that could be applied as a framework to the DTC context. We used an abductive analytical approach to make sense of the data, which combines both inductive and deductive data analysis.[27] Inductive approach allows key concepts to emerge from data itself; and the application of pre-existing theories and knowledge deductively forms a more structured understanding of data.[27] The analysis was led by the first author with input from the multidisciplinary authorship team, which included experts in medical overuse (KB, SC, ARH) and pathology (ARH, DL, MS, ASJ).). The data was firstly viewed as a whole, and then grouped and sorted into broader categories based on the key similarities and distinctions over several iterations. The grouping and sorting process was informed by pre-existing concepts and our own prior knowledge of the medical overuse literature, especially in relation to the overuse of pathology tests. The following principles from the medical overuse literature most relevant for pathology tests were used to guide the analysis:

- The balance of potential harm and benefit of the test is based on the assumption that a consumer is making the testing decision on their own; but the potential harm of the test itself is analysed at a population level.

For peer review only - http://bmjopen.bmj.com/site/about/guidelines.xhtml

- The scientific and analytical validity of the test as appropriate for the condition and/or purpose stated is based on published literature supporting scientific evidence, selected using the hierarchy of evidence as determined by the Oxford Centre for Evidence-based Medicine (OCEBM).[28]

- We prioritised any information identified by existing recommendations for or against by professional organisations, especially those developed to prevent overdiagnosis (e.g. NICE, Choosing Wisely).

We moved iteratively back and forth between data and theory to change or add new inclusion/exclusion criteria to the groupings until no new categories emerged, and there was consensus between the authors (see Supplemental Figure 1). The allocation of tests into each category was supported by a literature search of evidence-based medicine (see Supplement 1). The final categories were determined by the following levels of distinctions: The first key distinction in the data made was whether there is sufficient evidence-base to support the validity of the test as a diagnostic or screening tool for the stated condition it tests for. Secondly, within the evidence-based tests, there was a further distinction between those with and without a professional recommendation for screening the condition among specific populations. In the non-evidence-based tests, there was a distinction between those that aimed at testing for a disease and those that aimed at indicating health. Thirdly, within the evidence-based tests with no recommendations for screening, tests that are 'recommended against' by known professional guidelines aimed at preventing medical overuse were highlighted, followed by tests that have other medical overuse concerns, for example if the test method itself has low specificity or sensitivity, or that the condition being tested for has low pre-test probability. Within the non-evidence-based tests for disease, a distinction was made between tests that were non-evidence based in themselves, and tests used to diagnose conditions that were not recognised by the general medical community. In total, four categories emerged, with two of the categories (2 and 4) having three and two subcategories respectively.

For peer review only - http://bmjopen.bmj.com/site/about/guidelines.xhtml

<insert Supplemental Figure 1>

3. Findings

A total of 484 DTC tests were identified. Collectively these targeted 103 unique medical conditions, analytes or test products. Tests ranged from AU$12.99 to AU$1947 in cost. There were 177 (36.6%) home self-tests, 65 (13%) self-collected DAPT, and 242 (51%) lab-collected DAPT.

3.1. Categories of clinical utility

1) Category 1: Tests with potential clinical utility (10.7% of total)

Just over 10% of total products were allocated to Category 1 (Table 2). These were evidence-based tests recommended for screening in specific population groups by a recognised professional medical organisation, determined by a reported evidence-based medicine (EBM) approach. The most common examples were tests for sexually transmitted infections (STI), for which stigma contributes to barriers to clinical care, especially among communities at higher risk. In our study, DTC tests for STI tended to come in the form of home self-testing kits, which are lower in cost compared to DAPT and more likely to report test accuracy (Fig. 4). Studies in Australia and internationally show that undertested at-risk populations are more likely to use online and home-based testing for STI than at primary care settings.[29 30] DTC tests in this category may improve the rate of beneficial diagnosis and treatment in high-risk populations, especially those with barriers to clinical care. However, the test will have limited utility for asymptomatic and low-risk populations outside of populations recommended for routine testing. Nevertheless, clinical follow-up is still needed to ensure timely treatment.

For peer review only

This is especially notable since Category 1 tests are the least likely to offer pre- and post-test consultation.

Table 2: Category 1 results

Inclusion criteria	Target condition or test purpose	Key analyte or testing method	Mode of test[#]				Min. cost	Max. cost
			H	S	L	TN		
An established and recognised part of the diagnostic, screening or risk assessment pathway for the named medical condition or risk factor of a condition the product purports to test for.<br><br>Recommended for screening in specific population groups by a recognised professional medical organisation, determined by a reported evidence-based medicine (EBM) approach.	Bowel cancer	Faecal occult blood	0	1	1	**2**	69.00	129.00
	Cardiovascular disease risk factors	Lipoprotein	0	0	3	**3**	198.00	330.00
	Chlamydia	Chlamydia trachomatis antigen	4	0	0	**4**	24.95	55.29
	Cholesterol level	Blood cholesterol	1	1	1	**3**	45.00	84.00
	Diabetes	HbA1c, glucose	0	2	4	**6**	35.00	78.00
	Gonorrhoea	Neisseria gonorrhoea antigen	3	0	0	**3**	34.95	56.71
	Hepatitis B	Hepatitis B surface antigen	4	0	0	**4**	24.95	56.71
	Hepatitis C	Hepatitis C virus antibody	2	0	0	**2**	56.71	92.40
	HIV	HIV-1 & HIV-2 antibody	11	0	0	**11**	34.95	138.00
	Sexually transmitted diseases - multiple tests	Chlamydia antigen, Gonorrhoea antigen, Hepatitis B antigen, Syphilis	2	0	7	**9**	64.00	188.00
	Syphilis	Treponemal Antibody	4	0	0	**4**	24.95	56.71
	Trichomoniasis infection	Trichomonas vaginalis lipophosphoglycan antibody	1	0	0	**1**	56.71	

[#]Mode of access: H=home self-tests, S= self-collected DAPT, L=Lab-collected DAPT, TN=total number).

2) *Category 2: Tests with limited clinical utility (30.6%)*

While tests in Category 2 (Table 3) have a legitimate role in diagnosis or treatment in clinical settings for the condition they target, the context in which these are marketed as DTC tests bring up several concerns for medical overuse: Firstly, about half in this category (designated

For peer review only

For peer review only - http://bmjopen.bmj.com/site/about/guidelines.xhtml

as Subgroup 2A, 19.4% of total), were tests identified by well-known guidelines that aim to reduce medical overuse, such as the Choosing Wisely 'Do Not Do' recommendations and the, NICE 'Do Not Do' Recommendation, and 'Screening tests of unproven benefit' identified by the Royal Australasian College of General Practitioners (RACGP)'s 'Guidelines for preventative activities in general practice'.[31-33] Examples include prostate-specific antigen and MTHFR gene testing. Other tests in this category were more likely to generate high false positive rates because of the limited accuracy of the testing method for the target condition (Subgroup 2B, 9.3% of total). Examples include urine dipsticks for kidney and liver disease, which are often used in point-of-care screening before further investigation; whereas when used by consumers at home, false positive results may cause unnecessary distress and trigger further GP visits. Thirdly, a small proportion were tests for conditions with low pre-test probability in the general population[34] (Subgroup 2C, 1.9%). This means the potential for producing high rates of false positives or misattributed diagnosis. For example, an IgG test for Cytomegalovirus is of limited utility because a high proportion of the general population will carry antibodies without clinically significant disease. Even though the test may correctly show positive results from past vaccination or infection, it has limited utility in diagnosis, especially if symptoms are vague or within normal limits.

Table 3: Category 2 results

Inclusion criteria	Target condition or test purpose	Key analyte or testing method	Mode of test[#]				Min. cost	Max. cost
			H	S	L	TN		
Subgroup 2A *(n=94, 19.4% of total)*								
Identified as potentially contributing to medical overuse in	Alzheimer's disease	APOE genotype	0	1	1	**2**	165.00	270.00
	Arthritis	Rheumatoid Factor	0	0	2	**2**	44.00	130.00
	Autoimmune disease	HLA-B27 antibody	0	0	2	**2**	69.00	120.00

For peer review only - http://bmjopen.bmj.com/site/about/guidelines.xhtml

certain populations by professionally recognised and evidence-based medicine (EBM) recommendations that aim to prevent medical overuse (e.g. Choosing Wisely, NICE 'Do Not Do' Recommendations)	Cancer markers	Carcinoembryonic antigen (CEA)	0	0	3	**3**	80.00	100.00
	Cancer risk factors	Glutathione S-transferases genes	0	0	2	**2**	313.00	142.00
	Cardiovascular disease risk factors	Homocysteine	0	0	1	**1**	160.00	
	Cardiovascular disease risk factors	MTHFR & APOE genetic test	0	1	0	**1**	199.00	
	Coeliac disease	HLA gene typing	0	0	1	**1**	169.00	
	Food allergy	IgE	0	2	5	**7**	109.00	430.00
	Genital herpes	Antibodies	3	0	0	**3**	63.00	140.40
	Haemochromatosis	HFE gene mutation	0	0	2	**2**	69.00	159.00
	Heavy metal exposure	Heavy metal in blood	0	0	3	**3**	118.00	189.00
	Immune disorders	Immunogenetics	0	1	0	**1**	440.00	
	Insulin resistance	Fasting glucose, fasting Insulin	0	0	2	**2**	69.00	190.00
	Iron deficiency	Iron studies	0	1	8	**9**	58.00	318.00
	Low-grade Inflammation	High sensitivity CRP	0	0	1	**1**	142.00	
	MTHFR gene mutation	MTHFR gene mutation	0	4	5	**9**	50.00	520.00
	Neuro genetic disorders	Neurogenetics	0	1	0	**1**	647.00	
	Prostate cancer	Prostate-specific antigen	0	0	2	**2**	39.00	40.00
	Thyroid disease	Thyroid hormones	1	7	15	**23**	35.00	328.00
	Urinary tract infection	Urine culture & microscopy	0	0	3	**3**	36.00	50.00
	Vitamin B deficiency	Vitamin B12, B6, B9	0	0	6	**6**	65.00	298.00
	Vitamin D deficiency	Vitamin D	2	4	1	**7**	35.00	175.00
	Vitamin K deficiency	Vitamin K	0	1	0	**1**	280.00	
**Subgroup 2B** *(n=45, 9.3% of total)*								
The testing method used has low sensitivity (limited	Blood clotting	Prothrombin time	0	0	1	**1**	55.00	
	Chronic fatigue	Iron, Vitamin B12, thyroid hormones	0	0	1	**1**	145.00	

For peer review only

ability to correctly predict disease) or low specificity (limited ability to correctly rule out disease) or is not reliable as a diagnostic test when used on its own. To reach an accurate diagnosis for the named condition, the gold standard diagnosis, and/or follow-up or parallel tests would be required.	Coeliac disease	IgA & IgG	0	0	4	**4**	69.00	232.00
	Gastrointestinal candida	IgG & IgM	0	1	0	**1**	240.00	
	Giardia	Lambia rapid antigen test	1	0	0	**1**	106.80	
	Helicobacter pylori	Stool antigen	1	2	1	**4**	90.00	125.00
	Inflammatory bowel disease	Faecal calprotectin	0	1	4	**5**	75.00	95.00
	Kidney disease	Albumin/ Creatinine Ratio dipstick	2	0	2	**4**	39.00	69.60
	Liver disease	Bilirubin dipstick	1	1	2	**4**	35.99	65.00
	Measles, mumps, rubella serology (indicating past infection)	Antibodies	0	0	4	**4**	49.00	90.00
	Pancreatic insufficiency	Faecal pancreatic elastase 1	0	0	1	**1**	89.00	
	Pancreatitis	Lipase, amylase	0	0	2	**2**	59.00	62.00
	Urinary tract infection	Urine dipstick (Nitrite and leukocyte esterase)	10	0	0	**10**	25.99	215.00
	Vaginal infection	Vaginal pH	6	0	0	**6**	12.99	14.95
**Subgroup 2C** ***(n=9, 1.9% of total)***								
The test is for a medical condition that has a low pre-test probability. Investigating clinically significant disease without clinician guidance or assessment of symptoms and medical history can drive false positive results.	Cytomegalovirus (CMV)	IgC & IgM antibody	0	0	1	**1**	59.00	
	Epstein-Barr virus (EBV)	EBV Antibody	0	0	1	**1**	59.00	
	Glucose-6-Phosphate-Dehydrogenase (G6PD) deficiency	G6PD enzyme assay	0	0	1	**1**	299.00	
	Lyme disease	Borrelia antibody	0	0	5	**5**	59.00	903.00
	Rotavirus	Rapid antigen test	1	0	0	**1**	92.40	

[#]Mode of access: H=home self-tests, S= self-collected DAPT, L=Lab-collected DAPT, TN=total number).

3) Category 3: Non-evidence-based commercial 'health checks' (41.9%)

For peer review only

Category 3 (Table 4) were non-evidence-based commercial 'health checks', making up the largest proportion of all tests in this study, at 41.9% of total. This finding suggest that healthy people form the largest target of the DTC test marketing. These tests may often be justifiable if offered in a clinical setting as part of investigating symptoms or disease monitoring (e.g. testosterone hormone tests for hypogonadism). However there is limited evidence to support their clinical utility as evaluative tests for health status among healthy populations.[35] A health check is potentially useful if clinically relevant abnormalities are detected. However, when a test is not used for investigating symptoms of a known medical condition, there are no agreed protocols or harmonised range to interpret whether the test results fall into a healthy or pathological range for a particular individual (the reference interval), which may vary between laboratories. Testing asymptomatic and low-risk populations can sometimes result in the incidental detection of conditions or risk factors that may not benefit from diagnosis or treatment.

For peer review only

Table 4: Category 3 results

Inclusion criteria	Target condition or test purpose	Key analyte or testing method	Mode of test#				Min. cost	Max. cost
			H	S	L	TN		
Evaluates normally existing biomarkers or health status in healthy populations; Does not aim at detecting, diagnosing or monitoring of a specific disease or medical condition	Biological age	Glycans	0	1	0	**1**	599.00	
	Bone health	Serum alkaline, phosphatase, calcium, potassium	0	0	2	**2**	80.00	125.00
	Coenzyme Q10 profile	Coenzyme Q10 level	0	0	1	**1**	180.00	
	Cytokine profile	Cytokine level	0	0	1	**1**	425.00	
	Fertility status (male)	Semen analysis	0	0	1	**1**	75.00	

For peer review only - http://bmjopen.bmj.com/site/about/guidelines.xhtml

	Gastrointestinal function	Microbiome analysis	0	11	1	**12**	109.00	1,199.0 0
	Gastrointestinal function	Stool analysis	0	14	0	**14**	120.00	490.00
	Health & wellness profile	Panel consisting of tests for more than one of: Full blood count, hormones, organ function, vitamins & minerals	1	0	48	**49**	18.95	749.00
	Hormone profile - female fertility status	Panel consisting of tests for more than one of: Follicle Stimulating Hormone, luteinising hormone, oestradiol, progesterone, testosterone	0	1	13	**14**	59.00	165.00
	Hormone profile - female health	Panel consisting of tests for more than one of: DHEAs, estrone, SHBG, oestradiol, estriol, progesterone, testosterone, androstenedione	0	11	1	**12**	54.00	410.00
	Hormone profile - general health	Panel consisting of tests for more than one of: adrenal, growth, thyroid & reproductive hormones	0	10	9	**19**	59.00	239.00
	Hormone profile - male health	Panel consisting of tests for more	0	8	3	**11**	50.00	270.00

For peer review only

For peer review only - http://bmjopen.bmj.com/site/about/guidelines.xhtml

		than one of: DHEA, estrone, oestradiol, testosterone, SHBG, androstenedione						
	Hormone profile - menopause	Panel consisting of tests for more than one of: oestradiol, estriol, estrone, DHEA	0	2	2	**4**	81.00	179.00
	Hormone profile - ovarian reserve	Anti-Mullerian Hormone	0	0	3	**3**	98.00	299.00
	Hormone profile - sleep quality	Melatonin & cortisol	0	5	1	**6**	909.00	189.00
	Hormone profile - sports & fitness performance	Panel consisting tests for more than one of: Growth & adrenal hormones	0	1	14	**15**	59.00	239.00
	Hormone profile - stress	Salivary cortisol	0	10	0	**10**	23.00	165.00
	Nutrigenomic profile	Genetics	0	2	0	**2**	249.00	550.00
	Nutritional status	Amino acids	0	2	0	**2**	270.00	370.00
	Nutritional status	Ammonia	0	0	1	**1**	153.00	
	Nutritional status	Chromium	0	0	1	**1**	108.00	
	Nutritional status	Copper	0	0	2	**2**	85.00	108.00
	Nutritional status	Essential Fatty Acids	0	1	1	**2**	89.00	225.00
	Nutritional status	Glutathione	0	0	1	**1**	335.00	
	Nutritional status	Magnesium	0	0	1	**1**	138.00	

For peer review only - http://bmjopen.bmj.com/site/about/guidelines.xhtml

	Nutritional status	Organic acids	0	8	0	**8**	160.00	597.00
	Nutritional status	Selenium	0	0	1	**1**	108.00	
	Nutritional status	Iodine	0	1	0	**1**	155.00	
	Nutritional status	Zinc	0	0	2	**2**	75.00	170.00
	Secretor status (blood typing)	Antigens	1	1	0	**2**	89.95	

#Mode of access: H=home self-tests, S= self-collected DAPT, L=Lab-collected DAPT, TN=total number).

4) Tests whose methods and/or target condition are not recognised by the general medical community (16.7%)

While Category 4 tests (Table 5) were also non-evidence-based (16.7%), they were condition-specific. We divided these into two subgroups: Firstly, tests that are in themselves non-evidence based, and secondly, the condition tested for is non-evidence based to differentiate how non-evidence based tests might present different concerns: For Subgroup 4A, without a reliable evidence base to support protocols for diagnosis and treatment, unproven DTC tests can be used to justify treatments for medical conditions that are not clinically recognised,[36] which can lead to further harms. In using tests in Subgroup 4B, consumers may be led to believe that they have a condition that requires treatment when there is little evidence to support this. Tests in this subcategory can be used to promote conditions that are not recognised by the medical community and the on-sale of associated non-evidence-based treatments, also known as 'disease mongering'.[37] Most notably, these unproven tests are often high in cost, including the most expensive item found in the review, at AU$1947.

Table 5: Category 4 results

Inclusion Criteria	Target condition or test purpose	Key analyte or testing method	Mode of test#				Min. cost	Max. cost
			H	S	L	TN		
Subgroup 4A *(n= 70, 14.5% of total)*								

For peer review only - http://bmjopen.bmj.com/site/about/guidelines.xhtml

Current evidence does not support the clinical validity of the testing method for the condition it aims to test for.	Environmental toxin exposure	Hair metal & mineral analysis	0	4	0	**4**	175.00	325.00
	Environmental toxin exposure	Mycotoxin test	0	4	0	**4**	535.00	735.00
	Food allergy	Hair analysis	0	6	0	**6**	29.00	297.00
	Food allergy	IgA	2	0	0	**2**	380.00	
	Food allergy	IgG	0	10	12	**22**	162.00	550.00
	Food sensitivity & intolerance	ALCAT	0	13	0	**13**	325.00	1,947.00
	Hair loss	Hormones	0	0	2	**2**	225.00	225.00
	Heavy metal exposure	Urine chelation	0	5	0	**5**	225.00	485.00
	Histamine intolerance	Whole blood histamine	0	0	3	**3**	69.00	325.00
	Liver detoxification	Urine metabolites	0	1	0	**1**	225.00	
	Mental health nutrition (Pfeiffer test)	Zinc, Copper, Zinc-Copper ratio	0	0	2	**2**	338.00	531.00
	Cancer, Autism	Nagalese in blood	0	0	1	**1**	303.00	
	Mental health disorders	Neurotransmitters in urine	0	1	0	**1**	360.00	
	Small intestinal bacterial overgrowth (SIBO)	Hydrogen & methane breath test	0	2	2	**4**	220.00	380.00
**Subgroup 4B** *(n=11, 2.3% of total)*								
Current evidence does not support the condition tested for as being established and recognised by the general medical community.	Adrenal fatigue	DHEA & Cortisol	0	4	1	5	110.00	255.00
	Intestinal permeability (Leaky Gut Syndrome)	Lactulose & mannitol; stool analysis	0	4	0	4	123.00	622.80
	Paediatric autoimmune neuropsychiatric disorders syndrome (PANDAS)	Antibody	0	0	1	1	1,700.00	
	Mental health conditions/Pyrrole disorder	Hydroxyhaemopyrrolin-2-one (HPL) & neurotoxin	0	1	0	1	199.00	

#Mode of access: H=home self-tests, S= self-collected DAPT, L=Lab-collected DAPT, TN=total number).

3.2. Average cost

The average cost of each category is statistically significantly different (p value <0.001). There is a relationship between higher clinical utility and lower cost, and vice versa. Category 1 has the lowest average cost of $77.16, and smallest range in cost ($24.95 - $330). Category 2 has more expensive tests with a mean cost of $138.44 (range of $13 to $903).

For peer review only

For peer review only - http://bmjopen.bmj.com/site/about/guidelines.xhtml

Category 3 had an average cost of $206.08 (range of $18.95 – $1199) and Category 4 had the most expensive tests with an average cost of $363.11 (range of $13 to $1947).

3.3. Pre-and post-test consultation and quality assurance

Services such as pre- and post-test consultation are crucial to optimising appropriate test selection and utilisation. Supplemental Figure 2 shows more than half of all DTC tests (n=273, 56.4%) did not state whether any pre- or post-test counselling was offered. Only a quarter of the products (n=123, 25.4%) included a post-test consultation with a healthcare professional, which may include non-medically trained health practitioner.[1] Another 9.7% (n=47) offered a post-test GP telephone consultation to be purchased separately.

Supplemental Figure 3 shows that Category 1 tests, which were most likely to be clinically useful, were least likely to offer pre- and post-test consultation. Supplemental Figure 4 shows a breakdown of quality assurance information by test mode. More than half of home self-tests and self-collected DAPT did not report quality assurance information in the online advertisement. While all lab-collected DAPT were designated for analysis in a NATA-accredited laboratory, there was no detailed information on accreditation status. A very small number of self -collected DAPT were designated for analysis in an overseas laboratory.

<insert Supplemental Figure 2, Supplemental Figure 3, Supplemental Figure 4>

4. Discussion

---

[1] Healthcare professional in Australia is defined as: (a) a medical practitioner, a dentist or any other kind of health care worker registered under a law of a State or Territory; or (b) a biomedical engineer, chiropractor, optometrist, orthodontist, osteopath, pharmacist, physiotherapist, podiatrist, prosthetist or rehabilitation engineer. Naturopaths and Nutritionists are only Health Professionals, with regard to the definition above, if they are registered under State or Territory law.

For peer review only - http://bmjopen.bmj.com/site/about/guidelines.xhtml

The wide range and large volume of DTC tests sold online in Australia suggest a sizable and lucrative market, targeting large numbers of consumers, both with and without symptoms. Accessed in a distinctly different way than for clinician-facilitated tests, there are certain DTC tests with attributes that will provide greater consumer benefits compared to those obtained in mainstream healthcare. The greater availability of DTC tests for stigmatised and under-tested conditions, such as STIs can potentially complement mainstream healthcare by addressing issues of medical underuse.[38] However, only a small proportion of all DTC tests found in this study were of a type likely to provide a clear benefit (Category 1, 10.7%). Meanwhile, the majority of tests found in this study would seldom be offered in mainstream healthcare unless there were very clear clinical justifications, if at all. These included tests that were recommended against by professional organisations due to concerns of medical overuse, and an array of non-evidenced tests not recognised by the medical community, as well as tests designed for medical management but rebranded as 'health checks' for asymptomatic people. These types of tests with potential for medical overuse, as identified in our categories of clinical utility, suggest significant implications for harms to consumers and the healthcare system.

By offering tests not normally obtainable in mainstream clinical care, DTC tests can appear to offer advantages in addition to ease and convenience of access. People's familiarity with undergoing a pathology test, which are often the same procedures ordered by doctors albeit under specific clinical circumstances, adds an element of scientific legitimacy to DTC testing.[36] Other products canvassed in the study depend on the hype and innovative appeal of emerging technologies such as nutrigenomic, microbiome and glycan tests, even though these are yet to demonstrate clinical utility in real-world settings.[39 40] In some cases DTC test results may be used to support diagnoses and treatments that are non-evidence based.[36] This may often target consumers on 'diagnostic odysseys' who are seeking answers for unexplained symptoms or unsatisfactory diagnosis in conventional medicine. For example, a number of tests in Category 4 were tests for gastrointestinal conditions, which are known to

For peer review only - http://bmjopen.bmj.com/site/about/guidelines.xhtml

be difficult to diagnose even in a clinical setting.[8] However, providing non-evidence-based diagnosis and treatments may further harm consumers already in a vulnerable position.

The largest target of DTC tests though are consumers who are healthy and asymptomatic, providing an untapped market for these products.[41] Over 40% of the tests found in this study aim to assess a range of normal, everyday functions such as sleep, ageing, physical activity, and hormone levels as potential indicators of pathological abnormality. The emerging wellness industry relies on the material data from these test results to support a pipeline of justifying and promoting the consumption of nutritional supplements and dietary advice.[42] [43] However, testing healthy populations without clear clinical justification will only normalise the notion that people are 'sick until proven healthy'.[14] The downstream consequence of this ‘low-value’ practice is the creation of unnecessary medical waste, and more opportunity for incidental and unnecessary diagnoses.

Another concern is the regulatory loophole that allows tests with greater implications for medical overuse to be offered as DAPTs, even though they cannot be supplied in the form of home self-tests in Australia. In a recent review of the *Therapeutic Goods (Excluded Purposes) Specification 2010*, tests for cancer and human genetics were considered too high risk and clinically complex to be used without professional guidance, and therefore cannot be supplied as DTC home self-tests.[1] Yet our study shows these tests were being sold as lab-collected or self-collected DAPT. They offer consumers an alternative channel of access to these ‘higher risk’ tests because they are processed in a laboratory rather than at home. The fact that most DAPT are processed at NATA accredited laboratories may reinforce their perceived trustworthiness. However, laboratories do not have the capacity to recognise whether the tests are the right ones for the right patient, nor can they influence how consumers and requesting DTC companies make clinical decisions based on test results. Furthermore, our findings suggest there is variation in the quality assurance and the provision

For peer review only - http://bmjopen.bmj.com/site/about/guidelines.xhtml

of pre- and post-test consultation in DAPTs, and thus compromising the safety and benefits for consumers.

While our findings conclude that the majority of DTC tests currently on the market are unlikely to benefit the everyday consumer, recognisably, some products may be clinically useful if professional consultation and balanced information is provided. However, our study shows that professional consultation is inconsistently provided, or incurs a separate cost to consumers. Currently, no policy, regulation or industry codes provide clear guidance on the quality and accessibility of this important aspect of testing.

There are some limitations to the study. The analysis by clinical utility is highly contextual and interpreted by the authors. We acknowledge the potential for the categories to overlap, and that there may be other approaches to analysis. The DTC tests included in this time-specific search may not be exhaustive, as product availability may change over time, and others may be advertised in different platforms or sold in pharmacies. DTC tests offered by private commercial clinics but which are not advertised as a directly-purchasable item online, as is often the case for genetic and fertility tests, were also not included. The likelihood is there are many more such tests being sold in the market. This is an overview of products available; data extraction did not include an in-depth qualitative assessment of the textual information in each advertisement. Further study of this aspect would provide insight into the quality and transparency of DTC test advertisement information.

4.1. Conclusion & recommendations

The growing number of DTC tests available for direct purchase online in Australia purport to empower consumers with choice and convenience of access. The categorisation of clinical utility developed in this study shows the majority of currently available products lack clear benefits to consumers. The large offering of tests seldom recommended in a clinical setting may represent an under-recognised driver of medical overuse and consumption of low-value

For peer review only - http://bmjopen.bmj.com/site/about/guidelines.xhtml

healthcare, particularly amongst healthy consumers. Unlike publicly funded pathology services and hospital data that are auditable, the harms triggered by DTC tests are not easily tracked and measured. This may mean it remains a relatively hidden problem, with consumers and the healthcare system bearing the brunt of the immediate and long-term costs.

At the policy and regulation level, higher standards of test quality and regulation are needed to support informed decision-making for consumers.[16] Under the new European IVD Regulation there are increasing requirements for IVD companies to provide data to the regulator not just on the quality and analytical performance of tests but also evidence on the clinical performance of the devices, as well as a need for post-market surveillance on the performance of the tests.[44] Similarly, in Australia, DTC tests, especially those in Category 4, should fall under the same scrutiny and regulation before they are allowed to enter the online market. Guidelines or position statements from NATA or the Royal College of Pathologists of Australasia for DTC tests - other than those already issued for genetic tests - would encourage best practice. Nevertheless, these professional organisation recommendations will be limited to the laboratory end of testing, rather than on consumer-facing marketing and promotion of DTC tests. At the industry level, a commitment to provide balanced information about the evidence-base and potential clinical utility for the right populations is needed; as is the assurance of pre- and post-testing consultation with appropriately qualified professionals. Consumers of varying levels of health literacy must be supported to critically engage and assess the applicability and suitability of different DTC tests. To ensure test results and patient safety are adequately followed up by medical professionals, the introduction of consumer-led reporting systems[38] such as digital registries of test outcomes similar to those used for COVID-19 RATs is recommended. Currently Choosing Wisely and NICE ‘Do Not Do’ guidelines are developed for clinical settings where clinicians and consumers are both involved; these could be adapted to support decision-making in the DTC context.

References

For peer review only - http://bmjopen.bmj.com/site/about/guidelines.xhtml

1. Therapeutic Goods Administration. *Summary and outcomes: review of the regulation of certain self-testing in vitro diagnostic medical devices (IVDs) in Australia*. 2020.
2. Orth M, Vollebregt E, Trenti T, Shih P, Tollanes M, Sandberg S. Direct-to-consumer laboratory testing (DTCT): challenges and implications for specialists in laboratory medicine. *Clinical Chemistry and Laboratory Medicine (CCLM)*. 2022;
3. Ayers AA. Understanding the benefits and risks of direct-to-consumer testing for urgent care. *Journal of Urgent Care Medicine*. 2021;
4. Ayala-Lopez N, Nichols JH. Benefits and Risks of Direct-to-Consumer Testing. *Archives of Pathology & Laboratory Medicine*. 2020;144(10):1193-1198.
5. Drummond S. What are the potential outcomes of allowing Australian consumers direct access to community pathology services?: A review of patient initiated testing. *Australian Journal of Medical Science*. 2016;37(1):14-18.
6. Lovett KM, Mackey TK, Liang BA. Evaluating the evidence: direct-to-consumer screening tests advertised online. *Journal of Medical Screening*. 2012;19(3):141-153.
7. Ryan A, Wilson S, Greenfield S, Clifford S, McManus R, Pattison H. Range of self-tests available to buy in the United Kingdom: an Internet survey. *Journal of Public Health*. 2006;28(4):370-374.
8. Wong A, White H, Plant A, Shipman A, Shipman K. Issues surrounding consumer-bought food-allergy testing. *Clinical and experimental dermatology*. 2022;47(3):547-552.
9. Galior KD, Baumann NA. Challenges with at-home and mail-in direct-to-consumer testing: preanalytical error, reporting results, and result interpretation. *Clinics in Laboratory Medicine*. 2020;40(1):25-36.
10. Ho A, Quick O. Leaving patients to their own devices? Smart technology, safety and therapeutic relationships. *BMC medical ethics*. 2018;19(1):1-6.
11. Geransar R, Einsiedel E. Evaluating online direct-to-consumer marketing of genetic tests: informed choices or buyers beware? *Genetic testing*. 2008;12(1):13-23.
12. Copp T, Nickel B, Lensen S, et al. Anti-Mullerian hormone (AMH) test information on Australian and New Zealand fertility clinic websites: a content analysis. *BMJ open*. 2021;11(7):e046927.
13. Carter SM, Rogers W, Heath I, Degeling C, Doust J, Barratt A. The challenge of overdiagnosis begins with its definition. *BMJ*. 2015;350:h869.
14. Welch HG, Schwartz L, Woloshin S. *Overdiagnosed: making people sick in the pursuit of health*. beacon press; 2012.
15. McAlister S, Barratt AL, Bell KJ, McGain F. The carbon footprint of pathology testing. *Medical journal of Australia*. 2020;212(8):377-382.
16. McCartney M, Watson J, Finney B, Salisbury C. Why blood testing companies need effective regulation. British Medical Journal Publishing Group; 2022.
17. Wilkinson E. The rise of direct-to-consumer testing: is the NHS paying the price? *bmj*. 2022;379
18. Bloss CS, Schork NJ, Topol EJ. Direct-to-consumer pharmacogenomic testing is associated with increased physician utilisation. *Journal of medical genetics*. 2014;51(2):83-89.
19. Giovanni MA, Fickie MR, Lehmann LS, et al. Health-care referrals from direct-to-consumer genetic testing. *Genetic Testing and Molecular Biomarkers*. 2010;14(6):817-819.
20. Elshaug AG, Watt AM, Mundy L, Willis CD. Over 150 potentially low-value health care practices: an Australian study. *Medical Journal of Australia*. 2012;197(10):556-560.
21. Brodersen J, Schwartz LM, Heneghan C, O'Sullivan JW, Aronson JK, Woloshin S. Overdiagnosis: what it is and what it isn't. Royal Society of Medicine; 2018. p. 1-3.
22. National Health and Medical Research Council. Direct-to-Consumer Genetic Testing: A Statement from the National Health and Medical Research Council (NHMRC). https://www.nhmrc.gov.au/sites/default/files/documents/reports/statement-consumer-genetic-testing.pdf

For peer review only - http://bmjopen.bmj.com/site/about/guidelines.xhtml

23. Tandy-Connor S, Guiltinan J, Krempely K, et al. False-positive results released by direct-to-consumer genetic tests highlight the importance of clinical confirmation testing for appropriate patient care. *Genetics in Medicine*. 2018;20(12):1515-1521.
24. Stanczyk FZ, Mandelbaum RS, Lobo RA. Potential pitfalls of reproductive direct-to-consumer testing. *F&S Reports*. 2022;3(1):3-7.
25. McGuire AL, Evans BJ, Caulfield T, Burke W. Regulating direct-to-consumer personal genome testing. *Science*. 2010;330(6001):181-182.
26. Chauvin A, Lang E, Bossuyt P. When less is more: mitigating the harms and risks of over-testing through shared decision making. *Canadian Journal of Emergency Medicine*. 2022;24(2):126-127.
27. Kennedy B, Thornburg R. Deduction, induction, and abduction. In: Flick U, ed. *Tthe SAGE Handbook of Qualitative Data Collection*. Sage; 2018:49-64.
28. OCEBM Levels of Evidence Working Group. *The Oxford Levels of Evidence 2*. 2009. https://www.cebm.ox.ac.uk/resources/levels-of-evidence/ocebm-levels-of-evidence
29. Groos A, Peardon-Freeman S, McFarlane K, et al. Free online chlamydia and gonorrhoea urine test request in Queensland, Australia: convenience of home sample collection versus pathology collection centre attendance for faster results. *Sexual Health*. 2021;18(3):254-259.
30. Kersaudy-Rahib D, Lydié N, Leroy C, et al. Chlamyweb Study II: a randomised controlled trial (RCT) of an online offer of home-based Chlamydia trachomatis sampling in France. *Sexually transmitted infections*. 2017;93(3):188-195.
31. Bhatia RS, Levinson W, Shortt S, et al. Measuring the effect of Choosing Wisely: an integrated framework to assess campaign impact on low-value care. *BMJ quality & safety*. 2015;24(8):523-531.
32. Cadamuro J, Ibarz M, Cornes M, et al. Managing inappropriate utilization of laboratory resources. *Diagnosis*. 2019;6(1):5-13.
33. Royal Australasian College of General Practitioners. *Guidelines for Preventive Activities in General Practice, 9th Edition*. 2016.
34. Lam JH, Pickles K, Stanaway FF, Bell KJ. Why clinicians overtest: development of a thematic framework. *BMC Health Services Research*. 2020;20(1):1-11.
35. Krogsbøll LT, Jørgensen KJ, Larsen CG, Gøtzsche PC. General health checks in adults for reducing morbidity and mortality from disease. *Cochrane Database of Systematic Reviews*. 2012;(10)
36. Jones SL, Campbell B, Hart T. Laboratory tests commonly used in complementary and alternative medicine: a review of the evidence. *Annals of Clinical Biochemistry*. 2019;56(3):310-325.
37. Moynihan R, Heath I, Henry D. Selling sickness: the pharmaceutical industry and disease mongering. *BMJ*. 2002;324(7342):886-891.
38. Exten C, Pinto CN, Gaynor AM, Meyerson B, Griner S, Van Der Pol B. Direct-to-Consumer STI Testing Services: A Position Statement from the American Sexually Transmitted Diseases Association. *Sexually transmitted diseases*. 2021;
39. Fiala C, Taher J, Diamandis EP. P4 medicine or O4 medicine? Hippocrates provides the answer. *The Journal of Applied Laboratory Medicine*. 2019;4(1):108-119.
40. Floris M, Cano A, Porru L, et al. Direct-to-consumer nutrigenetics testing: an overview. *Nutrients*. 2020;12(2):566.
41. Shih P, Prokopovich K, Degeling C, Street J, Carter SM. Direct-to-consumer detection of atrial fibrillation in a smartwatch electrocardiogram: Medical overuse, medicalisation and the experience of consumers. *Social Science & Medicine*. 2022:114954.
42. Flores M, Glusman G, Brogaard K, Price ND, Hood L. P4 medicine: how systems medicine will transform the healthcare sector and society. *Personalized medicine*. 2013;10(6):565-576.

For peer review only - http://bmjopen.bmj.com/site/about/guidelines.xhtml

43. Bjelakovic G, Nikolova D, Gluud LL, Simonetti RG, Gluud C. Antioxidant supplements for prevention of mortality in healthy participants and patients with various diseases. *Cochrane database of systematic reviews*. 2012;(3)
44. In vitro diagnostic medical devices, Regulation (EU) 2017/746 of the European Parliament and of the Council of 5 April 2017 on in vitro diagnostic medical devices and repealing Directive 98/79/EC and Commission Decision 2010/227/EU (2022).

For peer review only

For peer review only - http://bmjopen.bmj.com/site/about/guidelines.xhtml

Supplemental Figure 1. Levels of distinctions in analysis

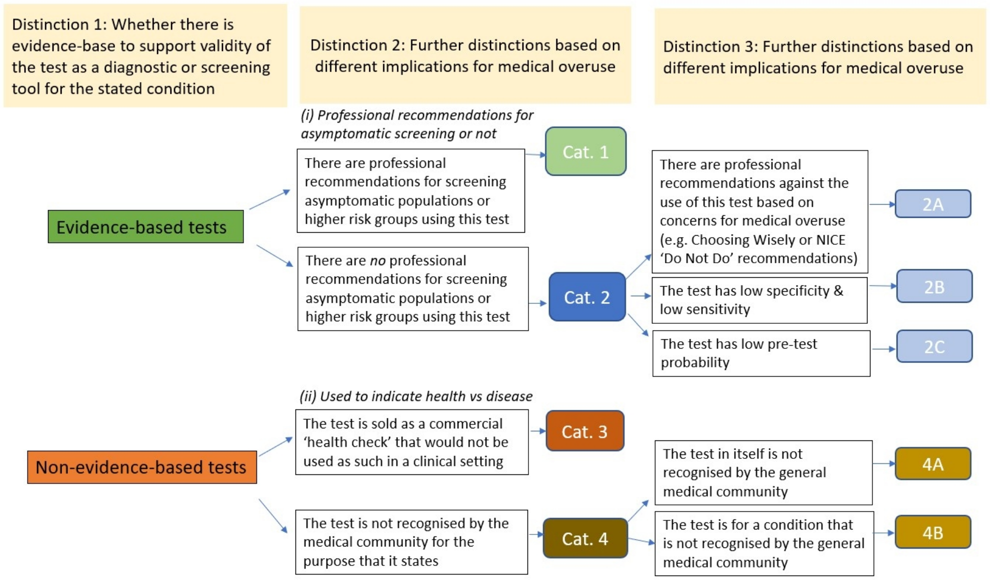

358x230mm (96 x 96 DPI)

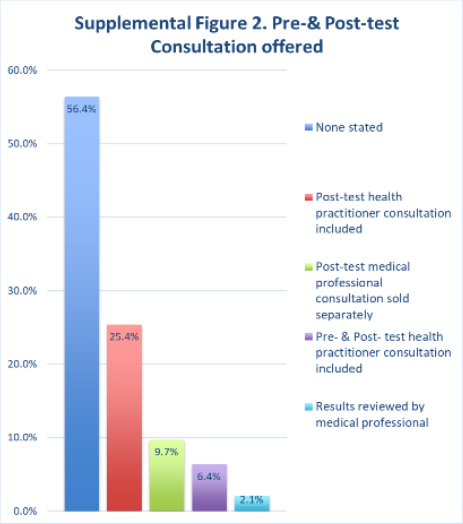

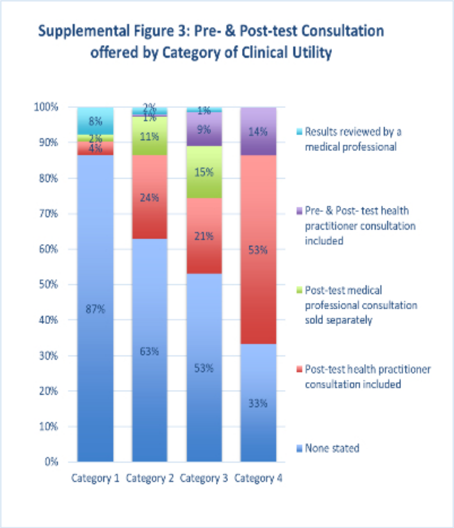

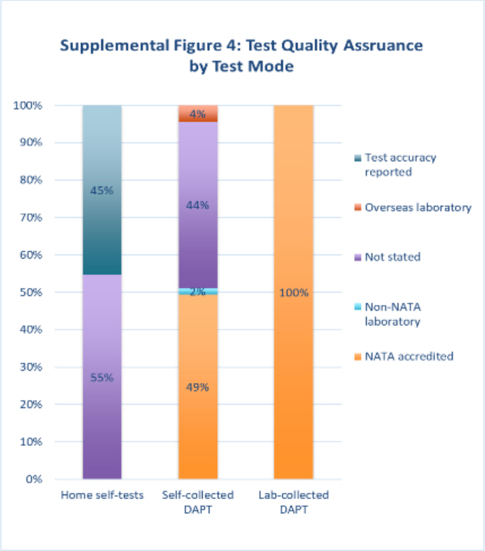

590x707mm (38 x 38 DPI)

Supplement 1

The following table presents the evidence used to support the inclusion of the tests into respective Categories and Subgroups, and the search and inclusion strategy used.

1. Category 1: Tests with potential clinical utility

Evidence to support inclusion

The test is recommended for asymptomatic screening of specific population groups by a recognised professional medical organisation, determined by a reported evidence-based medicine (EBM) approach.

Search & inclusion strategy

1. For each test, a Google Advanced search using key words: (“*medical condition or test purpose*, *analyte and/or testing method – see column 1*”) AND Screen* OR test* AND Recommend* OR Guide* AND Australia.
2. The first 30 records were screened for relevance. We prefaced Australian and Australasian professional organisations first, and then international professional organisations. The country or region of origin of the recommendations/guidelines is reported in Column 2.

Medical condition or test purpose; analyte and/or testing method	Reason for inclusion & Type of supporting evidence	Recommendation or summary of evidence	Reference
Bowel cancer (faecal occult blood test)	Recommended as an asymptomatic screening test for certain population groups; Government organisation (Australia)	Bowel and colorectal cancer screening is recommended for Australians aged 50 - 74 by the National Bowel Cancer Screening Program (every two years).	Australian Government, Department of Health and Aged Care (2022) National Bowel Cancer Screening Program, https://www.health.gov.au/initiatives-and-programs/national-bowel-cancer-screening-program. Accessed 19 June 2022.
Cardiovascular disease risk factors (Lipoproteins)	Recommended as an asymptomatic screening test for certain population groups; Recognised professional group (Australasia)	Adults should have their blood lipids assessed every five years starting at 45 years of age. Lipid levels should be interpreted in the context of an absolute CVD risk assessment after 45 years of age (35 years of age for Aboriginal and Torres Strait Islander peoples). Aboriginal and Torres Strait Islander adults should have lipid tests performed every five years from 35 years of age.	Royal Australasian College of General Practitioners (RACGP) (2021) Guidelines for preventive activities in general practice https://www.racgp.org.au/getattachment/1ad1a26f-9c8b-4e3c-b45b-3237272b3a04/Guidelines-for-preventive-activities-in-general-practice.aspx. Accessed 12 June 2022.
Chlamydia (Chlamydia trachomatis antigen)	Recommended as an asymptomatic screening test for certain population groups; Recognised professional groups (Australasia)	Chlamydia testing should be included in a standard STI check-up (gonorrhoea, chlamydia, HIV, syphilis) for asymptomatic sexually active populations. Routine screening is also recommended for men who have sex with men, young (heterosexual) Aboriginal and Torres Strait Islander people living in remote and very	RACGP (2021) Guidelines for preventive activities in general practice https://www.racgp.org.au/clinical-resources/clinical-guidelines/key-racgp-guidelines/view-all-racgp-guidelines/guidelines-for-

For peer review only - http://bmjopen.bmj.com/site/about/guidelines.xhtml

		remote areas, and travellers returning from high prevalence areas overseas.	preventive-activities-in-general-pr/communicable-diseases/sexually-transmissible-infections. Accessed 12 June 2022.<br>Australasian Society for HIV, Viral Hepatitis and Sexual Health Medicine (ASHM), Australian STI Management Guidelines (2022)<br>https://sti.guidelines.org.au/standard-asymptomatic-checkup/<br>. Accessed 12 June 2022.
Cholesterol level (Blood Cholesterol)	Recommended as an asymptomatic screening test for certain population groups; Recognised professional group (Australasia)	Adults 45 and over have their blood cholesterol level assessed every five years. Lipid levels should be interpreted in the context of an absolute CVD risk assessment after 45 years of age (35 years of age for Aboriginal and Torres Strait Islander peoples; Aboriginal and Torres Strait Islander adults should have lipid tests performed every five years from 35 years of age. For those with established CHD, or those at high risk, screening should be yearly.	RACGP (2022) Guidelines for prevention activities in general practice, Cholesterol and other lipids,<br>https://www.racgp.org.au/clinical-resources/clinical-guidelines/key-racgp-guidelines/view-all-racgp-guidelines/guidelines-for-preventive-activities-in-general-pr/prevention-of-vascular-and-metabolic-disease/cholesterol-and-other-lipids. Accessed 31 May 2022.
Diabetes (HbA1c, glucose)	Recommended as an asymptomatic screening test for certain population groups; Recognised professional group (Australasia)	Fasting glucose or glycated Hb is recommended every 3 years for people at high risk of Type 2 Diabetes (40+ and being overweight or obese, AUSDRISK score (questionnaire based risk calculator) of 12 or more. Consider screening for populations with increased risk, e.g. first-degree relative with diabetes, high-risk ethnicity, history of a previous cardiovascular event, women with history of gestational diabetes mellitus and polycystic ovary syndrome, patients on antipsychotic drugs); Those with impaired glucose tolerance test or impaired fasting glucose (not limited by age) should have fasting blood glucose tested every 12 months.	RACGP (2021) Guidelines for preventive activities in general practice<br>https://www.racgp.org.au/getattachment/1ad1a26f-9c8b-4e3c-b45b-3237272b3a04/Guidelines-for-preventive-activities-in-general-practice.aspx. Accessed 12 June 2022.
Gonorrhoea (Neisseria gonorrhoea antigen)	Recommended as an asymptomatic screening test for certain population groups; Recognised professional group (Australasia)	Gonorrhoea should be included in a standard STI check-up (gonorrhoea, chlamydia, HIV, syphilis) for asymptomatic sexually active populations. Routine screening is also recommended for men who have sex with men, young (heterosexual) Aboriginal and Torres Strait Islander people living in remote and very remote areas, and travellers returning from high prevalence areas overseas.	RACGP (2021) Guidelines for preventive activities in general practice<br>https://www.racgp.org.au/clinical-resources/clinical-guidelines/key-racgp-guidelines/view-all-racgp-guidelines/guidelines-for-preventive-activities-in-general-pr/communicable-diseases/sexually-transmissible-infections. Accessed 12 June

For peer review only

			2022.<br>Australasian Society for HIV, Viral Hepatitis and Sexual Health Medicine (ASHM), Australian STI Management Guidelines (2022)<br>https://sti.guidelines.org.au/standard-asymptomatic-checkup/. Accessed 12 June 2022.
Hepatitis B (Hepatitis B surface antigen)	Recommended as an asymptomatic screening test for certain population groups; Recognised professional group (Australasia)	Most people with chronic hepatitis B are asymptomatic unless their disease progresses (latent Hepatitis B). Opportunistic screening is recommended 1for high-risk groups (pp. 20) including people born in countries with a high prevalence, and Aboriginal and Torres Strait Island peoples.	ASHM, Testing Policy - Hepatitis B (2022) https://testingportal.ashm.org.au/files/ASHM_TestingPolicy_2020_HepatitisB_07_2.pdf. Accessed 12 June 2022.
Hepatitis C (Hepatitis C virus antibody)	Recommended as an asymptomatic screening test for certain population groups; Recognised professional group (Australasia)	Hepatitis C virus (HCV) testing should be performed as part of STI testing in people living with HIV, current HIV pre-exposure prophylaxis (PrEP) use, history of injecting drug use, anal sex with a partner with HCV infection, incarceration, non-professional tattoos or body piercings or receipt of organs or blood products before 1990. Asymptomatic screening is *not* recommended for other populations.	ASHM, Australian STI Management Guidelines (2022)<br>https://sti.guidelines.org.au/standard-asymptomatic-checkup/. Accessed 12 June 2022.
HIV (HIV-1 & HIV-2 antibody)	Recommended as an asymptomatic screening test for certain population groups; Recognised professional group (Australasia)	HIV should be included in a standard STI check-up (gonorrhoea, chlamydia, HIV, syphilis) for asymptomatic sexually active populations. Routine screening is also recommended for men who have sex with men, young (heterosexual) Aboriginal and Torres Strait Islander people living in remote and very remote areas, and travellers returning from high prevalence areas overseas.	RACGP (2021) Guidelines for preventive activities in general practice<br>https://www.racgp.org.au/clinical-resources/clinical-guidelines/key-racgp-guidelines/view-all-racgp-guidelines/guidelines-for-preventive-activities-in-general-pr/communicable-diseases/sexually-transmissible-infections. Accessed 12 June 2022.<br>Australasian Society for HIV, Viral Hepatitis and Sexual Health Medicine (ASHM), Australian STI Management Guidelines (2022)<br>https://sti.guidelines.org.au/standard-asymptomatic-checkup/. Accessed 12 June 2022.
Syphilis (Treponemal antibody)	Recommended as an asymptomatic screening test for certain population groups; Recognised professional group (Australasia)	Syphilis screening should be included in a standard STI check-up (gonorrhoea, chlamydia, HIV, syphilis) for asymptomatic sexually active populations. Routine screening is also recommended for men who have sex with men, young	RACGP (2021) Guidelines for preventive activities in general practice<br>https://www.racgp.org.au/clinical-resources/clinical-guidelines/key-racgp-

For peer review only - http://bmjopen.bmj.com/site/about/guidelines.xhtml

		(heterosexual) Aboriginal and Torres Strait Islander people living in remote and very remote areas, and travellers returning from high prevalence areas overseas.	guidelines/view-all-racgp-guidelines/guidelines-for-preventive-activities-in-general-pr/communicable-diseases/sexually-transmissible-infections. Accessed 12 June 2022.<br>Australasian Society for HIV, Viral Hepatitis and Sexual Health Medicine (ASHM), Australian STI Management Guidelines (2022)<br>https://sti.guidelines.org.au/standard-asymptomatic-checkup/. Accessed 12 June 2022.
Trichomoniasis infection (Trichomonas vaginalis lipophosphoglycan antibody)	Recommended as an asymptomatic screening test for certain population groups; Recognised professional group (Australasia)	Asymptomatic screening for trichomoniasis is only recommended in certain population groups and situations, e.g. in older people, people from regional and remote areas, especially Aboriginal and Torres Strait Islander people and street-based sex workers. Trichomoniasis is endemic in some Aboriginal and Torres Strait Islander populations in regional/remote areas and people should be tested according to local guidelines.	Australasian Society for HIV, Viral Hepatitis and Sexual Health Medicine (ASHM), Australian STI Management Guidelines (2022)<br>https://sti.guidelines.org.au/sexually-transmissible-infections/trichomoniasis/. Accessed 12 June 2022.

2. Category 2, Subgroup 2A: Tests with limited clinical utility - identified by experts as potentially contributing to medical overuse

Evidence to support inclusion

The test is identified as potentially contributing to medical overuse in certain populations by professionally recognised and EBM recommendations that aim to prevent medical overuse (e.g. Choosing Wisely, NICE 'Do Not Do' Recommendations, other recommendations against testing), as tests clinicians and consumers should question or avoid

Search & inclusion strategy:

1. Search terms for each test: "Do Not Do" OR "Choosing Wisely" AND recommend AND (*analyte and/or testing method - see column 1*) OR (*medical condition or test purpose - see column 1)*
2. Manual search of other known recommendations for and against screening (e.g. RACGP Guidelines for preventative activities in general practice)
3. The first 30 records were screened for relevance. We prefaced Australian and Australasian professional organisations first, and then international professional organisations. The country of origin of the recommendations/guidelines is reported in Column 2.

**Medical condition or test purpose; analyte and/or testing method**	**Reason for inclusion & Type of supporting evidence**	**Recommendation or summary of evidence**	**Reference**
Alzheimer's disease (Apolipoprotein E genotype)	Consumers advised to question the test by a Choosing Wisely Recommendation	Don't undertake genetic testing for methylenetetrahydrofolate reductase (MTHFR), apolipoprotein E (APOE) and other such tests where the clinical utility for	Choosing Wisely Recommendations, Human Genetics Society of Australasia (2022)

For peer review only - http://bmjopen.bmj.com/site/about/guidelines.xhtml

	(Australia)	diagnostic purposes is extremely low.	https://www.choosingwisely.org.au/recommendations/hgsa3. Accessed 23 May 2022.
Arthritis (Rheumatoid Factor)	Consumers advised to question the test by a Choosing Wisely Recommendation (United States (US))	Do not order rheumatoid factor (RF) alone, or as part of a 'panel' or 'cascade' in children to evaluate for rheumatologic disease such as juvenile idiopathic arthritis. due to musculoskeletal complaints. Do not let laboratory results guide referral.<br>The rheumatoid factor test is commonly used to help diagnose rheumatoid arthritis. However, a positive RF test does not always mean a person has rheumatoid arthritis, as there are several conditions that give positive rheumatoid factor results. Healthy people without rheumatoid arthritis can also test positive for rheumatoid factor, particularly older people. This does not mean they will develop the condition.	Choosing Wisely Recommendations, American Academy of Paediatrics (2022)<br>https://www.choosingwisely.org/clinician-lists/aap-sorh-rheumatoid-factor-to-evaluate-rheumatologic-disease/. Accessed 18 June 2022.<br>Arthritis Australia (2022)<br>https://arthritisaustralia.com.au/managing-arthritis/medical-management/blood-test-for-arthritis/. Accessed 18 June 2022.
Autoimmune disease (HLA-B27 antibody)	Consumers advised to question the test by a Choosing Wisely Recommendation (Canada)	Don't order an HLA-B27 unless spondyloarthritis is suspected based on specific signs or symptoms. HLA-B27 testing is not useful as a single diagnostic test in a patient with low back pain without further spondyloarthropathy, signs or symptoms because the diagnosis of spondyloarthropathy in these patients is of low probability. There is no clinical utility to ordering an HLA-B27 in the absence of positive imaging or the minimally required SpA signs or symptoms.	Choosing Wisely Recommendations, Canadian Rheumatology Association (2022) https://choosingwiselycanada.org/recommendation/rheumatology/. Accessed 18 June 2022.
Cancer markers (Carcino-embryonic antigen (CEA))	Consumers advised to question the test by a Choosing Wisely Recommendation (Australia)	Do not perform serum tumour marker tests except to evaluate or monitor a cancer known to produce these markers. In patients with non-specific symptoms, testing for a panel of tumour markers to try and diagnose an underlying cancer is not supported by evidence given the low sensitivity and specificity of these tests.	Choosing Wisely Recommendations, Medical Oncology Group of Australia (2022) https://www.choosingwisely.org.au/recommendations/moga4#. Accessed 23 May 2022.
Cancer risk factors (Glutathione S-transferases Genes test)	Consumers advised to question the test by a Choosing Wisely Recommendation (Australia)	Don't undertake genetic testing when clinical diagnostic criteria exist and there are no reproductive or predictive testing implications. Genetic tests do not have inherent utility. It is the adoption of therapeutic or preventive interventions that influences health outcomes.	Choosing Wisely Recommendations, Human Genetics Society of Australasia (2022) https://www.choosingwisely.org.au/recommendations/hgsa5. Accessed 25 August 2022.
Cardiovascular disease risk factors (Homocysteine test)	Consumers advised to question the test by a Choosing Wisely Recommendation (Australia)	Serum homocysteine as a screening test not recommended for low-risk populations. Its value as a risk factor for coronary heart disease is uncertain, and published RCTs show no evidence of benefit by lowering levels.	RCAGP Guidelines for preventative activities in general practice (2022) https://www.racgp.org.au/getattachment/1ad1a26f-9c8b-4e3c-b45b-3237272b3a04/Guidelines-for-preventive-activities-in-general-practice.aspx. Accessed 12 May

For peer review only - http://bmjopen.bmj.com/site/about/guidelines.xhtml

			2022.
Cardiovascular disease risk factors (MTHFR & APOE genetic test)	Consumers advised to question the test by a Choosing Wisely Recommendation (Australia)	Don't undertake genetic testing for methylenetetrahydrofolate reductase (MTHFR), apolipoprotein E (APOE) and other such tests where the clinical utility for diagnostic purposes is extremely low.	Choosing Wisely Recommendations, Human Genetics Society of Australasia (2022) https://www.choosingwisely.org.au/recommendations/hgsa3. Accessed 23 May 2022.
Coeliac disease (HLA gene typing)	Consumers advised to question the test by a Choosing Wisely Recommendation (Australia)	Do not undertake genetic testing for coeliac genes as a screening test for coeliac disease." The value of testing for coeliac genes is primarily as a negative test – if the gene test is negative then coeliac disease may be excluded. However as a coeliac gene can be found in approximately one third of the population, a positive result does not make coeliac disease a certainty. Serological testing, in a patient consuming an appropriate amount of gluten, is the appropriate first line screening test for coeliac disease. A small bowel biopsy is then required if serology is positive.	Choosing Wisely Recommendation, Gastroenterological Society of Australia (2022) https://www.choosingwisely.org.au/recommendations/gesa4#. Accessed 23 May 2022.
Food allergy (IgE)	Consumers advised to question the test by a Choosing Wisely Recommendation (Australia)	Food specific IgE testing should not be performed without a clinical history suggestive of IgE-mediated food allergy. While specific Immunoglobulin E (IgE) antibodies can be identified for a wide range of target allergens, the clinical significance of such tests is critically dependent on history. Since specific IgE antibodies are identifiable in many individuals with no evidence of allergic disease, the predictive value of these tests is strongly dependent on a history suggestive of underlying atopic disease with symptoms for which the detected IgE specificity is a plausible trigger.	Choosing Wisely Recommendation, Australasian Society of Clinical Immunology and Allergy (2022) https://www.choosingwisely.org.au/recommendations/ascia4. Accessed 12 May 2022. Australasian Society of Clinical Immunology and Allergy (2022) Laboratory Investigation of Allergic Diseases, https://www.allergy.org.au/hp/papers/tests-in-the-diagnosis-of-allergic-diseases. Accessed 12 May 2022.
Genital Herpes (antibody)	Consumers advised to question the test by a Choosing Wisely Recommendation (Australia)	Do not order herpes serology tests unless there is a clear clinical indication. Herpes serology is not an appropriate screening test in asymptomatic patients and does not accurately confirm whether the person is infected or is a transmission risk to others from asymptomatic shedding. Clinicians also need to consider whether test results will influence treatment or outcomes because, if they do not, then testing is a waste of finite health resources and is not indicated. Herpes serology tests only have good sensitivity and specificity in high prevalence populations. However, selective use of herpes serological tests may be justified for particular groups, such as those at high risk for STIs and human immunodeficiency virus (HIV) infection who are motivated to reduce their sexual risk behaviour; HIV-infected	Choosing Wisely Recommendations, Australasian Chapter of Sexual Health Medicine (2022) https://www.choosingwisely.org.au/recommendations/achshm1. Accessed 25 August 2022

For peer review only - http://bmjopen.bmj.com/site/about/guidelines.xhtml

		patients; patients with sexual partners with genital herpes; and in cases where a woman appears to have a first episode of herpes simplex virus (HSV) during pregnancy.	
Haemochromatosis (HFE gene mutation)	Consumers are advised to question the test by a Choosing Wisely Recommendation (US)	Don't order HFE genetic testing for a patient without iron overload or a family history of HFE-associated hereditary hemochromatosis. The majority of hereditary hemochromatosis is due to inheritance of HFE gene mutations. HFE gene mutations are common among individuals of European ancestry; however, only a small proportion of individuals with these mutations develop clinical disease. Other genetic and non-genetic factors contribute to disease expression. HFE genotyping should only be performed among individuals with iron overload or a known family history of HFE-associated hereditary hemochromatosis.	Choosing Wisely Recommendations, American College of Medical Genetics (2022) https://www.choosingwisely.org/clinician-lists/american-college-medical-genetics-genomics-hfe-genetic-testing/. Accessed 1 June 2022.
Heavy metal exposure (Heavy metal in blood)	Consumers are advised to question the test by a Choosing Wisely Recommendation (US)	Don't order heavy metal screening tests to assess non-specific symptoms in the absence of excessive exposure to metals. Individuals are constantly exposed to metals in the environment and often have detectable levels without being poisoned. Indiscriminate testing leads to needless concern when a test returns outside of a 'normal' range. Diagnosis of any metal poisoning requires an appropriate exposure history and clinical findings consistent with poisoning by that metal. A patient should only undergo specific metal testing if there is concern for a specific poisoning based on history and physical examination findings.	Choosing Wisely Recommendations, American College of Medical Toxicology and The American Academy of Clinical Toxicology (2022) https://www.choosingwisely.org/clinician-lists/american-college-academy-medical-toxicology-heavy-metal-screening-tests/. Accessed 10 October 2022
Immune disorders (Immunogenetics)	Consumers advised to question the test by a Choosing Wisely Recommendation (Australia)	Don't undertake genetic testing when clinical diagnostic criteria exist and there are no reproductive or predictive testing implications. Genetic tests do not have inherent utility. It is the adoption of therapeutic or preventive interventions that influences health outcomes.	Choosing Wisely Recommendations, Human Genetics Society of Australasia (2022) https://www.choosingwisely.org.au/recommendations/hgsa5. Accessed 25 August 2022
Insulin resistance (fasting glucose, fasting Insulin)	Consumers advised to question the test by a Choosing Wisely Recommendation (Australia)	Do not measure insulin concentration in the fasting state or during an oral glucose tolerance test to assess insulin sensitivity. Measurement of insulin either in the fasting state or during an oral glucose tolerance test is not a clinically useful method (and may be costly because of the insulin assay) to estimate insulin sensitivity.	Choosing Wisely Recommendations, The Endocrine Society of Australia (2022) https://www.choosingwisely.org.au/recommendations/esa3. Accessed 25 August 2022
Iron deficiency (Iron studies)	The test is not recommended by	Iron studies or serum iron should not be requested to diagnose iron deficiency:	Royal Children's Hospital Melbourne (2022) Clinical

For peer review only

For peer review only - http://bmjopen.bmj.com/site/about/guidelines.xhtml

	professional practice guidelines (Australia)	Serum iron reflects recent iron intake and does not provide a measure of the iron stores; Serum ferritin is an acute phase reactant and a normal result does not exclude iron deficiency in the presence of coexisting infection, inflammation or liver disease.	Practice Guidelines – Iron deficiency https://www.rch.org.au/clinicalguide/guideline_index/Iron_deficiency/. Accessed 11 October 2022.
Low-grade Inflammation (high sensitivity C-reactive protein (hsCRP))	Professional guidelines recommend against screening for this condition in certain populations (Australasia)	hsCRP test is a screening test not recommended by the RACGP Guideline for preventative practice, as there is insufficient evidence to support the role of hsCRP in preventive screening of asymptomatic patients.	RACGP (2021) Guidelines for preventive activities in general practice https://www.racgp.org.au/clinical-resources/clinical-guidelines/key-racgp-guidelines/view-all-racgp-guidelines/guidelines-for-preventive-activities-in-general-pr/communicable-diseases/sexually-transmissible-infections. Accessed 12 May 2022.
Neuro genetic disorders (Neurogenetic test)	Consumers advised to question the test by a Choosing Wisely Recommendation (Australia)	Don't undertake genetic testing when clinical diagnostic criteria exist and there are no reproductive or predictive testing implications. Like other screening or diagnostic tests, genetic tests do not have inherent utility. It is the adoption of therapeutic or preventive interventions that influences health outcomes. If clinical diagnostic criteria already exist for the condition in question and there are no reproductive or other predictive testing implications as a result of definitively identifying a genetic cause for the condition, then this renders genetic testing unnecessary.	Choosing Wisely Recommendations, Human Genetics Society of Australasia https://www.choosingwisely.org.au/recommendations/hgsa5. Accessed 25 August 2022
Prostate cancer (Prostate Specific Antigen (PSA))	Professional guidelines recommend against screening for this condition in certain populations (Australasia)	Do not perform PSA testing for prostate cancer screening in men with no symptoms and whose life expectancy is less than 7 years. Screening of asymptomatic (low-risk) men for prostate cancer by PSA testing is not recommended because the benefits have not clearly been shown to outweigh the harms. Pre-test discussion to address the benefits and harms (from overdiagnosis and overtreatment) of prostate cancer screening is recommended.	RACGP Guidelines for Preventative Activities in General Practice (2022) https://www.racgp.org.au/clinical-resources/clinical-guidelines/key-racgp-guidelines/view-all-racgp-guidelines/guidelines-for-preventive-activities-in-general-pr. Accessed 12 May 2022.
Thyroid disease (Thyroid hormones)	Consumers advised to question the test by a Choosing Wisely Recommendation (Australia)	Do not test thyroid function as population screening for asymptomatic patients. Clear evidence on the benefits and harms of screening is unavailable, and recommended against population-based screening. In the absence of evidence that early treatment reduces symptoms, lipid levels, or the risk of cardiovascular disease in patients with mild thyroid dysfunction detected by screening,	Choosing Wisely Recommendations, RACGP https://www.choosingwisely.org.au/recommendations/racgp10. Accessed 21 May 2022. RACGP Guidelines for Preventative Activities in General Practice (2022) https://www.racgp.org.au/clini

For peer review only - http://bmjopen.bmj.com/site/about/guidelines.xhtml

		screening for thyroid disease in asymptomatic populations is not recommended. This screening recommendation does not apply to people with symptoms suggestive of thyroid disease.	cal-resources/clinical-guidelines/key-racgp-guidelines/view-all-racgp-guidelines/guidelines-for-preventive-activities-in-general-pr. Accessed 12 May 2022.
Urinary tract infection (urine culture & microscopy)	Consumers advised to question the test by a Choosing Wisely Recommendation (US)	Don't obtain a urine culture unless there are clear signs and symptoms that localise to the urinary tract.	American Family Physician Choosing Wisely Recommendations (2022) https://www.aafp.org/afp/recommendations/viewRecommendation.htm?recommendationId=96. Accessed 21 May 2022.
Vitamin B deficiency (Vitamin B12, B6, B9)	Consumers advised to question the test by a NICE 'Do Not Do' Recommendation (United Kingdom (UK))	Tests for vitamin B12 deficiency & folate (B6) levels should not be carried out unless a full blood count and mean cell volume show a macrocytosis. Vitamin testing should be limited to patients with high-risk for deficiency, e.g. malabsorption or malnutrition	NICE 'Do Not Do Recommendation' (2022) https://www.nice.org.uk/donotdo/tests-for-vitamin-b12-deficiency-should-not-be-carried-out-unless-a-full-blood-count-and-mean-cell-volume-show-a-macrocytosis & https://www.nice.org.uk/donotdo/tests-for-folate-levels-should-not-be-carried-out-unless-a-full-blood-count-and-mean-cell-volume-show-a-macrocytosis. Accessed 31 May 2022.
Vitamin D deficiency (Vitamin D level)	Consumers advised to question the test by a Choosing Wisely Recommendation (Australia)	Do not perform population-based screening for Vitamin D deficiency; Clinicians should be aware that current vitamin D assay methods, may give different results and should understand the limitations of the method used in the laboratory that most of their patients attend.	Choosing Wisely Recommendation, Royal College of Pathologists Australasia (2022) https://www.choosingwisely.org.au/recommendations/rcpa3. Accessed 31 May 2022.
Vitamin K deficiency (Vitamin K level)	Consumers advised to question the test by a Choosing Wisely Recommendation (US)	Vitamin K deficiency is very rare. Measurements of the level of vitamin K in the blood are rarely used to determine if a deficiency exists. A diagnosis of Vitamin K deficiency is typically made by observing the prothrombin time correction following administration of vitamin K, plus the presence of clinical risk factors for vitamin K deficiency.	Choosing Wisely Recommendation, American Family Physician (2022) https://www.aafp.org/afp/recommendations/viewRecommendation.htm?recommendationId=244. Accessed 12 May 2022.

3. Category 2 Subgroup 2B: Tests with limited clinical utility - method has limited accuracy

Evidence to support inclusion

There is credible evidence to suggest the test or testing method has limited accuracy when used on its own

Search & inclusion strategy

For peer review only - http://bmjopen.bmj.com/site/about/guidelines.xhtml

1. PubMed and Google Advanced: (*"analyte and/or test method – see column 1")* AND diagnos* AND accuracy OR sensitivity OR specificity.
2. The first 30 records in each database were screened for relevance. i) We first prefaced EBM guidelines issued by Australian, Australasian and international professional or governmental organisations. The country or region of origin of the recommendations/guidelines is reported in Column 2. ii) If none were included, we included the peer-reviewed publication with the highest level of evidence (using Oxford Centre for Evidence-Based Medicine (CEBM): Levels of Evidence Grading[i]) were included. The type of evidence and CEBM grade level used is reported in Column 2. iii) If none were included, we then prefaced web-based consumer information issued by recognised expert health information organisations). A second or third publication may be included if the first publication included is insufficient to support the inclusion criteria.

**Medical condition or test purpose; analyte and/or testing method**	**Reason for inclusion & Type of supporting evidence**	**Recommendation or summary of evidence**	**Reference**
Blood clotting (Prothrombin Time)	Test has limited sensitivity or specificity (Peer-reviewed literature, CEBM Grade Level 1 – Systematic review)	The prothrombin time (PT) test measures the time it takes for blood to form a clot. It is usually used for monitoring patients already under clinical care, e.g. using warfarin and other Vitamin K antagonists to monitor treatment and adjust doses, and for preoperative testing. PT is limited by biological variability, insensitivity to other bleeding disorders, variability in results due to differences in reagents and coagulation. Routine preoperative haemostasis testing with PT is not useful in asymptomatic patients with no known risk factors. High rate of false-positive and false-negative results may lead to inappropriate precautionary measures or false reassurance.	Levy, J. H., Szlam, F., Wolberg, A. S., & Winkler, A. (2014). Clinical use of the activated partial thromboplastin time and prothrombin time for screening: a review of the literature and current guidelines for testing. Clinics in laboratory medicine, 34(3), 453-477.
Chronic fatigue (Iron & Vitamin B12, thyroid hormones)	Test method has limited accuracy when used on its own (Consumer information issued by recognised expert organisation)	Iron and vitamin B12 and thyroid hormones are important biomarkers of chronic fatigue and may play a role in treatment, diagnosis requires comprehensive assessment. Abnormal iron, Vitamin B12 and thyroid hormone levels may be an indicator of a range of other conditions, such as anaemia and thyroid disease.	Health Direct (2022) Chronic fatigue syndrome, https://www.healthdirect.gov.au/chronic-fatigue-syndrome-cfs. Accessed 12 May 2022
Coeliac disease (IgA, IgG antibody)	Test method has limited accuracy when used on its own (Peer-reviewed literature, CEBM Grade Level 5 – Expert opinion based on 'first principles')	Coeliac disease is an immune reaction to eating gluten, and creates inflammation that damages the small intestine's lining. Blood tests that check for antibodies (IgA & IgG) is typically the first step in diagnosing celiac disease, however, serological investigation alone is insufficient to make the diagnosis. A definitive diagnosis of coeliac disease	Lewis, D., Haridy, J., & Newnham, E. D. (2017). Testing for coeliac disease. *Australian prescriber*, *40*(3), 105

For peer review only - http://bmjopen.bmj.com/site/about/guidelines.xhtml

		requires gastroscopy and duodenal biopsy. Up to 5% of patients with coeliac disease can have negative serology.	
Gastrointestinal candida (IgG, IgM antibody)	Test method has limited accuracy when used on its own (Peer-reviewed literature, CEBM Grade Level 2 – level 2 evidence synthesis)	The candida fungus is part of the normal gastrointestinal track of healthy populations, and its presence is not sufficient to produce clinically significant candidiasis infection. The standard diagnostic test for gastrointestinal candidiasis is a blood culture. Antibody tests are nonculture diagnostic tests are non-invasive and have the potential to identify patients with currently unrecognised candidiasis and shorten the time to diagnosis. However, no nonculture *Candida* diagnostic tests have been shown to reduce mortality and morbidity, shorten hospital stays, or restrain the emergence of antifungal resistance.	Clancy, C. J., & Nguyen, M. H. (2018). Diagnosing invasive candidiasis. *Journal of Clinical Microbiology*, *56*(5), e01909-17.
Giardia (Rapid antigen test)	Test method has limited accuracy when used on its own (Peer-reviewed literature, CEBM Grade Level 3 – level 3 evidence synthesis)	Several immunodiagnostic tests of rapid diagnosis of giardiasis have been developed in decades, mainly based on the detection of Giardia antigens in faecal specimens. While many of these methods have high sensitivity, they should only serve as a complementary test to the gold standard traditional microscopy stool examination performed in the routine medical laboratory.	Hooshyar, H., Rostamkhani, P., Arbabi, M., & Delavari, M. (2019). Giardia lamblia infection: review of current diagnostic strategies. *Gastroenterology and hepatology from bed to bench*, *12*(1), 3–12.
Helicobacter pylori (Stool antigen)	Test method has limited accuracy when used on its own (Peer-reviewed literature, CEBM Grade Level 1 - Systematic review)	In people with no history of gastrectomy and those who have not recently had antibiotics or proton pump inhibitors, urea breath tests had high diagnostic accuracy while serology and stool antigen tests had lower accuracy to detect H pylori infection (Best et al, 2018).	Best, L. M., Takwoingi, Y., Siddique, S., Selladurai, A., Gandhi, A., Low, B., ... & Gurusamy, K. S. (2018). Non-invasive diagnostic tests for Helicobacter pylori infection. *Cochrane Database of Systematic Reviews*, (3).
Inflammatory bowel disease (Faecal calprotectin test)	The test should only be used based on assessing symptoms and medical history (Consumer information issued by recognised expert organisation and government organisation)	The faecal calprotectin test is used to distinguish between Inflammatory bowel disease (IBD) and irritable bowel syndrome (IBS), which is a functional condition of altered bowel habits with no inflammation. As a screening tool, faecal calprotectin tests reduce the need for invasive diagnostic procedures (such as colonoscopy), potentially leading to earlier diagnosis of IBD and IBS and subsequently improving patient management. However, these tests should only be limited to those necessary, based on symptoms and medical history.	Australian Government, Medical Services Advisory Committee (2022) Medical Measurement of Calprotectin as a marker of bowel inflammation http://www.msac.gov.au/internet/msac/publishing.nsf/Content/1353-public. Accessed 13 October 2022
Kidney disease (Creatinine	Test method has limited accuracy when used on its	Urine dipstick is an inexpensive, non-invasive and accessible test for	White, S. L., Yu, R., Craig, J. C., Polkinghorne, K. R., Atkins, R.

For peer review only - http://bmjopen.bmj.com/site/about/guidelines.xhtml

Albumin Ratio)	own (Peer-reviewed literature, CEBM Grade Level 3 – Cross sectional cohort study)	measuring albumin concentration in urine for the purpose of detecting kidney disease. However, this method has high false-positive rates, emphasising the need for laboratory confirmation of positive results. Laboratory Creatinine/Albumin Ratio is a recommended screening test for kidney disease, and is useful in managing diabetes and assessing the risk and progression of diabetic nephropathy. However, the tests should only be limited to those necessary, based on symptoms and medical history.	C., & Chadban, S. J. (2011). Diagnostic accuracy of urine dipsticks for detection of albuminuria in the general community. American Journal of Kidney Diseases, 58(1), 19-28.
Liver function (Bilirubin dipstick)	Test method has limited accuracy when used on its own (Peer-reviewed literature, CEBM Grade Level 4 - Retrospective review)	A urine dipstick measuring urine-bilirubin is a common and non-invasive measure of bilirubin, however, it is known to yield a high rate of false-positive results. In patients with an unexpected positive urine bilirubin test result, 85% had abnormal liver function test results after their positive urine bilirubin result. However, unexpected positive results amounted to only 0.13% of all test results. Urine bilirubin does not appear to add significant information toward the diagnosis of liver disease in most patients.	Foley, K. F., & Wasserman, J. (2014). Are unexpected positive dipstick urine bilirubin results clinically significant? A retrospective review. *Laboratory Medicine*, *45*(1), 59-61.
Measles, mumps, rubella serology (Antibodies)	Test method has limited accuracy when used on its own (Guideline issued by recognised professional organisation)	Routine serological testing for varicella does not provide a reliable measure of vaccine-induced immunity. Although the test can indicate whether natural immunity has occurred due to prior infection, it does not reliably guide diagnosis of clinically significant disease.	RACGP Guidelines for Preventative Activities in General Practice (2022) https://www.racgp.org.au/clinical-resources/clinical-guidelines/key-racgp-guidelines/view-all-racgp-guidelines/guidelines-for-preventive-activities-in-general-pr . Accessed 13 October 2022
Pancreatic insufficiency (pancreatic elastase 1)	Test method has limited accuracy when used on its own (Guideline issued by recognised professional organisation)	Pancreatic insufficiency is the inability of the pancreas to produce and/or transport enough digestive enzymes to break down food in the intestine and allow its absorption. The pancreatic elastase 1: test on its own may lead to false positives or misattribution of diagnosis.	Nikfarjam, M., Wilson, J. S., & Smith, R. C. (2017). Diagnosis and management of pancreatic exocrine insufficiency: Australasian Pancreatic Club Pancreatic Enzyme Replacement Therapy Guidelines Working Group. *Medical Journal of Australia*, *207*(4), 161-165.
Pancreatitis (lipase, amylase)	Test method has limited accuracy when used on its own (Peer-reviewed literature, CEBM Grade Level 3 – level 3 evidence synthesis)	Blood test for amylase and lipase rise within hours of an episode of acute pancreatitis are key components of the diagnostic criteria of acute pancreatitis. However, radiological findings are needed to confirm a diagnosis. Other conditions can also cause slightly	Basnayake C, Ratnam D. (2015) Blood tests for acute pancreatitis. Aust Prescr 2015;38:128-30.https://doi.org/10.18773/austprescr.043

For peer review only - http://bmjopen.bmj.com/site/about/guidelines.xhtml

		increased lipase levels. Using the test on its own may lead to false positives or misattribution of diagnosis.	
Urinary tract infection (Nitrite and leukocyte esterase dipstick)	The test method is not reliable on its own as a diagnostic test (Peer-reviewed literature, CEBM Grade Level 5 – Expert opinion based on 'first principles')	Urinary tract infections (UTI) or cystitis is a common bacterial infection. Nitrite and leukocyte esterase dipsticks are useful for ruling out and ruling in cystitis, but a positive urine culture with pyuria confirms the diagnosis.	Colgan R, Williams M. (2011) Diagnosis and treatment of acute uncomplicated cystitis. American Family Physician. 84(7):771-6.
Vaginal infection (pH)	The test has limited sensitivity or specificity (Peer-reviewed literature, CEBM Grade Level 3 – Cross sectional cohort study)	Vaginal bacterial infection is common among women of reproductive age. Vaginal pH determination with a swab is relatively sensitive, but less specific in detecting women with bacterial infection. The pH test may be suitable for home screening and direct patients towards the use of over-the-counter (OTC) antifungal medication, however, complicated vaginal infections should be diagnosed by healthcare providers.	Hemalatha, R., Ramalaxmi, B. A., Swetha, E., Balakrishna, N., & Mastromarino, P. (2013). Evaluation of vaginal pH for detection of bacterial vaginosis. *The Indian journal of medical research*, *138*(3), 354.

4. Category 2, Subgroup 2C: Tests with limited clinical utility – condition tested for has low pre-test probability

Evidence to support inclusion (2C)

There is credible evidence to suggests the condition tested for has low pre-test probability among the healthy general population.

Search & inclusion strategy

1. For each test: PubMed (with MeSH terms) and Google Advanced: (*"medical condition or test purpose" – see column 1)* OR (*"testing method and/or analyte" – see column 1*) AND Australia OR prevalence OR diagnos* OR test* OR screen*
2. The first 30 records in each database were screened for relevance. i) We first prefaced EBM guidelines issued by Australian, Australasian and international professional or governmental organisations. The country or region of origin of the recommendations/guidelines is reported in Column 2. ii) If none were included, we included the peer-reviewed publication with the highest level of evidence (using CEBM Grading) were included. The type of evidence and CEBM grade level used is reported in Column 2. iii) If none were included, we then prefaced web-based consumer information issued by recognised expert health information organisations).

Medical condition or test purpose; analyte and/or testing method	Reason for inclusion & Type of supporting evidence	Recommendation or summary of evidence	Reference
Cytomegalovirus (CMV) (IgC & IgM)	The test is for a condition with low pre-test probability in the general population (Consumer information issued by recognised health information organisation)	By the age of 20 years, around 50% of adults would have been infected with CMV, but it rarely causes symptoms. Most people with CMV infection may have a self-limiting flu-like or glandular fever-like illness. CMV tests are only needed to help diagnose a current,	MedlinePlus (2022) Cytomegalovirus (CMV) Tests, https://medlineplus.gov/lab-tests/cytomegalovirus-cmv-tests/ Accessed 31 May 2022

For peer review only - http://bmjopen.bmj.com/site/about/guidelines.xhtml

		reactivated, or past CMV infection in people at risk for health complications.	
Epstein-Barr virus (EBV) (EBV antibody)	The test is for a condition with low pre-test probability in the general population (Consumer information issued by recognised professional organisation & government organisation)	Approximately 95% of the world's adult population have been infected by EBV, but it rarely causes symptoms. An EBV antibody test may be needed in non-typical cases of mononucleosis or if another illness caused by EBV infection is suspected, or used to investigate flu-like symptoms in pregnancy.	Pathology Tests Explained (2022) Epstein Barr Virus Antibodies https://pathologytestsexplained.org.au/learning/test-index/epstein-barr-virus-antibodies. Accessed 21 May 2022<br>WebMD (2022) What to Know About the Epstein-Barr Virus Test https://www.webmd.com/a-to-z-guides/what-to-know-epstein-barr-virus-test. Accessed 21 May 2022
Glucose-6-Phosphate-Dehydrogenase deficiency (G6PD) (G6PD enzyme test)	The test is for a condition with low pre-test probability in the general population (Consumer information issued by recognised professional organisation, international)	G6PD deficiency is one of the most common forms of enzyme deficiency, affect more than 400 million people worldwide. However, the vast majority of people remain clinically asymptomatic throughout their lives. G6PD deficiency does not seem to affect life expectancy, quality of life, or the activity of affected individuals. Testing for G6PD deficiency should be considered when an acute haemolytic reaction triggered by exposure to a known oxidative drug, infection, or ingestion of fava beans; and prior to commencing treatment with certain medications such as primaquine, tafenoquine and rasburicase. It is not a routine test. Screening would only be justified for newborns in families with disease and in populations with higher risk for malaria.	Rare Diseases (2022) https://rarediseases.org/rare-diseases/glucose-6-phosphate-dehydrogenase-deficiency/. Accessed 21 October 2022
Lyme disease (Borrelia antibody)	The test is for a condition with low pre-test probability in the general population (Peer-reviewed literature, CEBM Grade Level 3 – level 3 evidence synthesis)	Lyme disease is caused by bacteria transmitted in a tick bite. The usefulness of the serological tests for Lyme disease depends on the pre-test probability and subsequent predictive values in the setting where the tests are being used. The types of tick that carry the bacteria are not native to Australia therefore it is unlikely to transmit Lyme disease. Treating false positive results, or treating patients with "Lyme disease-like illness" can lead to prolonged antibiotic therapy that is unjustified, and is likely to do much more harm than good.	Collignon, P. J., Lum, G. D., & Robson, J. M. (2016). Does Lyme disease exist in Australia? *Medical Journal of Australia*, *205*(9), 413-417.
Rotavirus (Rapid antigen test)	The test is for a condition with low pre-test probability in the general population (Consumer information	Rotavirus is an infectious disease most commonly among children under 5. The symptoms are diarrhoea, vomiting and stomach pains, and can cause	The Australian Government Department of Health (2022) Rotavirus Laboratory Case Definition (LCD)

For peer review only - http://bmjopen.bmj.com/site/about/guidelines.xhtml

	issued by government organisation)	severe gastroenteritis. There is no treatment for Rotavirus, but severe symptoms require hospitalisation. Commercially available rapid antigen detections kits are based on the VP6 antigen of group A rotaviruses, which means this is the only rotavirus serogroup detected. Recent experience in Australia has found a high false-positive rate for antigen detection tests compared with PCR, especially in vaccinated children. Confirmation of positive results should be done where there is a low pre-test probability that the person has rotavirus infection.	https://www1.health.gov.au/internet/main/publishing.nsf/Content/cda-phlncd-rotavirus.htm. Accessed 28/5/2022.

5. Category 3: Commercial 'health checks' with low potential clinical utility

Evidence to support inclusion

1. There is insufficient evidence to support clinical utility of the test as a routine assessment of health for low-risk and asymptomatic general practice populations; OR
2. There is literature to suggest the lack of clinical validity as a testing method for the named medical condition.

Search & inclusion strategy

1. For each test: a Google Advanced search using key words: (*"medical condition or test purpose - see column* 1) OR (*"analyte and/or testing method – see column 1"*) AND test* OR Recommend* OR Guide*. The first 30 records were screened for relevance. If no relevant sources were included, then PubMed (with MeSH terms): *(test or analyte – reported in column 1*) AND utility OR test* OR screen*
2. The first 30 records in each database were screened for relevance. i) We first prefaced EBM guidelines issued by Australian, Australasian and international professional or governmental organisations. The country or region of origin of the recommendations/guidelines is reported in Column 2. ii) If none were included, we included the peer-reviewed publication with the highest level of evidence (using CEBM Grading) were included. The type of evidence and CEBM grade level used is reported in Column 2. iii) If none were included, we then prefaced web-based consumer information issued by recognised expert health information organisations).

Medical condition or test purpose; analyte and/or testing method	Reason for inclusion & Type of supporting evidence	Recommendation or summary of evidence	Reference
Biological age (Glycans)	Insufficient evidence to support clinical utility of the test as an indicator of health status among healthy populations (Peer-reviewed literature, CEBM Grade Level 3 – level 3 evidence synthesis)	There is insufficient evidence to demonstrate clinical utility of measuring 'biological age', which is a hypothetical measure of the biological aging process, as opposed to chronological age as a marker of passage of time. There is still limited understanding about the mechanisms by which biological age predictors work. It remains unclear how best to	Jylhävä, J., Pedersen, N. L., & Hägg, S. (2017). Biological age predictors. EBioMedicine, 21, 29-36.<br>Nwanaji-Enwerem, J. C., & Mair, W. B. (2022). Redefining age-based screening and diagnostic guidelines: an opportunity for biological aging clocks in clinical medicine?. The Lancet Healthy

For peer review only - http://bmjopen.bmj.com/site/about/guidelines.xhtml

		incorporate measures of biological age into clinical medicine, and there is a need for trials that show modifying epigenetic age acceleration impacts human morbidity and mortality.	Longevity, 3(6), e376-e377.\
Bone health (serum alkaline phosphatase, calcium, potassium)	Insufficient evidence to support clinical utility of the test as an indicator of health status among healthy populations (Consumer information issued by recognised professional organisation, Australia)	An elevated level of bone markers may be seen in conditions such as osteoporosis or Paget disease. However, bone marker testing is typically only indicated in people who have been diagnosed with or are at risk of bone loss. The tests are not intended to be used to screen the general public.	Pathology Tests Explained (2022) Bone Markers, https://pathologytestsexplained.org.au/learning/test-index/bone-markers. Accessed 12 October 2022
Coenzyme Q10 (CoQ10) profile (Coenzyme Q10 level)	Insufficient evidence to support clinical utility of the test as an indicator of health status among healthy populations (Official information issued by government organisation, US)	CoQ10 has important functions in the body, people with some diseases may have reduced levels. However, low or high levels of CoQ10 do not necessarily indicate a problem in a person without symptoms of deficiency. Clinically significant coenzyme deficiency needs to be further diagnosed and not rely on blood plasma CoQ10 measures alone.	US Department of Health, National Centre for Complementary and Integrative Health (2022) Health Information – Coenzyme Q10 https://www.nccih.nih.gov/health/coenzyme-q10. Accessed 12 October 2022
Cytokine profile (Cytokine level)	Insufficient evidence to support clinical utility of the test as an indicator of health status among healthy populations (Guideline issued by recognised professional organisation, Australia)	Cytokines have been shown to regulate immunologic responses, hematopoietic development, and cell-to-cell communication as well as host responses to infectious agents and inflammatory stimuli. However, the clinical usefulness of measuring cytokine levels in plasma remains unclear. There is no definite diagnostic application for cytokine profile test at present.	RCPA Manual (2022) Cytokine Profile Test https://www.rcpa.edu.au/Manuals/RCPA-Manual/Pathology-Tests/C/Cytokine-profile-test. Accessed 6 October 2022
Fertility status - male (Semen analysis)	Insufficient evidence to support clinical utility of the test as an indicator of health status among healthy populations (Peer-reviewed literature, CEBM Grade Level 5 – Expert opinion based on 'first principles')	Semen analysis is subject to considerable interobserver variability and has been difficult to standardise, particularly with regard to the quantification of concentration and morphology. The results of semen analysis are often nuanced, making self-interpretation of results difficult without the guidance of a reproductive physician. Individual semen analysis parameters, even if abnormal, may be weak indicators of a man's fertility potential.	Stanczyk, F. Z., Mandelbaum, R. S., & Lobo, R. A. (2022). Potential pitfalls of reproductive direct-to-consumer testing. *F&S Reports*, *3*(1), 3-7.
Gastrointestinal function (Microbiome analysis)	Insufficient evidence to support clinical utility of the test as an indicator of health status among healthy populations (Guideline issued by recognised professional organisation,	The British Dieticians Association does not advise commercial gut microbiota testing and lifestyle change programs based on the test results. Research to date indicates there is no standard microbiome composition that can be used as a baseline for health and few	British Dieticians' Association, Commercial Gut Microbiome (2021) https://www.bda.uk.com/resource/commercial-gut-microbiome-testing.html. Accessed 11 July 2022.

For peer review only - http://bmjopen.bmj.com/site/about/guidelines.xhtml

	UK)	instances where there is a definitive association between any gut microbe and disease risk.	
Gastrointestinal function (Stool Analysis)	Insufficient evidence to support clinical utility of the test as an indicator of health status among healthy populations (Consumer information issued by recognised professional organisation, Australasia)	Laboratory analysis of stools can be useful , especially in gastroenterology. However, commercial stool analysis marketed as 'functional' assessment of gastrointestinal health may vary in protocol and the analysis, and do not have clinical utility for healthy populations.	Friends of Science In Medicine (FSM) Recommendations For Pathology Tests In Australia (2013) https://pathologytestsexplained.org.au/lto_au/media/images/FSM-pathologyrecommendations.pdf. Accessed 11 July 2022.
Health & Wellness profile (Panel consisting of tests for more than one of: full blood count, hormones, organ function, vitamins & minerals)	Insufficient evidence to support clinical utility of the test as an indicator of health status among healthy populations (Peer-reviewed literature, CEBM Grade Level 1 - Systematic review of level 1 studies)	General health checks are screening test for more than one disease or risk factor in more than one organ system in a person who does not feel ill. These aim to detect disease and risk factors for disease with the purpose of reducing morbidity and mortality. However, evidence does not suggest general health checks reduces morbidity or mortality, neither overall nor for cardiovascular or cancer causes, although the number of new diagnoses was increased. Important harmful outcomes, such as the number of follow-up diagnostic procedures or short-term psychological effects, were often not studied or reported and many trials had methodological problems.	Krogsbøll, L. T., Jørgensen, K. J., Larsen, C. G., & Gøtzsche, P. C. (2012). General health checks in adults for reducing morbidity and mortality from disease: Cochrane systematic review and meta-analysis. *BMJ*, *345*.
Hormone profile – female fertility status (Panel consisting of tests for more than one of: Follicle Stimulating Hormone, luteinising hormone, estradiol, progesterone, testosterone)	Insufficient evidence to support clinical utility of the test as an indicator of health status among healthy populations (Guideline issued by recognised professional organisation, Canada)	Tests for female reproductive hormone are used to investigate primary or secondary hypogonadism, infertility, prediction of ovulation, and for monitoring patients undergoing fertility treatment. The tests do not indicate the fertility status of healthy populations not under clinical care.	British Columbia Medical Association (2022) Hormone Testing – Indicators and Appropriate Use, https://www2.gov.bc.ca/gov/content/health/practitioner-professional-resources/bc-guidelines/special-endocrine-testing. Accessed 22 May 2022
Hormone profile – female health (Panel consisting of tests for more than one of: Follicle Stimulating Hormone, luteinising hormone, estradiol, progesterone, testosterone)	Insufficient evidence to support clinical utility of the test as an indicator of health status among healthy populations (Guideline issued by recognised professional organisation, Canada)	Tests for female reproductive hormone are used to investigate primary or secondary hypogonadism, infertility, prediction of ovulation, and for monitoring patients undergoing fertility treatment. The tests do not indicate the general health status of healthy populations not under clinical care.	British Columbia Medical Association (2022) Hormone Testing – Indicators and Appropriate Use, https://www2.gov.bc.ca/gov/content/health/practitioner-professional-resources/bc-guidelines/special-endocrine-testing. Accessed 22 May 2022

For peer review only - http://bmjopen.bmj.com/site/about/guidelines.xhtml

Hormone profile – general health (Panel consisting of tests for more than one of: adrenal, growth, thyroid & reproductive hormones)	Insufficient evidence to support clinical utility of the test as an indicator of health status among healthy populations (Guideline issued by recognised professional organisation, Canada)	Testosterone and luteinising hormone tests have indications for investigating primary or secondary hypogonadism. Sex hormone binding globulin (SHBG) is used to clarify borderline low total testosterone. Estradiaol, dehydroepiandrosterone (DHEA), androstenedione are not indicated in routine clinical practice. The tests do not indicate the general health status of healthy populations not under clinical care.	British Columbia Medical Association (2022) Hormone Testing – Indicators and Appropriate Use, https://www2.gov.bc.ca/gov/content/health/practitioner-professional-resources/bc-guidelines/special-endocrine-testing. Accessed 22 May 2022
Hormone profile – male health (Panel consisting of tests for more than one of: DHEA, estrone, estradiol, testosterone, SHBG, androstenedione)	Insufficient evidence to support clinical utility of the test as an indicator of health status among healthy populations (Guideline issued by recognised professional organisation, Canada)	Testosterone and luteinising hormone tests have indications for investigating primary or secondary hypogonadism. SHBG – clarification of borderline low total testosterone. Estradiaol, DHEA, androstenedione are not indicated in routine clinical practice. The tests do not indicate the general health status of healthy populations not under clinical care.	British Columbia Medical Association (2022) Hormone Testing – Indicators and Appropriate Use, https://www2.gov.bc.ca/gov/content/health/practitioner-professional-resources/bc-guidelines/special-endocrine-testing. Accessed 22 May 2022
Hormone profile – Menopause (Panel consisting of tests for more than one of: estradiol, estriol, estrone, DHEA)	Insufficient evidence to support clinical utility of the test as an indicator of health status among healthy populations (Consumer information issued by recognised professional organisation, US)	Testing hormone levels is not required to determine whether a woman has the "right amount" of hormones. The optimal hormone levels in postmenopausal women have not been established. How symptoms respond to a particular dose of hormones or non-hormonal menopause medication is the only reliable guide. Because hormone levels vary day to day as well as throughout the day, even a blood and saliva test cannot accurately reflect the body's hormone levels.	The North American Menopause Society (2022) What is hormone testing https://www.menopause.org/publications/clinical-practice-materials/bioidentical-hormone-therapy/what-is-hormone-testing-. Accessed 12 May 2022.
Hormone profile – ovarian reserve (Anti-Mullerian Hormone (AMH)	Insufficient evidence to support clinical utility of the test as an indicator of health status among healthy populations (Peer-reviewed literature, CEBM Grade Level 3 - cohort study)	AMH are used in the clinical setting for fertility treatments. In the general population and those not undergoing treatment for infertility, AMH does not predict future fertility potential, the likelihood of unassisted pregnancy, the time to pregnancy, or the timing of menopause.	Depmann M., Broer S.L., Eijkemans M.J.C., van Rooij I.A.J., Scheffer G.J., Heimensem J., et al. (2017) Anti-Müllerian hormone does not predict time to pregnancy: results of a prospective cohort study. Gynecol Endocrinol. ;33:644–648.
Hormone profile – sleep quality (Cortisol & Melatonin)	Insufficient evidence to support clinical utility of the test as an indicator of health status among healthy populations (Guideline issued by recognised professional organisation, Canada)	Cortisol (24-hour urine free) is used for targeted screening for Cushing Syndrome caused by endogenous cortisol excess only. Cortisol (serum/plasma) is not useful in the investigation of non-specific fatigue and lethargy unless accompanied by clinical syndromes of Addison's Disease or Cushing Syndrome. Measurements of morning and	British Columbia Medical Association (2022) Hormone Testing – Indicators and Appropriate Use, https://www2.gov.bc.ca/gov/content/health/practitioner-professional-resources/bc-guidelines/special-endocrine-testing. Accessed 22 May 2022

For peer review only - http://bmjopen.bmj.com/site/about/guidelines.xhtml

		afternoon cortisol (for diurnal variation) is not useful.	
Hormone profile – Sports & fitness profile (Growth & reproductive hormones)	Insufficient evidence to support clinical utility of the test as an indicator of health status among healthy populations (Guideline issued by recognised professional organisation, Canada)	Screening for growth hormone related disorders with insulin-like growth factor 1 (IGF1) is only indicated in patients demonstrating symptoms of growth hormone excess (acromegaly) or deficiency. The reference intervals are highly dependent on age. Tests for reproductive hormones are used to investigate primary or secondary hypogonadism and other endocrine disease or monitoring patients undergoing fertility treatment. Estradiaol, DHEA, androstenedione are not indicated in routine clinical practice. The tests do not indicate fitness or sports performance level in healthy populations.	British Columbia Medical Association (2022) Hormone Testing – Indicators and Appropriate Use, https://www2.gov.bc.ca/gov/content/health/practitioner-professional-resources/bc-guidelines/special-endocrine-testing. Accessed 22 May 2022.
Hormone profile – stress (salivary cortisol)	Insufficient evidence to support clinical utility of the test as an indicator of health status among healthy populations (Guideline issued by recognised professional organisation, Canada)	Salivary cortisol is used to investigate the cortisol response to stress in a research setting. In a clinical context, the utility of salivary hormone testing is limited to Cushing Syndrome screening using late night salivary cortisol.	Inder, W. J., Dimeski, G., & Russell, A. (2012). Measurement of salivary cortisol in 2012–laboratory techniques and clinical indications. *Clinical endocrinology*, *77*(5), 645-651.
Nutrigenomic profile (genetic)	Insufficient evidence to support clinical utility of the test as an indicator of health status among healthy populations (Guideline issued by recognised professional organisation, international)	The Consensus Report of the Academy of Nutrition and Dietetics suggest there is insufficient evidence regarding the effectiveness of incorporating nutrigenetic testing into nutrition counselling or care and reporting dietary or clinical outcomes at present. Research on the application of nutritional genomics to practice is in its infancy.	Braakhuis, A., Monnard, C. R., Ellis, A., & Rozga, M. (2021). Consensus report of the Academy of Nutrition and Dietetics: incorporating genetic testing into nutrition care. *Journal of the Academy of Nutrition and Dietetics*, *121*(3), 545-552.
Nutritional status (Amino acids)	Insufficient evidence to support clinical utility of the test as an indicator of health status among healthy populations (Guideline issued by recognised professional organisation, US.)	Assessing amino acid alone is not a reliable indication of nutritional status, because the reference range of amino acid concentrations vary with age. Interpretations of amino acid results are based upon relative amino acid levels, pattern recognition, and correlation of positive and negative findings, rather than on individual amino acids levels alone. Amino acid abnormalities or overall profiles should also be considered in the context of clinical findings and/or additional test results.	Sharer, J. D., De Biase, I., Matern, D., Young, S., Bennett, M. J., & Tolun, A. A. (2018). Laboratory analysis of amino acids, 2018 revision: a technical standard of the American College of Medical Genetics and Genomics (ACMG). *Genetics in Medicine*, *20*(12), 1499-1507.
Nutritional status (Ammonia)	Insufficient evidence to support clinical utility of the test as an indicator of health status among healthy populations (Peer-reviewed	In healthy populations, there is a normal range of ammonia in the blood but this will vary depending on the age of a person. A test for ammonia is not used as a routine screening test. It is	Tietze KJ. (2012) Review of Laboratory and Diagnostic Tests. In: Tietze KJ, ed. *Clinical Skills for Pharmacists (Third Edition).* Saint

For peer review only

	literature, CEBM level 2 – level 2 evidence synthesis)	used to diagnose and/or monitor conditions that cause high ammonia levels, such as liver disease, urea cycle disorders.	Louis: Mosby:86-122
Nutritional status (Chromium)	Insufficient evidence to support clinical utility of the test as an indicator of health status among healthy populations (Peer-reviewed literature,	Chromium deficiency has not been reported in healthy populations, and no definitive deficiency symptoms have been established. There is a lack of an accepted biomarker of chromium nutritional status.	Vincent JB, Lukaski HC. Chromium. (2018) Adv Nutr. Jul 1;9(4):505-506. doi: 10.1093/advances/nmx021. PMID: 30032219; PMCID: PMC6054252.
Nutritional status (Copper)	Insufficient evidence to support clinical utility of the test as an indicator of health status among healthy populations (Guideline issued by recognised professional organisation, Australasia)	Plasma copper is not a valid indicator of general health and nutritional status. The clinical purpose of a copper test is to diagnose Wilson's disease, Menkes disease and, occasionally, investigation of copper status in parenteral alimentation, and investigation of suspected copper toxicity.	RCPA Manual (2022) https://www.rcpa.edu.au/Manuals/RCPA-Manual/Pathology-Tests/C/Copper. Accessed 31 May 2022.
Nutritional Status (Essential Fatty Acids)	Insufficient evidence to support clinical utility as valid indicator of general health (Consumer information issued by recognised professional organisation)	Essential fatty acid testing or fatty acid profiling involves analysing specific types of fats in the blood stream. Commonly advertised are from fatty acids of the Omega-3 and Omega-6 groups.-Whilst there is good evidence that modifying diet to include more 'good' fats (i.e. omega-3) is beneficial, there is no evidence that testing fatty acids is able to provide any further information or health benefit.	LabTests Online UK (2022) https://labtestsonline.org.uk/tests/unvalidated-or-misleading-laboratory-tests. Accessed 31 May 2022
Nutritional status (Glutathione)	Insufficient evidence to support clinical utility as valid indicator of general health (Peer-reviewed literature, CEBM level 2 – level 2 evidence synthesis)	Measuring glutathione levels can help to determine if a person is deficient and may benefit from supplements or an increased diet of glutathione-rich foods. However, while optimizing glutathione levels has been proposed as a strategy for health promotion and disease prevention, clear, causal relationships between glutathione status and disease risk or treatment remain to be clarified.	Minich, D. M., & Brown, B. I. (2019) A review of dietary (phyto) nutrients for glutathione support. *Nutrients*, *11*(9), 2073.
Nutritional status (Iodine)	Insufficient evidence to prove clinical utility as valid indicator of general health (Peer-reviewed literature, CEBM level 3 – level 3 evidence synthesis)	There are limitations of assessing iodine status in the general population. Urine iodine is highly variable from day to day in a given patient and has low predictive value for iodine deficiency. Median urine iodine concentrations used in epidemiological studies should not be applied to individuals; if they are then iodine deficiency will be overdiagnosed. The need for multiple samples and a long sampling period severely limits the clinical utility of	Wainwright, P., & Cook, P. (2019). The assessment of iodine status– populations, individuals and limitations. *Annals of Clinical Biochemistry*, *56*(1), 7-14.

For peer review only - http://bmjopen.bmj.com/site/about/guidelines.xhtml

		such tests.	
Nutritional status (Magnesium test)	Insufficient evidence to prove clinical utility as valid indicator of general health (Peer-reviewed literature, CEBM level 3 – level 3 evidence synthesis)	There is no standardised laboratory test that accurately describes the status of magnesium. Due to the way in which magnesium is compartmentalised, typical blood and urine analytics may not provide an accurate proxy of magnesium status.	Workinger, Jayme L., Robert P. Doyle, and Jonathan Bortz. (2018) Challenges in the diagnosis of magnesium status. *Nutrients* 10, no. 9: 1202.
Nutritional status (Organic acids)	Insufficient evidence to prove clinical utility as valid indicator of general health (Guidelines issued a recognised professional organisation, Australia.	Nutritional status is not an indicator of an organic acid test. The normal range of organic acid levels in healthy asymptomatic individuals is varied. Further studies with well-defined patient groups exhibiting specific symptoms or diseases are warranted in order to discern between normal and pathological values.	RCPA Manual (2022) Organic acids https://www.rcpa.edu.au/Manuals/RCPA-Manual/Pathology-Tests/O/Organic-acids-urine. Accessed 31 May 2022.
Nutritional status (Selenium)	Insufficient evidence to prove clinical utility as valid indicator of general health (Guideline from a recognised professional organisation, Australia; consumer information from a recognised professional organisation, UK)	A selenium test is used for the detection of selenium deficiency in patients with dietary deficiency, especially patients on a non-supplemented synthetic amino acid diet, and the detection of selenium toxicity. Clinically significant selenium deficiency is manifest primarily as cardiomyopathy. This correlates poorly with plasma selenium levels and rarely occurs in selenium deficient patients on total parenteral nutrition. A nutrient test for selenium is useful but only in rare circumstances because clinically relevant deficiency is unlikely	RACP Manual (2022) Selenium https://www.rcpa.edu.au/Manuals/RCPA-Manual/Pathology-Tests/S/Selenium Accessed 11 October 2022. LabTests Online UK (2022) https://labtestsonline.org.uk/tests/unvalidated-or-misleading-laboratory-tests. Accessed 31 May 2022.
Nutritional status (Zinc)	Insufficient evidence to support clinical utility as valid indicator of general health (Guideline from professional and government organisation, Australia, New Zealand)	In a clinical setting, zinc plasma test is used to indicate zinc toxicity, or to assess people at risk of zinc deficiency (e.g. malnutrition, gastrointestinal disease). However, the zinc blood test is not very reliable for people with mild or low-level deficiency, because zinc is only present in small amounts in the body's cells.	RACP Manual (2022) Zinc https://www.rcpa.edu.au/Manuals/RCPA-Manual/Pathology-Tests/Z/Zinc. Accessed 11 October 2022. Auckland District Health Board (2022) Test Guide https://testguide.adhb.govt.nz/EGuide/. Accessed 11 October 2022.
Secretor status (Blood typing) (Antigens)	Insufficient evidence to prove clinical utility as valid indicator of general health (Peer-reviewed literature, CEBM level 1 - Systematic review)	Secretor status describes whether a person's blood type antigens (ABO blood type) are secreted in other body fluid such as saliva and mucus. Secretor status is determined by a FUT2 gene test. Secretor status test has been used to promote the 'Blood-Type Diet', based on the claim that each ABO blood type processes food differently, and adherence to a diet specific to an individual's ABO blood group could improve health, wellbeing, and energy levels and reduce risk of developing diseases such as cancer	Wang, J., García-Bailo, B., Nielsen, D. E., & El-Sohemy, A. (2014). ABO genotype,'blood-type'diet and cardiometabolic risk factors. *PloS one*, *9*(1), e84749.

For peer review only - http://bmjopen.bmj.com/site/about/guidelines.xhtml

		and cardiovascular disease. However, no evidence currently exists to validate the purported health benefits of blood type diets.	

6. Category 4, Subgroup 4A: Non-evidence-based tests with no potential clinical utility (Testing method is not recognised by the general medical community)

Description

The test method or condition tested for is not recognised by the general medical community. Current evidence does not support the validity of the test or the condition tested for. The results of non-evidence-based tests does not provide reliable clinical information to guide effective and beneficial management.

Evidence to support inclusion (4A):

There is insufficient evidence to support the clinical validity of the testing method for the condition it aims to test for.

Search & inclusion strategy

1. Google Advanced search terms: (*"test method and/or analyte" - see column 1)* AND diagnos* OR test* OR evidence*
2. PubMed and Google Advanced: (*"test method and/or analyte"- see column 1)* AND diagnos* OR test* OR evidence*

The first 30 records in each database were screened for relevance. i) We first prefaced EBM guidelines issued by Australian, Australasian and international professional or governmental organisations. The country or region of origin of the recommendations/guidelines is reported in Column 2. ii) If none were included, we included the peer-reviewed publication with the highest level of evidence (using CEBM Grading) were included. The type of evidence and CEBM grade level used is reported in Column 2. iii) If none were included, we then prefaced web-based consumer information issued by recognised expert health information organisations).

**Medical condition or test purpose; analyte and/or testing method**	**Reason for inclusion & Type of supporting evidence**	**Recommendation or summary of evidence**	**Reference**
Environmental toxins (Hair metal & mineral analysis)	Insufficient evidence to support the validity of the test method in the general medical community (Official information from government organisation, US)	A number of scientific issues need to be resolved before hair analysis can become a useful tool to understand environmental exposures. Although hair analysis may answer some questions about environmental exposure to certain substances, hair analysis often raises more questions than they answer. With very few exceptions, hair analysis results provide no insights as to whether an individual will develop health effects.	Agency for Toxic Substances and Disease Registry (2022) Analysis of Hair Samples: How Do Hair Sampling Results Relate to Environmental Exposures? https://www.atsdr.cdc.gov/HAC/hair_analysis/03-0330HairSampleTesting-Scientific.pdf. Accessed 10 October 2022.
Environmental toxins (Mycotoxin test)	Insufficient evidence to support the validity of the test method in the general medical community (Guideline issued by recognised intergovernmental organisation,	Mycotoxins are metabolites of some fungi that can cause illness primarily after ingestion of contaminated foods. Low levels of mycotoxins are found in many foods; therefore, mycotoxins are found in the urine of healthy persons. Mycotoxin levels that predict disease have not been established. Although	World Health Organizataion (2009) WHO guidelines for indoor air quality: Dampness and mould. http://www.who.int/indoorair/publications/7989289041683/en/. Accessed 10 October 2022.

For peer review only - http://bmjopen.bmj.com/site/about/guidelines.xhtml

	international)	mycotoxins can induce a wide range of adverse health effects in both animals and human beings, the evidence that they play a role in health problems related to indoor air is extremely weak	
Food allergy (Hair analysis, ALCAT, IgA, IgG)	Insufficient evidence to support the validity of the test method in the general medical community (Guideline issued by recognised professional organisation, Australasian)	Hair analysis, ALCAT, Immunoglobulin G (IgG) to foods are unproven, non evidence-based allergy 'tests' and 'treatments' that are provided by some unorthodox/alternative practitioners. There is currently no stringent government regulation of these methods. Consumers are strongly advised against the use of these tests.	Australasian Society of Clinical Immunology and Allergy (2021) Evidence-based vs Non-evidence based allergy tests and treatments https://www.allergy.org.au/images/pcc/ASCIA_PCC_Evidence-Based_vs_Non_Evidence-Based_Allergy_Tests-Treatments_FAQ_2021.pdf. Accessed 17 May 2022.
Hair loss (hormone tests)	Insufficient evidence to support the validity of the test method in the general medical community (Guideline issued by recognised professional organisation, Australia)	Hormone potentially contributes to hair health, but testing these analytes is not part of the diagnostic pathway for hair loss. Most cases of hair loss do not require pathology tests. Alopecia may be the result of a number of systemic or dermatological condition. Skin biopsy (from the active margin) may be helpful for lesions associated with inflammation or scarring.	RCPA Manual (2022) Alopecia https://www.rcpa.edu.au/Manuals/RCPA-Manual/Clinical-Problems/A/Alopecia. Accessed 17 May 2022.
Heavy metal chelation test (urine analysis)	Insufficient evidence to support the validity of the test method in the general medical community (Guideline issued by recognised professional organisation, US)	Post-chelator challenge urinary metal testing has not been scientifically validated, has no demonstrated benefit, and may be harmful when applied in the assessment and treatment of patients in whom there is concern for metal poisoning. The post-challenge urinary metal test and its application to assess of metal poisoning often leads to unwarranted and prolonged oral and/or intravenous administration of chelating agents. Chelation therapy based on such laboratory values, in addition to being of no benefit to patient outcome, may actually prove harmful.	American College of Medical Toxicology (2010). American College of Medical Toxicology position statement on post-chelator challenge urinary metal testing. *Journal of Medical Toxicology*, *6*(1), 74-75.
Histamine intolerance (diamine oxidase level)	Insufficient evidence to support the validity of the test method in the general medical community (Peer-reviewed literature, CEBM Grade Level 2 – level 2 evidence synthesis)	Histamine intolerance is a disorder associated with an impaired ability to metabolise ingested histamine, causing sensitive response to histamine in food. However, the symptoms are nonspecific an there is currently no consensus or validation of diagnostic tests for this condition. Currently available studies indicate an etiological relationship between diamine oxidase (DAO) deficiency and certain symptoms or disorders related to histamine intolerance. However, the evidence for the validity of blood DAO activity measurements for the diagnosis of histamine intolerance is neither	Comas-Basté, O., Sánchez-Pérez, S., Veciana-Nogués, M. T., Latorre-Moratalla, M., & Vidal-Carou, M. D. C. (2020). Histamine intolerance: The current state of the art. *Biomolecules*, *10*(8), 1181.

For peer review only - http://bmjopen.bmj.com/site/about/guidelines.xhtml

		abundant nor conclusive.	
Liver detoxification test (urine metabolites)	Insufficient evidence to support the validity of the test method among the general medical community (Guideline issued by recognised professional organisation, UK)	Liver detoxification tests, used mostly in complementary and alternative medicine (CAM), involves taking several common drugs e.g. paracetamol, aspirin and caffeine and measuring their metabolites in blood and urine afterwards. The amount and ratios of metabolites present are used as an indication of the amount of metabolism taking place in the liver. There is no evidence that the results of liver detoxification tests have any clinical utility in patients who do not have advanced liver disease. Genuine liver damage can be diagnosed using clinical examinations, imaging and other laboratory tests.	Lab Tests Online UK (2022) Unvalidated or misleading laboratory tests, https://labtestsonline.org.uk/tests/unvalidated-or-misleading-laboratory-tests. Accessed 21 May 2022
Mental health nutrition ('Pfeiffer Test')	Insufficient evidence to support the validity of the test method among the general medical community (Peer-reviewed literature, CEBM Grade Level 3 – level 3 evidence synthesis)	The Pfeiffer Test (to be distinguished from Pfeiffer disease) is a test used in CAM to analyse nutritional elements including zinc, copper and zinc-copper ratio in blood, promoted as an indicator of mental disorders such as attention deficit disorder (ADD), attention deficit hyperactivity disorder (ADHD), schizophrenia, Autism, learning disorders and mental disorders. While there is evidence to suggest that people with mental illness are likely to have lower or deficient levels of zinc or a copper-zinc imbalance, the causal link between low zinc and zinc-copper ratio imbalance to mental disorders is not established.	Robberecht, H., Verlaet, A. A., Breynaert, A., De Bruyne, T., & Hermans, N. (2020). Magnesium, iron, zinc, copper and selenium status in attention-deficit/hyperactivity disorder (ADHD). Molecules, 25(19), 4440.
Nagalase test	Insufficient evidence to support the validity of the test method among the general medical community (Guideline issued by recognised professional organisation, international & Australia)	Nagalase is an enzyme produced by cancerous cells. It is an indicator of early or potential cancer. A test for the Nagalase enzyme is most notably used as an indicator of a patients' response to a controversial cancer treatment using Gc Macrophage Activating Factor (GcMAF). There is no credible evidence to show that Gc-MAF can rebuild the immune system or eradicate early stage cancer. Studies that claim successful treatment using GcMAF have since been retracted. The Anticancer Fund and Cancer Council Australia have issued public warnings against using GcMAF as a cancer treatment	Cancer Council Australia (2022) Can GcMAF rebuild the immune system to eradicate early stage cancer? https://www.cancer.org.au/iheard/can-gcmaf-rebuild-the-immune-system-to-eradicate-early-stage-cancer. Accessed 17 May 2022<br>Ugarte, A., Bouche, G., & Meheus, L. [Anticancer Fund] (2014). Inconsistencies and questionable reliability of the publication "immunotherapy of metastatic colorectal cancer with vitamin D-binding protein-derived macrophages-activating, GcMAF" by Yamamoto et al. Cancer Immunology Immunotherapy, 63(12), 1347-8. doi: 10.1007/s00262-014-1587-y

For peer review only - http://bmjopen.bmj.com/site/about/guidelines.xhtml

Neurotransmitters urine test (Neurotransmitters in urine)	Insufficient evidence to support the validity of the test method among the general medical community (Peer-reviewed literature, CEBM Grade Level 2 – level 2 evidence synthesis)	Neurotransmitters excreted in the urine may have a place in clinical practice as a biomarker of nervous system function assess disturbances and monitor treatment efficacy for depression, ADHD, and inflammation. However, Urinary neurotransmitter assessment is yet to be accepted as diagnostic for depression or any particular disease or condition. There is variability of baseline measurements. The fraction of monoamines from the nervous system that appear in the urine, and under what conditions, is unclear.	Marc, D. T., Ailts, J. W., Campeau, D. C. A., Bull, M. J., & Olson, K. L. (2011). Neurotransmitters excreted in the urine as biomarkers of nervous system activity: validity and clinical applicability. *Neuroscience & Biobehavioral Reviews*, *35*(3), 635-644.
Small intestinal bacterial overgrowth (SIBO) - Hydrogen & methane breath test	Insufficient evidence to support the validity of the test method among the general medical community (Peer-reviewed literature, CEBM Grade Level 1 - Systematic review)	The lactulose hydrogen breath test is not a standard diagnostic test for SIBO. There is no validated breath test for the diagnosis of SIBO.	Khoshini, R., Dai, S. C., Lezcano, S., & Pimentel, M. (2008). A systematic review of diagnostic tests for small intestinal bacterial overgrowth. *Digestive diseases and sciences*, *53*(6), 1443-1454.

7. Category 4, Subgroup 4B: Non-evidence-based tests with no potential clinical utility (condition tested for is not recognised by the general medical community)

Evidence to support inclusion

There is insufficient evidence to support the condition as established and recognised by the general medical community

Search & inclusion strategy

1. Google Advanced search terms: (*condition - see column 1)* AND diagnos* OR test* OR evidence*

The first 30 records were screened for relevance.

2. PubMed and Google Advanced: (*condition - see column 1)* AND diagnos* OR test* OR evidence*

The first 30 records in each database were screened for relevance. i) We first prefaced EBM guidelines issued by Australian, Australasian and international professional or governmental organisations. The country or region of origin of the recommendations/guidelines is reported in Column 2. ii) If none were included, we included the peer-reviewed publication with the highest level of evidence (using CEBM Grading) were included. The type of evidence and CEBM grade level used is reported in Column 2. iii) If none were included, we then prefaced web-based consumer information issued by recognised expert health information organisations).

**Medical condition or test purpose; analyte and/or testing method**	**Reason for inclusion & Type of supporting evidence**	**Recommendation or summary of evidence**	**Reference**
Adrenal fatigue (DHEA & Cortisol)	Insufficient evidence to support the condition as established and recognised among the general medical community (Peer-reviewed literature, CEBM	The term 'adrenal fatigue' has been used by some doctors, healthcare providers, and the general media to describe an alleged condition caused by chronic exposure to stressful situations. Despite this, adrenal fatigue has not been recognised by any Endocrinology	Cadegiani, F. A., & Kater, C. E. (2016). Adrenal fatigue does not exist: a systematic review. *BMC endocrine disorders*, *16*(1), 1-16.

For peer review only - http://bmjopen.bmj.com/site/about/guidelines.xhtml

	Grade Level 1 - Systematic review)	society, as there is no sufficient evidence for its existence (Cadagiani & Kater, 2016). A systematic review by Cadagiani & Kater (2016) of 58 validated studies show that there is no substantiation that adrenal fatigue is an actual medical condition.	
Intestinal permeability (Lactulose & mannitol; stool analysis)	Insufficient evidence to support the condition as established and recognised among the general medical community (Peer-reviewed literature, CEBM Grade Level 3 – level 3 evidence synthesis)	Intestinal permeability (Leaky gut syndrome) describes a condition in which bacteria and toxins are able to pass through the intestinal wall into the bloodstream. Although barrier dysfunction has been described in a number of disorders, its role in their pathogenesis remains to be defined.	Quigley, E. M. (2016). Leaky gut–concept or clinical entity? *Current opinion in gastroenterology*, *32*(2), 74-79.
Paediatric autoimmune neuropsychiatric disorders (PANDAS) syndrome (Antibody test)	Insufficient evidence to support the condition as established and recognised among the general medical community (Peer-reviewed literature, CEBM Grade Level 2 – level 2 evidence synthesis)	Pediatric autoimmune neuropsychiatric disorders associated with streptococcal infections (PANDAS) is a hypothetical for a subset of children with rapid onset of obsessive-compulsive disorder (OCD) or tic disorders. Although there is a body of evidence that supports the existence of PANDAS and related conditions, it remains a controversial diagnosis. A number of studies investigated the PANDAS hypothesis do support the existence of these conditions. Further studies failed to identify significant differences in specific serum autoantibodies between PANDAS patients and healthy controls. Currently, none of the studied diagnostic approaches of PANDAS is sufficient to confirm the diagnosis.	Wilbur, C., Bitnun, A., Kronenberg, S., Laxer, R. M., Levy, D. M., Logan, W. J., ... & Yeh, E. A. (2019). PANDAS/PANS in childhood: Controversies and evidence. *Paediatrics & child health*, *24*(2), 85-91.
Pyrrole disorder (HPL & neurotoxin)	Insufficient evidence to support the condition as established and recognised among the general medical community (Peer-reviewed literature, CEBM Grade Level 1 - Systematic review)	Pyrrole disorder refers to a mood disorder attributed to elevated levels of pyrroles in the urine, also known as hydroxyhemepyrrolin-2-one (HPL). Hypothetically, the raised pyrrole levels lead to depletions in zinc and vitamin B_6, which, in turn, result in a range of psychiatric disorders, such as schizophrenia, anxiety, and depression.<br>However, elevated HPL is a clinically observed, but poorly researched biomarker with unclear associations with mental disorders.	Warren B, Sarris J, Mulder RT, Rucklidge JJ. Pyroluria: Fact or Fiction? J Altern Complement Med. 2021 May;27(5):407-415. doi: 10.1089/acm.2020.0151. Epub 2021 Apr 27. PMID: 33902305.

[i] Oxford Centre for Evidence-Based Medicine (CEBM): Levels of Evidence (2009) https://www.cebm.ox.ac.uk/resources/levels-of-evidence/oxford-centre-for-evidence-based-medicine-levels-of-evidence-march-2009 . Accessed 2 May 2022

For peer review only - http://bmjopen.bmj.com/site/about/guidelines.xhtml

BMJ Open

Direct-to-consumer tests advertised online in Australia and their implications for medical overuse: Systematic online review and a typology of clinical utility

Journal:	*BMJ Open*
Manuscript ID	bmjopen-2023-074205.R1
Article Type:	Original research
Date Submitted by the Author:	30-Jun-2023
Complete List of Authors:	Shih, Patti; University of Wollongong, School of Health & Society<br>Ding, Pauline; University of Wollongong, School of Mathematics and Applied Statistics<br>Carter, Stacy M; University of Wollongong,<br>Stanaway, Fiona; University of Sydney, School of Public Health<br>Horvath, Andrea ; NSW Health Pathology; University of New South Wales<br>Langguth, Daman; Wesley Hospital, Sullivan Nicolaides Pathology<br>Saad, Mirette; Australian Clinical Labs Victorian Central Laboratory Headquarters<br>St John, Andrew; Drajon Health, ; ARC Consulting,<br>Bell, Katy; The University of Sydney Faculty of Medicine and Health, School of Public Health; The University of Sydney
**Primary Subject Heading**:	Health services research
Secondary Subject Heading:	Health services research
Keywords:	HEALTH SERVICES ADMINISTRATION & MANAGEMENT, PATHOLOGY, Decision Making, Health Services Accessibility

SCHOLARONE™
Manuscripts

I, the Submitting Author has the right to grant and does grant on behalf of all authors of the Work (as defined in the below author licence), an exclusive licence and/or a non-exclusive licence for contributions from authors who are: i) UK Crown employees; ii) where BMJ has agreed a CC-BY licence shall apply, and/or iii) in accordance with the terms applicable for US Federal Government officers or employees acting as part of their official duties; on a worldwide, perpetual, irrevocable, royalty-free basis to BMJ Publishing Group Ltd ("BMJ") its licensees and where the relevant Journal is co-owned by BMJ to the co-owners of the Journal, to publish the Work in this journal and any other BMJ products and to exploit all rights, as set out in our licence.

The Submitting Author accepts and understands that any supply made under these terms is made by BMJ to the Submitting Author unless you are acting as an employee on behalf of your employer or a postgraduate student of an affiliated institution which is paying any applicable article publishing charge ("APC") for Open Access articles. Where the Submitting Author wishes to make the Work available on an Open Access basis (and intends to pay the relevant APC), the terms of reuse of such Open Access shall be governed by a Creative Commons licence – details of these licences and which Creative Commons licence will apply to this Work are set out in our licence referred to above.

Other than as permitted in any relevant BMJ Author's Self Archiving Policies, I confirm this Work has not been accepted for publication elsewhere, is not being considered for publication elsewhere and does not duplicate material already published. I confirm all authors consent to publication of this Work and authorise the granting of this licence.

For peer review only

Title: Direct-to-consumer tests advertised online in Australia and their implications for medical overuse: Systematic online review and a typology of clinical utility

Authors

Patti Shih, Australian Centre for Health Engagement Evidence & Values (ACHEEV), School of Health & Society, University of Wollongong, Wollongong, AUSTRALIA

Pauline Ding, School of Mathematics and Applied Statistics University of Wollongong, Wollongong, AUSTRALIA

Stacy M. Carter, Australian Centre for Health Engagement Evidence & Values (ACHEEV), School of Health & Society, University of Wollongong, Wollongong, AUSTRALIA

Fiona Stanaway, School of Public Health, University of Sydney, Sydney, AUSTRALIA

Andrea Rita Horvath, NSW Health Pathology, Sydney, AUSTRALIA & School of Biomedical Sciences, University of New South Wales, Sydney, AUSTRALIA

Andrew St John, Drajon Health, Toodyay, AUSTRALIA

Daman Langguth Wesley Hospital, Sullivan Nicolaides Pathology Brisbane, AUSTRALIA

Mirette Saad, Australian Clinical Labs, Melbourne, AUSTRALIA

Katy Bell, School of Public Health, University of Sydney, Sydney, AUSTRALIA

Corresponding author: Patti Shih, Building 29.319, School of Health & Society, University of Wollongong, Wollongong, Australia. pshih@uow.edu.au. +61 2 4221 4546.

Keywords: direct-to-consumer tests, self-testing, medical overuse, consumer health information, direct access pathology

Word count: 5053

For peer review only - http://bmjopen.bmj.com/site/about/guidelines.xhtml

Abstract

Objectives: To map the range and variety of direct-to-consumer (DTC) tests advertised online in Australia and analyse their potential clinical utility and implications for medical overuse.

Design: Systematic online search of DTC test products in Google and Google Shopping. DTC test advertisements data were collected, and analysed to develop a typology of potential clinical utility of the tests at population level, assessing their potential benefits and harms using available evidence, informed by concepts of medical overuse.

Results: We identified 484 DTC tests (103 unique products), ranging from AU$12.99 to AU$1947 in cost (mean $197.83; median $148.50). Using our typology, we assigned the tests into one of four categories: tests with potential clinical utility (10.7%); tests with limited clinical utility (30.6%); non-evidence-based commercial 'health checks' (41.9%); and tests whose methods and/or target conditions are not recognised by the general medical community (16.7%). Of the products identified, 56% did not state that they offered pre- or post-test consultation, and 51% did not report analytical performance of the test or laboratory accreditation.

Conclusions: This first-in-Australia study shows most DTC tests sold online have low potential clinical utility, with healthy consumers constituting the main target market. Harms may be caused by overdiagnosis, high rates of false positives, and treatment decisions led by non-evidence-based tests, as well as financial costs of unnecessary and inappropriate testing. Regulatory mechanisms should demand a higher standard of evidence of clinical utility and efficacy for DTC tests. Better transparency and reporting of health outcomes, and the development of decision-support resources for consumers are needed.

For peer review only

Strengths & limitations to this study

- This review strategy of DTC tests advertised online is comprehensive, and further analysed with a conceptual focus on the clinical utility of DTC tests. It provides a nuanced and critical angle on their implications for medical overuse and potential benefits and harms for consumers, and can be replicated and applied outside of Australia.
- The DTC tests included in this time-specific search may not be exhaustive, as product availability may change over time, and others may be advertised in different platforms or sold in pharmacies.
- DTC tests offered by private commercial clinics but which are not advertised as a directly-purchasable item online, as is often the case for genetic and fertility tests, were also not included.
- This is an overview of products available; data extraction did not include an in-depth qualitative assessment of the textual information in each advertisement.

For peer review only - http://bmjopen.bmj.com/site/about/guidelines.xhtml

1. Introduction

Direct-to-consumer (DTC) tests are commercially marketed pathology tests, purchased by members of the public,(1, 2) who pay for and decide on which test to access and when. DTC testing does not require the recommendation or referral from a consulting doctor, nor the facilitation of a public health program. This makes them distinct from home collection kits that are part of screening programs (e.g. bowel cancer screening), pandemic responses (e.g. COVID-19 rapid antigen tests (RATs)), point-of-care testing (as this is conducted within a clinical or care-based setting), or self-monitoring tests for patients already diagnosed with a condition or under clinical management (e.g. diabetes self-monitoring).

The size of the DTC test market is rapidly growing, expanding from a reported US$15.28 million per annum in 2010 to over US$352 million in 2020.(3) As part of a greater commercial shift towards more individualised and consumer-driven models of healthcare, DTC testing promises the opportunity for more proactive self-care and personal autonomy.(4, 5) No longer just reliant on screening programs or doctors' recommendations, consumers may (in theory) initiate and obtain pathology tests quickly and conveniently to inform early disease prevention or enable timely management decisions. Bypassing clinical visits may allow more privacy and confidentiality around testing, and may avoid the additional cost and logistics of travel and administration.(5)

Studies of DTC tests in the US and UK show a wide variety of diagnostic, screening and risk monitoring tests being available for direct purchase, suggesting consumers with and without symptoms are targeted.(6, 7) (8) However, only 15% of the DTC screening tests found in the US were supported by evidence-based recommendations, and in the UK, only one out of 24 companies offering allergy testing used approved methods from an accredited laboratory.(8) These findings suggest the need to scrutinise the *clinical utility* of DTC tests: whether the product leads to useful clinical decision-making and improved health outcomes. Clinical utility depends on getting the right test for the right person at the right time.(9) This

For peer review only - http://bmjopen.bmj.com/site/about/guidelines.xhtml

is determined by a variety of factors, such as an individual's medical history, symptoms (if any), and pre-test probability for the target condition. Other important considerations are the reliability and validity of the testing method, the quality of the in-vitro device, and the credibility of the laboratory. When products are directly-marketed, consumers may be making test decisions without the adequate opportunity for discussion and guidance from their doctors.(10) Indeed, several studies show that the quality and reliability of information on websites promoting tests varies,(6, 11, 12) which can expose consumers to the harms of choosing inappropriate and/or unnecessary tests.

An often under-analysed harm related to pathology tests of limited clinical utility is medical overuse: healthcare that is either harmful or does not lead to improved health outcomes.(13, 14) This includes using tests inappropriately or unnecessarily, such as testing low-risk and asymptomatic populations outside of clinical recommendations. This can drive the unnecessary diagnosis of early or slow-progressing diseases that would not have harmed the patient in their lifetime (overdiagnosis), and instead lead to treatments that produce no significant benefit but may cause harm (overtreatment).(13, 14) Testing, whether using home testing kits or at a laboratory, is resource intensive and produces medical waste.(15) Testing demands further clinical resources such as analysis and interpretation, and decision-making based on results, whether positive or negative.(16, 17) Test results can also trigger downstream testing and unjustified treatments that generate further costs and resource-use in the health system, such as unnecessary General Practitioner (GP) visits.(18, 19) When testing decisions are not clinically justified or do not lead to improvement in health outcomes, they are a form of 'low-value care', creating waste and additional burden to the healthcare system.(20) Although it is well-recognised that medical overuse occurs even in clinician-guided settings,(13, 21) it may be exacerbated in a DTC context where consumers may be making testing decisions with little support or balanced information.(17)

For peer review only - http://bmjopen.bmj.com/site/about/guidelines.xhtml

Considerable critical attention has already been paid to the questionable clinical utility of the growing DTC genetic testing and fertility treatment industry.(22-24) For example, genetic tests for conditions such as Alzheimer's disease may predict a person's risk of eventually developing the condition where prevention strategies are limited, and testing can also cause psychosocial distress to healthy people.(25) The results of the anti-Mullerian hormone ('Egg Timer') test measuring ovarian reserve are unreliable as a basis for making family planning decisions in the future, as the test is only potentially useful for patients undergoing a current fertility treatment.(12) Typically, these better-known DTC genetic and fertility tests are offered by private clinics that include a suite of services and consultations before tests are chosen and performed 'in-house'.

An emerging trend in DTC testing that has had limited exploration is the 'direct purchase' online testing market: pathology test products and services advertised widely on the internet, and immediately purchasable in a single online transaction. Broadly, there are three types of directly-purchased DTC tests: 1) *Home self-tests* - tests kits used by consumers themselves producing immediate results for their own interpretation; 2) *Self-collected direct accessed pathology tests (DAPTs)* - a pathology test requested from a commercial company on behalf of a consumer who purchased the test, rather than requested by a doctor after consultation and discussion, with samples collected at home and mailed into a laboratory; or 3) *Lab-collected DAPTs*, again purchased directly by a consumer, but with samples collected in a laboratory. Home self-tests and DAPTs signal different levels of involvement of laboratory professionals and have different regulatory requirements depending on the jurisdiction. For example, in Australia, home self-tests are regulated by the Therapeutic Goods Administration (TGA), whereas laboratory-tested DAPTs are regulated by the National Association of Testing Authorities (NATA]). The commonality is that consumers are the primary decision-makers in testing and purchasing decisions. While some DTC testing services do offer professional consultations as an inclusion or as an add-on purchase, if this happens at all, testing decisions are already made before there is an opportunity to discuss and

For peer review only - http://bmjopen.bmj.com/site/about/guidelines.xhtml

deliberate about the personal values and expectations of the consumer in relation to taking a specific test.(26)

Currently, there is limited research available on DTC tests available in Australia, nor has there been much assessment of the potential benefits and harms of the different products available. This first-in-Australia study reviews DTC tests advertised online, with a focus on their clinical utility and implications for medical overuse. The research questions for this study are: 1) How many and what variety of products are advertised and available to Australian consumers online? 2) What are their implications for medical overuse?

2. Methods

The study took place in two stages. Firstly, a systematic search of DTC test products advertised online was conducted in Australia; Secondly, the data collected was developed into a typology of potential clinical utility of the product, which was analysed based on the potential benefits and harms of the test at a population level, informed by concepts of medical overuse.

Stage 1 - Systematic online search: A pilot search was conducted in Google and Google Shopping by two researchers independently in October 2020. These engines were chosen because the majority of internet searches for product purchases in Australia are conducted on these platforms. The pilot search identified the three modes of DTC tests (home self-tests, self-collected DAPT and lab-collected DAPT) and a range of medical conditions and/or types of tests. This helped refine the search terms used in an updated search (Table 1), taking place between June and August 2021. We allowed snowball inclusion of new items between August - December 2021, whereby additional products that met the study inclusion criteria were added if they appeared on the same websites advertising already-included products. Google search engine algorithms display search results according to relevance ranking by numerical order: the first ranked results and pages will be the most relevant, with a decline in relevance of search results in subsequent pages. Despite the searches returning large number of total

For peer review only - http://bmjopen.bmj.com/site/about/guidelines.xhtml

results, only very small, negligible percentages of relevance are expected in later pages, particularly after 200 results.(27, 28) Therefore, we reviewed the first 20 pages (10 results per page, totalling 200 results) from each search. While it can never be guaranteed that this cut-off point will not miss relevant results, it is the most practically and logistically viable approach to conduct the review with a standardised cut-off point for each search scan. We excluded sites that were not relevant, and any duplicate results. We created a data extraction table to chart data relevant for the assessment of clinical utility and medical overuse, and collected these data from the website of each included product: name of product, condition and/or analyte tested for, specimen sampled, mode of the test, cost in Australian dollars (including shipping), whether the product offers any pre- or post-test consultations; and any indicators of the quality assurance of the tests (for home self-tests, this is indicated by information about the diagnostic accuracy of the test, expressed as clinical sensitivity and specificity metrics; and for DAPT, this is indicated by any information on the accreditation of nominated laboratories or individual tests).

Table 1: Data search and selection strategy

Search strategy	Description	
Search string 1	Home self-tests & Self-collected pathology tests	Search: "self test", "self diagnosis","home test", "home diagnosis", "home screen", "self screen",
Search string 2	Direct access pathology & Self-collected pathology tests	Search string 2: "direct access test*", "self request* pathology", "private blood test*", "private medical test", "online blood test".
Search inclusions & exclusions		The published region was set in Australia. The search excluded the term COVID, and .gov and.edu domains, to omit any screening tests that are offered by government screening programs.
Selection inclusion	To be included in the study:<br>For all tests:<br>i. The tests are intended to indicate the presence of disease or conditions (e.g. diagnose a disease), or for general well-being purposes (e.g. tests that purport to indicate level of hydration, vitamins, hormones)	

For peer review only - http://bmjopen.bmj.com/site/about/guidelines.xhtml

	ii. The product can be shipped to an Australian address<br>iii. A test must be a purchasable product, itemised and displayed in AUD that is able to be purchased via an online transaction<br>• Home self-tests: must include an in-vitro device that collects a human tissue sample (e.g. a lancet or container for blood, urine, saliva, faecal material); samples are processed at home (e.g. testing strip)<br>• DAPT self-collected: must include an in-vitro device that collects a human tissue sample (e.g. a lancet or container for blood, urine, saliva, faecal material); samples are sent to be processed at a laboratory<br>• DAPT lab-collected services: must be a pathology request for a named biomarker or analyte that is tested in a laboratory
Selection exclusion	The product is excluded if it is:<br>i. Not for testing human samples (e.g. animals, soil, water)<br>ii. Intended for use by professionals or clinics, e.g. doctors, hospitals or laboratories<br>iii. Used or purchased with a separate product (e.g. used with a meter or reader or as part of a health coaching program)<br>iv. Funded as part of a government or health group screening program<br>v. For indicating the presence of illicit drugs or alcohol, and non-health related purposes (e.g. paternity and ancestry)<br>vi. For indicating pregnancy, monitoring a pre-existing condition, testing for SARS-CoV-2 infection

Stage 2 - Developing a typology of clinical utility: As an emerging new area of research, there were no available or comparable typologies of clinical utility that could be applied as a framework to the DTC context. We used an abductive analytical approach to make sense of the data, which combines both inductive and deductive data analysis.(29) Inductive approach allows key concepts to emerge from data itself; and the application of pre-existing theories and knowledge deductively forms a more structured understanding of data.(29) The analysis was led by the first author with input from the multidisciplinary authorship team, which included experts in medical overuse (KB, SC, ARH) and pathology (ARH, DL, MS, ASJ). The data was firstly viewed as a whole, and then grouped and sorted into broader categories based on the key similarities and distinctions over several iterations. The grouping and sorting

For peer review only - http://bmjopen.bmj.com/site/about/guidelines.xhtml

process was informed by pre-existing concepts and our own prior knowledge of the medical overuse literature, especially in relation to the overuse of pathology tests. The following principles from the medical overuse literature most relevant for pathology tests were used to guide the analysis:

- The balance of potential harms and benefits of the test for the individual consumer is based on the potential harms and benefits using population level data.
- The scientific and analytical validity of the test as appropriate for the condition and/or purpose stated is based on published literature supporting scientific evidence, selected using the hierarchy of evidence as determined by the Oxford Centre for Evidence-based Medicine (OCEBM).(30)
- We prioritised any information identified by existing recommendations for or against specific tests by professional organisations, especially those developed to promote greater test stewardship (e.g. NICE, Choosing Wisely).

We moved iteratively back and forth between data and theory to change or add new inclusion/exclusion criteria to the groupings until no new categories emerged, and there was consensus between the authors (see Supplemental Figure 1). The allocation of tests into each category was supported by a literature search of evidence-based medicine (see Supplement 1). The final categories were determined by the following levels of distinctions: The first key distinction made was whether there is sufficient evidence-base to support the validity of the test as a diagnostic or screening tool for the stated condition it tests for. Secondly, within the evidence-based tests, there was a further distinction between those with and without a professional recommendation for screening the condition among specific populations. In the non-evidence-based tests, there was a distinction between those that aimed to detect a disease and those that aimed to promote general health. Thirdly, within the evidence-based tests with no recommendations for screening, tests that are ‘recommended against’ by known

For peer review only

professional guidelines aimed at preventing medical overuse were highlighted, followed by tests that have other medical overuse concerns, for example if the test method itself has low specificity or sensitivity, or that the condition being tested for has low pre-test probability. Within the non-evidence-based tests for disease, a distinction was made between tests that were non-evidence based in themselves, and tests used to diagnose conditions that were not recognised by the general medical community. In total, four categories emerged, with two of the categories (2 and 4) having three and two subcategories respectively.

<insert Supplemental Figure 1>

Patient and public involvement

Patients and the public will be involved in the dissemination plans of our research: The study results will be promoted publicly with health consumer partners and advisors from the Consumer Reference Group of the Wiser Healthcare, a National Health and Medical Research Council Centre for Research Excellence, the Consumers Health Forum of Australia, and Health Consumers New South Wales.

3. Findings

A total of 484 DTC tests were identified. Collectively these targeted 103 unique medical conditions, analytes or test products. Tests ranged from AU$12.99 to AU$1947 in cost (mean $197.83; median $148.50). There were 177 (36.6%) home self-tests, 65 (13%) self-collected DAPT, and 242 (51%) lab-collected DAPT.

3.1. Categories of clinical utility

1) Category 1: Tests with potential clinical utility (10.7% of total)

For peer review only - http://bmjopen.bmj.com/site/about/guidelines.xhtml

Just over one tenth (10.7%) of total products were allocated to Category 1 (Table 2). These were evidence-based tests recommended for screening in specific population groups by a recognised professional medical organisation, determined by a reported evidence-based medicine (EBM) approach. The most common examples were tests for sexually transmitted infections (STI), for which stigma contributes to barriers to clinical care, especially among communities at higher risk. In our study, DTC tests for STI tended to come in the form of home self-testing kits, which are lower in cost compared to DAPT and more likely to report test accuracy. Studies in Australia and internationally show that undertested at-risk populations are more likely to use online and home-based testing for STI than at primary care settings.(31) (32) DTC tests in this category may improve the rate of beneficial diagnosis and treatment in high-risk populations, especially those with barriers to clinical care. However, the tests will have limited utility for asymptomatic and low-risk populations outside of populations recommended for routine testing. And even for high-risk people, clinical follow-up is still needed to ensure timely treatment. This is especially notable since Category 1 tests are the least likely to offer pre- and post-test consultation.

Table 2: Category 1 results, Tests with potential clinical utility

Inclusion criteria	Target condition or test purpose	Key analyte or testing method	Mode of test#				Min. cost	Max. cost
			H	S	L	TN		
An established and recognised part of the diagnostic, screening or risk assessment pathway for the named medical condition or risk factor of a condition the product purports to test for.	Bowel cancer	Faecal occult blood	0	1	1	**2**	69.00	129.00
	Cardiovascular disease risk factors	Lipoprotein	0	0	3	**3**	198.00	330.00
	Chlamydia	Chlamydia trachomatis antigen	4	0	0	**4**	24.95	55.29
	Cholesterol level	Blood cholesterol	1	1	1	**3**	45.00	84.00
	Diabetes	HbA1c, glucose	0	2	4	**6**	35.00	78.00

For peer review only - http://bmjopen.bmj.com/site/about/guidelines.xhtml

Recommended for screening in specific population groups by a recognised professional medical organisation, determined by a reported evidence-based medicine (EBM) approach.	Gonorrhoea	Neisseria gonorrhoea antigen	3	0	0	**3**	34.95	56.71
	Hepatitis B	Hepatitis B surface antigen	4	0	0	**4**	24.95	56.71
	Hepatitis C	Hepatitis C virus antibody	2	0	0	**2**	56.71	92.40
	HIV	HIV-1 & HIV-2 antibody	11	0	0	**11**	34.95	138.00
	Sexually transmitted diseases - multiple tests	Chlamydia antigen, Gonorrhoea antigen, Hepatitis B antigen, Syphilis	2	0	7	**9**	64.00	188.00
	Syphilis	Treponemal Antibody	4	0	0	**4**	24.95	56.71
	Trichomoniasis infection	Trichomonas vaginalis lipophosphoglycan antibody	1	0	0	**1**	56.71	

[#]Mode of access: H=home self-tests, S= self-collected DAPT, L=Lab-collected DAPT, TN=total number).

2) *Category 2: Tests with limited clinical utility (30.6%)*

While tests in Category 2 (Table 3) have a legitimate role in diagnosis or treatment in clinical settings for the condition they target, the context in which these are marketed as DTC tests bring up several concerns for medical overuse. Firstly, about half in this category (designated as Subgroup 2A, 19.4% of total), were tests identified by well-known guidelines that aim to reduce medical overuse, such as the Choosing Wisely 'Do Not Do' recommendations and the, NICE 'Do Not Do' Recommendation, and 'Screening tests of unproven benefit' identified by the Royal Australasian College of General Practitioners (RACGP)'s 'Guidelines for preventative activities in general practice'.(33-35) Examples include prostate-specific antigen and MTHFR gene testing. Other tests in this category were more likely to generate high false positive rates because of the limited accuracy of the testing method for the target condition (Subgroup 2B, 9.3% of total). Examples include urine dipsticks for kidney and liver disease, which are often used in point-of-care screening before further investigation; whereas when used by consumers at home, false positive results may cause unnecessary distress and trigger

For peer review only - http://bmjopen.bmj.com/site/about/guidelines.xhtml

further GP visits. Thirdly, a small proportion were tests for conditions with low pre-test probability in the general population(36) (Subgroup 2C, 1.9%). This means the potential for producing high rates of false positives or misattributed diagnosis. For example, an IgG test for Cytomegalovirus is of limited utility because a high proportion of the general population will carry antibodies, but the pre-test probability for an active infection causing clinically significant disease is usually low. Even though the test may correctly show positive results from past vaccination or infection, it has limited utility in diagnosis, especially if symptoms are vague or within normal limits.

Table 3: Category 2 results, Tests with limited clinical utility

Inclusion criteria	Target condition or test purpose	Key analyte or testing method	Mode of test[#]				Min. cost	Max. cost
			H	S	L	TN		
**Subgroup 2A** *(n=94, 19.4% of total)*								
Identified as potentially contributing to medical overuse in certain populations by professionally recognised and evidence-based medicine (EBM) recommendations that aim to prevent medical overuse (e.g. Choosing Wisely, NICE 'Do Not Do' Recommendations)	Alzheimer's disease	APOE genotype	0	1	1	**2**	165.00	270.00
	Arthritis	Rheumatoid Factor	0	0	2	**2**	44.00	130.00
	Autoimmune disease	HLA-B27 antibody	0	0	2	**2**	69.00	120.00
	Cancer markers	Carcinoembryonic antigen (CEA)	0	0	3	**3**	80.00	160.00
	Cancer risk factors	Glutathione S-transferases genes	0	0	2	**2**	313.00	313.00
	Cardiovascular disease risk factors	Homocysteine	0	0	1	**1**	747.00	
	Cardiovascular disease risk factors	MTHFR & APOE genetic test	0	1	0	**1**	199.00	
	Coeliac disease	HLA gene typing	0	0	1	**1**	169.00	
	Food allergy	IgE	0	2	5	**7**	90.00	430.00
	Genital herpes	Antibodies	3	0	0	**3**	63.00	140.40
	Haemochromatosis	HFE gene mutation	0	0	2	**2**	69.00	159.00
	Heavy metal exposure	Heavy metal in blood	0	0	3	**3**	118.00	189.00
	Immune disorders	Immunogenetics	0	1	0	**1**	440.00	
	Insulin resistance	Fasting glucose, fasting Insulin	0	0	2	**2**	69.00	190.00
	Iron deficiency	Iron studies	0	1	8	**9**	58.00	318.00

For peer review only - http://bmjopen.bmj.com/site/about/guidelines.xhtml

	Low-grade Inflammation	High sensitivity CRP	0	0	1	**1**	142.00	
	MTHFR gene mutation	MTHFR gene mutation	0	4	5	**9**	50.00	520.00
	Neuro genetic disorders	Neurogenetics	0	1	0	**1**	647.00	
	Prostate cancer	Prostate-specific antigen	0	0	2	**2**	39.00	40.00
	Thyroid disease	Thyroid hormones	1	7	15	**23**	35.00	328.00
	Urinary tract infection	Urine culture & microscopy	0	0	3	**3**	36.00	50.00
	Vitamin B deficiency	Vitamin B12, B6, B9	0	0	6	**6**	65.00	298.00
	Vitamin D deficiency	Vitamin D	2	4	1	**7**	35.00	175.00
	Vitamin K deficiency	Vitamin K	0	1	0	**1**	280.00	
**Subgroup 2B *(n=45, 9.3% of total)***								
The testing method used has low sensitivity (limited ability to correctly predict disease) or low specificity (limited ability to correctly rule out disease) or is not reliable as a diagnostic test when used on its own. To reach an accurate diagnosis for the named condition, the gold standard diagnosis, and/or follow-up or parallel tests would be required.	Blood clotting	Prothrombin time	0	0	1	**1**	55.00	
	Chronic fatigue	Iron, Vitamin B12, thyroid hormones	0	0	1	**1**	145.00	
	Coeliac disease	IgA & IgG	0	0	4	**4**	69.00	232.00
	Gastrointestinal candida	IgG & IgM	0	1	0	**1**	240.00	
	Giardia	Lambia rapid antigen test	1	0	0	**1**	106.80	
	Helicobacter pylori	Stool antigen	1	2	1	**4**	90.00	125.00
	Inflammatory bowel disease	Faecal calprotectin	0	1	4	**5**	75.00	95.00
	Kidney disease	Albumin/ Creatinine Ratio dipstick	2	0	2	**4**	39.00	69.60
	Liver disease	Bilirubin dipstick	1	1	2	**4**	35.99	65.00
	Measles, mumps, rubella serology (indicating past infection)	Antibodies	0	0	4	**4**	49.00	90.00
	Pancreatic insufficiency	Faecal pancreatic elastase 1	0	0	1	**1**	89.00	
	Pancreatitis	Lipase, amylase	0	0	2	**2**	59.00	62.00

For peer review only

For peer review only - http://bmjopen.bmj.com/site/about/guidelines.xhtml

	Urinary tract infection	Urine dipstick (Nitrite and leukocyte esterase)	10	0	0	**10**	25.99	215.00
	Vaginal infection	Vaginal pH	6	0	0	**6**	12.99	14.95
**Subgroup 2C** ***(n=9, 1.9% of total)***								
The test is for a medical condition that has a low pre-test probability. Investigating clinically significant disease without clinician guidance or assessment of symptoms and medical history can drive false positive results.	Cytomegalovirus (CMV)	IgC & IgM antibody	0	0	1	**1**	59.00	
	Epstein-Barr virus (EBV)	EBV Antibody	0	0	1	**1**	59.00	
	Glucose-6-Phosphate-Dehydrogenase (G6PD) deficiency	G6PD enzyme assay	0	0	1	**1**	299.00	
	Lyme disease	Borrelia antibody	0	0	5	**5**	59.00	903.00
	Rotavirus	Rapid antigen test	1	0	0	**1**	92.40	

[#]Mode of access: H=home self-tests, S= self-collected DAPT, L=Lab-collected DAPT, TN=total number).

3) Category 3: Non-evidence-based commercial 'health checks' (41.9%)

Category 3 (Table 4) were non-evidence-based commercial 'health checks', making up the largest proportion of all tests in this study, at 41.9% of total. This finding suggests that healthy people form the largest target of the DTC test marketing. These tests may sometimes be justifiable if offered in a clinical setting as part of investigating symptoms or disease monitoring (e.g. testosterone hormone tests for hypogonadism). However there is negligible evidence to support their clinical utility as evaluative tests for health status among healthy populations.(37) A health check could be potentially useful if clinically relevant abnormalities are able to be detected. However, the tests identified in Table 4 are not used for investigating symptoms of a known medical condition. Furthermore, there are no agreed protocols or harmonised range to interpret whether the test results fall into a healthy or pathological range for a particular individual (the reference interval). Testing asymptomatic and low-risk

For peer review only

For peer review only - http://bmjopen.bmj.com/site/about/guidelines.xhtml

populations can also sometimes result in the incidental detection of conditions or risk factors that may not benefit from diagnosis or treatment.

Table 4: Category 3 results, Non-evidence-based commercial 'health checks'

Inclusion criteria	Target condition or test purpose	Key analyte or testing method	Mode of test[#]				Min. cost	Max. cost
			H	S	L	TN		
Evaluates normally existing biomarkers or health status in healthy populations; Does not aim at detecting, diagnosing or monitoring of a specific disease or medical condition	Biological age	Glycans	0	1	0	**1**	599.00	
	Bone health	Serum alkaline, phosphatase, calcium, potassium	0	0	2	**2**	80.00	125.00
	Coenzyme Q10 profile	Coenzyme Q10 level	0	0	1	**1**	180.00	
	Cytokine profile	Cytokine level	0	0	1	**1**	425.00	
	Fertility status (male)	Semen analysis	0	0	1	**1**	75.00	
	Gastrointestinal function	Microbiome analysis	0	11	1	**12**	109.00	1,199.00
	Gastrointestinal function	Stool analysis	0	14	0	**14**	120.00	490.00
	Health & wellness profile	Panel consisting of tests for more than one of: Full blood count, hormones, organ function, vitamins & minerals	1	0	48	**49**	18.95	749.00
	Hormone profile - female fertility status	Panel consisting of tests for more than one of: Follicle Stimulating Hormone, luteinising hormone, oestradiol, progesterone, testosterone	0	1	13	**14**	59.00	165.00
	Hormone profile - female health	Panel consisting of tests for more than one of: DHEAs, estrone, SHBG, oestradiol, estriol, progesterone, testosterone, androstenedione	0	11	1	**12**	54.00	410.00
	Hormone profile - general health	Panel consisting of tests for more than one of:	0	10	9	**19**	59.00	239.00

For peer review only - http://bmjopen.bmj.com/site/about/guidelines.xhtml

		adrenal, growth, thyroid & reproductive hormones							
	Hormone profile - male health	Panel consisting of tests for more than one of: DHEA, estrone, oestradiol, testosterone, SHBG, androstenedione	0	8	3	**11**	50.00	270.00	
	Hormone profile - menopause	Panel consisting of tests for more than one of: oestradiol, estriol, estrone, DHEA	0	2	2	**4**	81.00	179.00	
	Hormone profile - ovarian reserve	Anti-Mullerian Hormone	0	0	3	**3**	98.00	299.00	
	Hormone profile - sleep quality	Melatonin & cortisol	0	5	1	**6**	909.00	189.00	
	Hormone profile - sports & fitness performance	Panel consisting tests for more than one of: Growth & adrenal hormones	0	1	14	**15**	59.00	239.00	
	Hormone profile - stress	Salivary cortisol	0	10	0	**10**	23.00	165.00	
	Nutrigenomic profile	Genetics	0	2	0	**2**	249.00	550.00	
	Nutritional status	Amino acids	0	2	0	**2**	270.00	370.00	
	Nutritional status	Ammonia	0	0	1	**1**	153.00		
	Nutritional status	Chromium	0	0	1	**1**	108.00		
	Nutritional status	Copper	0	0	2	**2**	85.00	108.00	
	Nutritional status	Essential Fatty Acids	0	1	1	**2**	89.00	225.00	
	Nutritional status	Glutathione	0	0	1	**1**	335.00		
	Nutritional status	Magnesium	0	0	1	**1**	138.00		
	Nutritional status	Organic acids	0	8	0	**8**	74.95	597.00	
	Nutritional status	Selenium	0	0	1	**1**	108.00		
	Nutritional status	Iodine	0	1	0	**1**	155.00		
	Nutritional status	Zinc	0	0	2	**2**	75.00	170.00	
	Secretor status (blood typing)	Antigens	1	1	0	**2**	89.95	195.00	

[#]Mode of access: H=home self-tests, S= self-collected DAPT, L=Lab-collected DAPT, TN=total number).

4) Tests whose methods and/or target condition are not recognised by the general medical community (16.7%)

For peer review only - http://bmjopen.bmj.com/site/about/guidelines.xhtml

While Category 4 tests (Table 5) were also non-evidence-based (16.7%), they were condition-specific. We divided these into two subgroups to differentiate how non-evidence based tests might present different concerns: Firstly, tests that are in themselves non-evidence based, and secondly, the condition tested for is non-evidence based. For Subgroup 4A, without a reliable evidence base to support protocols for diagnosis and treatment, unproven DTC tests can be used to justify treatments for medical conditions that are not clinically recognised,(38) which can lead to further harms. In using tests in Subgroup 4B, consumers may be led to believe that they have a condition that requires treatment when there is little evidence to support this. Tests in this subcategory can be used to promote conditions that are not recognised by the medical community and the on-sale of associated non-evidence-based treatments, also known as 'disease mongering'.(39) Most notably, these unproven tests are often high in cost, including the most expensive item found in the review, at AU$1947.

Table 5: Category 4 results, Tests whose methods and/or target condition are not recognised by the general medical community

Inclusion Criteria	Target condition or test purpose	Key analyte or testing method	Mode of test#				Min. cost	Max. cost
			H	S	L	TN		
Subgroup 4A *(n= 70, 14.5% of total)*								
Current evidence does not support the clinical validity of the testing method for the condition it aims to test for.	Environmental toxin exposure	Hair metal & mineral analysis	0	4	0	**4**	175.00	325.00
	Environmental toxin exposure	Mycotoxin test	0	4	0	**4**	535.00	735.00
	Food allergy	Hair analysis	0	6	0	**6**	29.00	297.00
	Food allergy	IgA	2	0	0	**2**	380.00	
	Food allergy	IgG	0	10	12	**22**	162.00	550.00
	Food sensitivity & intolerance	ALCAT	0	13	0	**13**	325.00	1,947.00
	Hair loss	Hormones	0	0	2	**2**	225.00	225.00
	Heavy metal exposure	Urine chelation	0	5	0	**5**	225.00	485.00
	Histamine intolerance	Whole blood histamine	0	0	3	**3**	69.00	325.00
	Liver detoxification	Urine metabolites	0	1	0	**1**	225.00	

For peer review only - http://bmjopen.bmj.com/site/about/guidelines.xhtml

	Mental health nutrition (Pfeiffer test)	Zinc, Copper, Zinc-Copper ratio	0	0	2	**2**	338.00	531.00
	Cancer, Autism	Nagalese in blood	0	0	1	**1**	303.00	
	Mental health disorders	Neurotransmitters in urine	0	1	0	**1**	360.00	
	Small intestinal bacterial overgrowth (SIBO)	Hydrogen & methane breath test	0	2	2	**4**	220.00	380.00
**Subgroup 4B** *(n=11, 2.3% of total)*								
Current evidence does not support the condition tested for as being established and recognised by the general medical community.	Adrenal fatigue	DHEA & Cortisol	0	4	1	5	110.00	255.00
	Intestinal permeability (Leaky Gut Syndrome)	Lactulose & mannitol; stool analysis	0	4	0	4	123.00	622.80
	Paediatric autoimmune neuropsychiatric disorders syndrome (PANDAS)	Antibody	0	0	1	1	1,700.00	
	Mental health conditions/Pyrrole disorder	Hydroxyhaemopyrrol in-2-one (HPL) & neurotoxin	0	1	0	1	199.00	

#Mode of access: H=home self-tests, S= self-collected DAPT, L=Lab-collected DAPT, TN=total number).

3.2. Average cost

The average cost of each category is statistically significantly different (p value <0.001). There is a relationship between higher clinical utility and lower cost, and vice versa. Category 1 has the lowest average cost of $77.16, and smallest range in cost ($24.95 - $330). Category 2 has more expensive tests with a mean cost of $138.44 (range of $13 to $903). Category 3 had an average cost of $206.08 (range of $18.95 – $1199) and Category 4 had the most expensive tests with an average cost of $363.11 (range of $13 to $1947).

3.3. Pre-and post-test consultation and quality assurance

Services such as pre- and post-test consultation are crucial to optimising appropriate test selection and utilisation. Supplemental Figure 2 shows more than half of all DTC tests (n=273, 56.4%) did not state whether any pre- or post-test counselling was offered. Only a

For peer review only

For peer review only - http://bmjopen.bmj.com/site/about/guidelines.xhtml

quarter of the products (n=123, 25.4%) included a post-test consultation with a healthcare professional, which may include a non-medically trained health practitioner.[1] Another 9.7% (n=47) offered a post-test GP telephone consultation to be purchased separately.

Supplemental Figure 3 shows that Category 1 tests, which were most likely to be clinically useful, were least likely to offer pre- and post-test consultation. Supplemental Figure 4 shows a breakdown of quality assurance information by test mode. More than half of home self-tests and self-collected DAPT did not report quality assurance information in the online advertisement. While all lab-collected DAPT were designated for analysis in a NATA-accredited laboratory, there was no detailed information on accreditation status. A very small number of self -collected DAPT were designated for analysis in an overseas laboratory.

<insert Supplemental Figure 2, Supplemental Figure 3, Supplemental Figure 4>

4. Discussion

The wide range and large volume of DTC tests sold online in Australia suggest a sizable and lucrative market, targeting large numbers of consumers, both with and without symptoms. Accessed in a distinctly different way than for clinician-facilitated tests, there are certain DTC tests with attributes that will provide greater consumer benefits compared to those obtained in mainstream healthcare. The greater availability of DTC tests for stigmatised, under-tested and infectious conditions, such as STIs can potentially complement mainstream healthcare by addressing issues of medical underuse.(40) However, only a small proportion of all DTC tests found in this study were of a type likely to provide a clear benefit (Category 1, 10.7%).

[1] Healthcare professional in Australia is defined as: (a) a medical practitioner, a dentist or any other kind of health care worker registered under a law of a State or Territory; or (b) a biomedical engineer, chiropractor, optometrist, orthodontist, osteopath, pharmacist, physiotherapist, podiatrist, prosthetist or rehabilitation engineer. Naturopaths and Nutritionists are only Health Professionals, with regard to the definition above, if they are registered under State or Territory law.

For peer review only - http://bmjopen.bmj.com/site/about/guidelines.xhtml

Meanwhile, the majority of tests found in this study would only be offered in mainstream healthcare if there were very clear clinical justifications, if offered at all. These included tests that were recommended against by professional organisations due to concerns of medical overuse, and an array of non-evidenced tests not recognised by the medical community, as well as tests designed for medical management but rebranded as 'health checks' for asymptomatic people. These types of tests with potential for medical overuse, as identified in our categories of clinical utility, suggest significant implications for harms to consumers and likely substantial unnecessary downstream costs for the healthcare system as a result of further tests and clinical care cascaded by the DTC tests results.

By offering tests not normally obtainable in mainstream clinical care, DTC tests can appear to offer advantages in addition to ease and convenience of access. People's familiarity with undergoing a pathology test, which are often the same procedures ordered by doctors albeit under specific clinical circumstances, adds an element of scientific legitimacy to DTC testing.(38) Other products canvassed in the study depend on the hype and innovative appeal of emerging technologies such as nutrigenomic, microbiome and glycan tests, even though these are yet to demonstrate clinical utility in real-world settings.(41) (42) In some cases DTC test results may be used to support diagnoses and treatments that are non-evidence based.(38) This may often target consumers on 'diagnostic odysseys' who are seeking answers for unexplained symptoms or unsatisfactory diagnosis in conventional medicine. For example, a number of tests in Category 4 were tests for gastrointestinal conditions, which are known to be difficult to diagnose even in a clinical setting.(8) However, providing non-evidence-based diagnosis and treatments may further harm consumers already in a vulnerable position.

The largest target of DTC tests though are consumers who are healthy and asymptomatic, providing an untapped market for these products.(43) Over 40% of the tests found in this study aim to assess a range of normal, everyday functions such as sleep, ageing, physical activity, and hormone levels as potential indicators of pathological abnormality. The

For peer review only - http://bmjopen.bmj.com/site/about/guidelines.xhtml

emerging wellness industry relies on the material data from these test results to support a pipeline of justifying and promoting the consumption of nutritional supplements and dietary advice.(44) (45) However, testing healthy populations without clear clinical justification will only normalise the notion that people are 'sick until proven healthy'.(14) The downstream consequence of this 'low-value' practice is the creation of unnecessary medical waste, and more opportunity for incidental and unnecessary diagnoses.

Another concern is the regulatory loophole that allows tests with greater implications for medical overuse to be offered as DAPTs, even though they cannot be supplied in the form of home self-tests in Australia. In a recent review of the *Therapeutic Goods (Excluded Purposes) Specification 2010*, tests for cancer and human genetics were considered too high risk and clinically complex to be used without professional guidance, and therefore cannot be supplied as DTC home self-tests.(1) Yet our study shows these tests were being sold as lab-collected or self-collected DAPT. They offer consumers an alternative channel of access to these 'higher risk' tests because they are processed in a laboratory rather than at home. The fact that most DAPT are processed at NATA accredited laboratories may reinforce their perceived trustworthiness. However, laboratories do not have the capacity to recognise whether the tests are the right ones for the right patient, nor can they influence how consumers and requesting DTC companies make clinical decisions based on test results. Furthermore, our findings suggest there is variation in the quality assurance and the provision of pre- and post-test consultation in DAPTs, and thus compromising the safety and benefits for consumers.

While our findings conclude that the majority of DTC tests currently on the market are unlikely to benefit the everyday consumer, recognisably, some products may be clinically useful if professional consultation and balanced information is provided. For example, there is emerging evidence to show that predictive genetic testing in the general population may lead to clinically useful early interventions if these are clearly available,(46) or have 'personal

For peer review only - http://bmjopen.bmj.com/site/about/guidelines.xhtml

utility' in guiding advance planning and assisting the coping with uncertain risk status.(47) However, these benefits are facilitated in highly-monitored clinical settings, where the assessment of risks, personal circumstances and medical history inform testing decisions, and when extensive professional counselling is provided. The risks and harms of ordering DTC tests, which the TGA review identifies as being particularly relevant for cancers and genetic testing, is that professional guidance and clinical monitoring is lacking. As the balance of harms and benefits of predictive genetic testing is still unclear for many chronic conditions, the call is for an ongoing development of guidelines, professional knowledge and practice in this field, to ensure caution and ethical considerations when offering such tests to asymptomatic, healthy individuals.(46, 48) DTC testing may by-pass much of these cautionary processes, which is where the danger lies for consumers. The potential harms of undergoing these DTC tests differ in nuance to those posed by non-evidence-based tests ('quacks') in Category 4, however, these products are often sold side-by-side by the same companies, which can mislead consumers.

However, our study shows that professional consultation is inconsistently provided, or incurs a separate cost to consumers. Currently, no policy, regulation or industry codes provide clear guidance on the quality and accessibility of this important aspect of testing.

While this study is set in Australia, the growing availability of DTC testing is a topic that is receiving increasing attention internationally. (2, 40, 49-51) Earlier studies in the US,(6) UK, (7) (8) and Europe(52) also show a large variety and volume of DTC tests on sale. However, what is available to consumers is time and jurisdiction-specific, due to the different IVD and laboratory regulations that influence the type and manner in which DTC tests can be sold and used.(2, 53) Nevertheless, the potential harms to consumers and the healthcare system, and the inadequacy of regulatory frameworks in managing these harms are shared concerns.(17, 54) This study has developed a systematic search strategy and an analytical

For peer review only - http://bmjopen.bmj.com/site/about/guidelines.xhtml

framework based on clinical utility that can be applied in other jurisdictions to better understand these issues.

To our knowledge, there are no available data in Australia on the extent to which the different types of DTC test are used, nor studies about user motivation or experience. Future research in these directions would inform the design of consumer-centred solutions to address potential harms and provide a better understanding of the social and cultural context of DTC test use.

There are some limitations to the study. The analysis by clinical utility is highly contextual and interpreted by the authors. We acknowledge the potential for the categories to overlap, and that there may be other approaches to analysis. The DTC tests included in this time-specific search may not be exhaustive, as product availability may change over time, and others may be advertised in different platforms or sold in pharmacies. DTC tests offered by private commercial clinics but which are not advertised as a directly-purchasable item online, as is often the case for genetic and fertility tests, were also not included. The likelihood is there are many more such tests being sold in the market. This is an overview of products available; data extraction did not include an in-depth qualitative assessment of the textual information in each advertisement. Further study of this aspect would provide insight into the quality and transparency of DTC test advertisement information.

For peer review only

4.1. Conclusion & recommendations

The growing number of DTC tests available for direct purchase online in Australia purport to empower consumers with choice and convenience of access. The categorisation of clinical utility developed in this study shows the majority of currently available products lack clear benefits to consumers. The large offering of tests seldom recommended in a clinical setting may represent an under-recognised driver of medical overuse and consumption of low-value healthcare, particularly amongst healthy consumers. Unlike publicly funded pathology

For peer review only - http://bmjopen.bmj.com/site/about/guidelines.xhtml

services and hospital data that are auditable, the harms triggered by DTC tests are not easily tracked and measured. This may mean it remains a relatively hidden problem, with consumers and the healthcare system bearing the brunt of the immediate and long-term costs.

At the policy and regulation level, higher standards of test quality and regulation are needed to support informed decision-making for consumers.(16) Under the new European IVD Regulation there are increasing requirements for IVD companies to provide data to the regulator not just on the quality and analytical performance of tests but also evidence on the clinical performance of the devices, as well as a need for post-market surveillance on the performance of the tests.(55) Similarly, in Australia, DTC tests, especially those in Category 4, should fall under the same scrutiny and regulation before they are allowed to enter the online market. Guidelines or position statements from NATA or the Royal College of Pathologists of Australasia for DTC tests - other than those already issued for genetic tests - would encourage best practice. Nevertheless, these professional organisation recommendations will be limited to the laboratory end of testing, rather than on consumer-facing marketing and promotion of DTC tests. At the industry level, a commitment to provide balanced information about the evidence-base and potential clinical utility for the right populations is needed; as is the assurance of pre- and post-testing consultation with appropriately qualified professionals. Consumers of varying levels of health literacy must be supported to critically engage and assess the applicability and suitability of different DTC tests. To ensure test results and patient safety are adequately followed up by medical professionals, the introduction of consumer-led reporting systems(40) such as digital registries of test outcomes similar to those used for COVID-19 RATs is recommended. Currently Choosing Wisely and NICE ‘Do Not Do’ guidelines are developed for clinical settings where clinicians and consumers are both involved; these could be adapted to support decision-making in the DTC context.

Acknowledgements

For peer review only - http://bmjopen.bmj.com/site/about/guidelines.xhtml

We would like to thank Tracy Walter and Isabel Serret for providing research assistance with data extraction, and Prof. David Handelsman for guidance on sources of information regarding clinical utility of pathology tests used in endocrinology.

Contributorship statement

PS conceptualised the study and led data collection, with theoretical contributions from KB, SC and FS. PD provided technical advice and analysis of the quantitative data. ARH, ASJ, DL and MS provided technical advice. All authors contributed to the data analysis and approved the submitted manuscript.

Human Research Ethics Approval

This study does not involve human participants.

Competing interests

No competing interest to declare.

Funding

This study received funding from the Department of Health and Aged Care, Australian Government, National Health and Medical Research Council, grant number #1104136 & #2006545

Data sharing statement

All data relevant to the study are included in the article or uploaded as supplementary information.

References

For peer review only - http://bmjopen.bmj.com/site/about/guidelines.xhtml

1. Therapeutic Goods Administration. Summary and outcomes: review of the regulation of certain self-testing in vitro diagnostic medical devices (IVDs) in Australia2020 September 2020.
2. Orth M, Vollebregt E, Trenti T, Shih P, Tollanes M, Sandberg S. Direct-to-consumer laboratory testing (DTCT): challenges and implications for specialists in laboratory medicine. Clinical Chemistry and Laboratory Medicine (CCLM). 2022.
3. Ayers AA. Understanding the benefits and risks of direct-to-consumer testing for urgent care. Journal of Urgent Care Medicine. 2021.
4. Ayala-Lopez N, Nichols JH. Benefits and Risks of Direct-to-Consumer Testing. Archives of Pathology & Laboratory Medicine. 2020;144(10):1193-8.
5. Drummond S. What are the potential outcomes of allowing Australian consumers direct access to community pathology services?: A review of patient initiated testing. Australian Journal of Medical Science. 2016;37(1):14-8.
6. Lovett KM, Mackey TK, Liang BA. Evaluating the evidence: direct-to-consumer screening tests advertised online. Journal of Medical Screening. 2012;19(3):141-53.
7. Ryan A, Wilson S, Greenfield S, Clifford S, McManus R, Pattison H. Range of self-tests available to buy in the United Kingdom: an Internet survey. Journal of Public Health. 2006;28(4):370-4.
8. Wong A, White H, Plant A, Shipman A, Shipman K. Issues surrounding consumer-bought food-allergy testing. Clinical and experimental dermatology. 2022;47(3):547-52.
9. Galior KD, Baumann NA. Challenges with at-home and mail-in direct-to-consumer testing: preanalytical error, reporting results, and result interpretation. Clinics in Laboratory Medicine. 2020;40(1):25-36.
10. Ho A, Quick O. Leaving patients to their own devices? Smart technology, safety and therapeutic relationships. BMC medical ethics. 2018;19(1):1-6.
11. Geransar R, Einsiedel E. Evaluating online direct-to-consumer marketing of genetic tests: informed choices or buyers beware? Genetic testing. 2008;12(1):13-23.
12. Copp T, Nickel B, Lensen S, Hammarberg K, Lieberman D, Doust J, et al. Anti-Mullerian hormone (AMH) test information on Australian and New Zealand fertility clinic websites: a content analysis. BMJ open. 2021;11(7):e046927.
13. Carter SM, Rogers W, Heath I, Degeling C, Doust J, Barratt A. The challenge of overdiagnosis begins with its definition. BMJ. 2015;350:h869.
14. Welch HG, Schwartz L, Woloshin S. Overdiagnosed: making people sick in the pursuit of health: beacon press; 2012.
15. McAlister S, Barratt AL, Bell KJ, McGain F. The carbon footprint of pathology testing. Medical journal of Australia. 2020;212(8):377-82.
16. McCartney M, Watson J, Finney B, Salisbury C. Why blood testing companies need effective regulation. British Medical Journal Publishing Group; 2022.
17. Wilkinson E. The rise of direct-to-consumer testing: is the NHS paying the price? bmj. 2022;379.
18. Bloss CS, Schork NJ, Topol EJ. Direct-to-consumer pharmacogenomic testing is associated with increased physician utilisation. Journal of medical genetics. 2014;51(2):83-9.
19. Giovanni MA, Fickie MR, Lehmann LS, Green RC, Meckley LM, Veenstra D, et al. Health-care referrals from direct-to-consumer genetic testing. Genetic Testing and Molecular Biomarkers. 2010;14(6):817-9.

For peer review only - http://bmjopen.bmj.com/site/about/guidelines.xhtml

20. Elshaug AG, Watt AM, Mundy L, Willis CD. Over 150 potentially low-value health care practices: an Australian study. Medical Journal of Australia. 2012;197(10):556-60.
21. Brodersen J, Schwartz LM, Heneghan C, O'Sullivan JW, Aronson JK, Woloshin S. Overdiagnosis: what it is and what it isn't. Royal Society of Medicine; 2018. p. 1-3.
22. National Health and Medical Research Council. Direct-to-Consumer Genetic Testing: A Statement from the National Health and Medical Research Council (NHMRC) 2014 [Available from: https://www.nhmrc.gov.au/sites/default/files/documents/reports/statement-consumer-genetic-testing.pdf.
23. Tandy-Connor S, Guiltinan J, Krempely K, LaDuca H, Reineke P, Gutierrez S, et al. False-positive results released by direct-to-consumer genetic tests highlight the importance of clinical confirmation testing for appropriate patient care. Genetics in Medicine. 2018;20(12):1515-21.
24. Stanczyk FZ, Mandelbaum RS, Lobo RA. Potential pitfalls of reproductive direct-to-consumer testing. F&S Reports. 2022;3(1):3-7.
25. McGuire AL, Evans BJ, Caulfield T, Burke W. Regulating direct-to-consumer personal genome testing. Science. 2010;330(6001):181-2.
26. Chauvin A, Lang E, Bossuyt P. When less is more: mitigating the harms and risks of over-testing through shared decision making. Canadian Journal of Emergency Medicine. 2022;24(2):126-7.
27. Cozza V, Hoang VT, Petrocchi M, De Nicola R, editors. Transparency in keyword faceted search: An investigation on google shopping. Digital Libraries: Supporting Open Science: 15th Italian Research Conference on Digital Libraries, IRCDL 2019, Pisa, Italy, January 31–February 1, 2019, Proceedings 15; 2019: Springer.
28. Ziakis C, Vlachopoulou M, Kyrkoudis T, Karagkiozidou M. Important factors for improving Google search rank. Future internet. 2019;11(2):32.
29. Kennedy B, Thornburg R. Deduction, induction, and abduction. In: Flick U, editor. Tthe SAGE Handbook of Qualitative Data Collection. Los Angeles: Sage; 2018. p. 49-64.
30. OCEBM Levels of Evidence Working Group. The Oxford Levels of Evidence 2. Oxford Centre for Evidence-Based Medicine; 2009.
31. Groos A, Peardon-Freeman S, McFarlane K, Braithwaite S, Gajjar D, Murch P, et al. Free online chlamydia and gonorrhoea urine test request in Queensland, Australia: convenience of home sample collection versus pathology collection centre attendance for faster results. Sexual Health. 2021;18(3):254-9.
32. Kersaudy-Rahib D, Lydié N, Leroy C, March L, Bébéar C, Arwidson P, et al. Chlamyweb Study II: a randomised controlled trial (RCT) of an online offer of home-based Chlamydia trachomatis sampling in France. Sexually transmitted infections. 2017;93(3):188-95.
33. Bhatia RS, Levinson W, Shortt S, Pendrith C, Fric-Shamji E, Kallewaard M, et al. Measuring the effect of Choosing Wisely: an integrated framework to assess campaign impact on low-value care. BMJ quality & safety. 2015;24(8):523-31.
34. Cadamuro J, Ibarz M, Cornes M, Nybo M, Haschke-Becher E, von Meyer A, et al. Managing inappropriate utilization of laboratory resources. Diagnosis. 2019;6(1):5-13.

For peer review only - http://bmjopen.bmj.com/site/about/guidelines.xhtml

35. Royal Australasian College of General Practitioners. Guidelines for Preventive Activities in General Practice, 9th Edition. East Melbourne, Vic.; 2016.
36. Lam JH, Pickles K, Stanaway FF, Bell KJ. Why clinicians overtest: development of a thematic framework. BMC Health Services Research. 2020;20(1):1-11.
37. Krogsbøll LT, Jørgensen KJ, Larsen CG, Gøtzsche PC. General health checks in adults for reducing morbidity and mortality from disease. Cochrane Database of Systematic Reviews. 2012(10).
38. Jones SL, Campbell B, Hart T. Laboratory tests commonly used in complementary and alternative medicine: a review of the evidence. Annals of Clinical Biochemistry. 2019;56(3):310-25.
39. Moynihan R, Heath I, Henry D. Selling sickness: the pharmaceutical industry and disease mongering. BMJ. 2002;324(7342):886-91.
40. Exten C, Pinto CN, Gaynor AM, Meyerson B, Griner S, Van Der Pol B. Direct-to-Consumer STI Testing Services: A Position Statement from the American Sexually Transmitted Diseases Association. Sexually transmitted diseases. 2021.
41. Fiala C, Taher J, Diamandis EP. P4 medicine or O4 medicine? Hippocrates provides the answer. The Journal of Applied Laboratory Medicine. 2019;4(1):108-19.
42. Floris M, Cano A, Porru L, Addis R, Cambedda A, Idda ML, et al. Direct-to-consumer nutrigenetics testing: an overview. Nutrients. 2020;12(2):566.
43. Shih P, Prokopovich K, Degeling C, Street J, Carter SM. Direct-to-consumer detection of atrial fibrillation in a smartwatch electrocardiogram: Medical overuse, medicalisation and the experience of consumers. Social Science & Medicine. 2022:114954.
44. Flores M, Glusman G, Brogaard K, Price ND, Hood L. P4 medicine: how systems medicine will transform the healthcare sector and society. Personalized medicine. 2013;10(6):565-76.
45. Bjelakovic G, Nikolova D, Gluud LL, Simonetti RG, Gluud C. Antioxidant supplements for prevention of mortality in healthy participants and patients with various diseases. Cochrane database of systematic reviews. 2012(3).
46. Paulsen JS, Nance M, Kim J-I, Carlozzi NE, Panegyres PK, Erwin C, et al. A review of quality of life after predictive testing for and earlier identification of neurodegenerative diseases. Progress in neurobiology. 2013;110:2-28.
47. Roberts JS, Patterson AK, Uhlmann WR. Genetic testing for neurodegenerative diseases: ethical and health communication challenges. Neurobiology of disease. 2020;141:104871.
48. Unim B, Pitini E, Lagerberg T, Adamo G, De Vito C, Marzuillo C, et al. Current genetic service delivery models for the provision of genetic testing in Europe: a systematic review of the literature. Frontiers in genetics. 2019;10:552.
49. Stoffel M, Greene DN, Beal SG, Foley P, Killeen AA, Shafi H, et al. Direct-to-consumer testing for routine purposes. Clinical chemistry. 2022;68(9):1121-7.
50. Ickenroth MH, Grispen JE, Ronda G, Dinant G-J, de Vries NK, van der Weijden T. Educating consumers in self-testing: the development of an online decision aid. Health Education Journal. 2015;74(4):485-95.
51. den Oudendammer WM, Broerse JE. Towards a decision aid for self-tests: Users' experiences in The Netherlands. Health Expectations. 2019;22(5):983-92.
52. Ronda G, Portegijs P, Dinant G-J, Buntinx F, Norg R, van der Weijden T. Use of diagnostic self-tests on body materials among Internet users in the Netherlands: prevalence and correlates of use. BMC Public Health. 2009;9(1):100.

For peer review only - http://bmjopen.bmj.com/site/about/guidelines.xhtml

53. Holmes DT. Self-Ordering Laboratory Testing: Limitations When a Physician Is not Part of the Model. Clinics in Laboratory Medicine. 2020;40(1):37-49.
54. Doan T-HP, Adamson PC, Klausner JD. Legislation to Increase Accessibility to Direct-to-Consumer Tests in California. Sexually Transmitted Diseases. 2022;49(1):e37.
55. The European Parliament. In vitro diagnostic medical devices, Regulation (EU) 2017/746 of the European Parliament and of the Council of 5 April 2017 on in vitro diagnostic medical devices and repealing Directive 98/79/EC and Commission Decision 2010/227/EU. 2022.

For peer review only

For peer review only - http://bmjopen.bmj.com/site/about/guidelines.xhtml

Supplemental Figure 1. Levels of distinctions in analysis

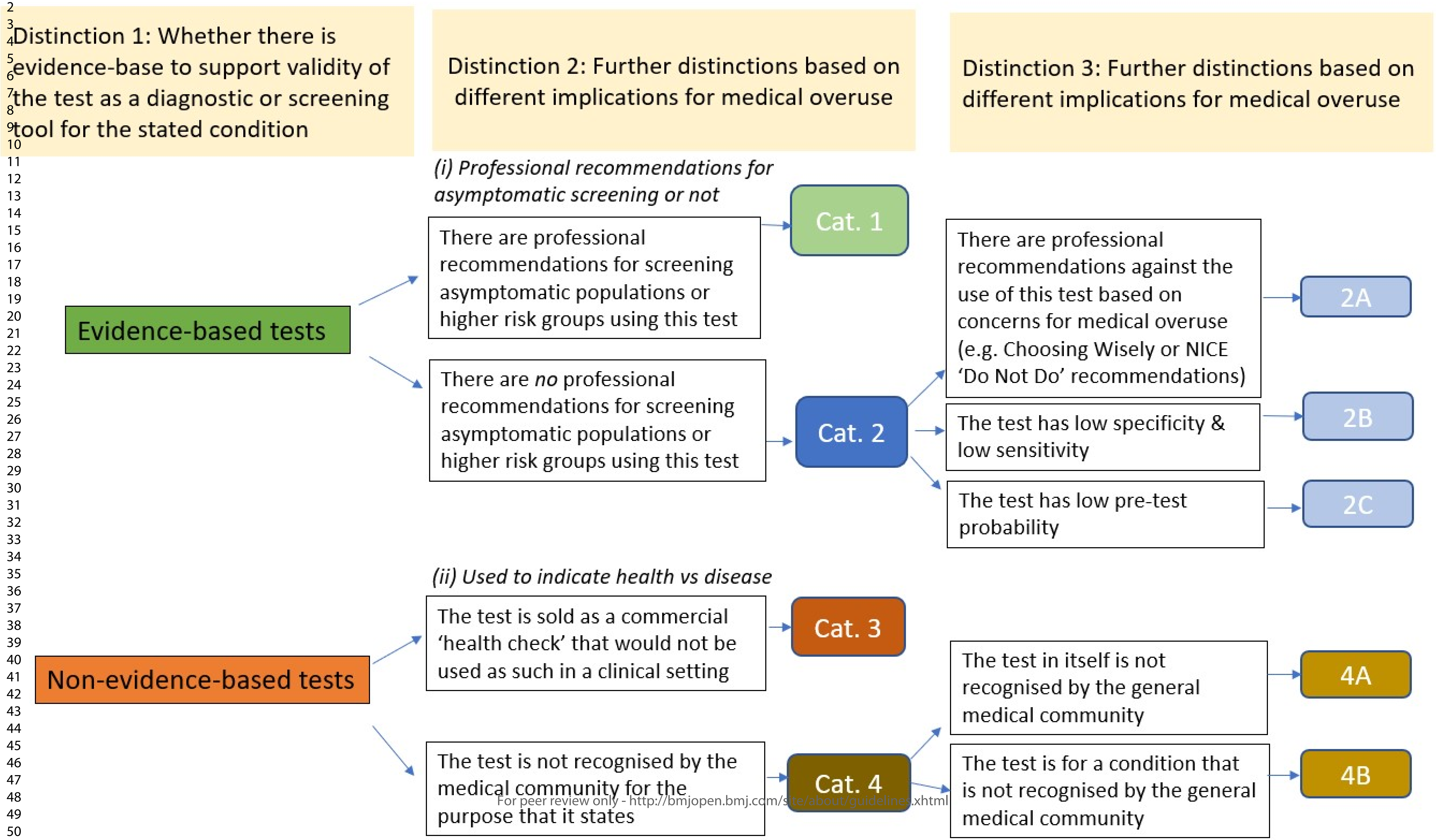

For peer review only - http://bmjopen.bmj.com/site/about/guidelines.xhtml

Supplemental Figure 2. Pre-& Post-test Consultation offered

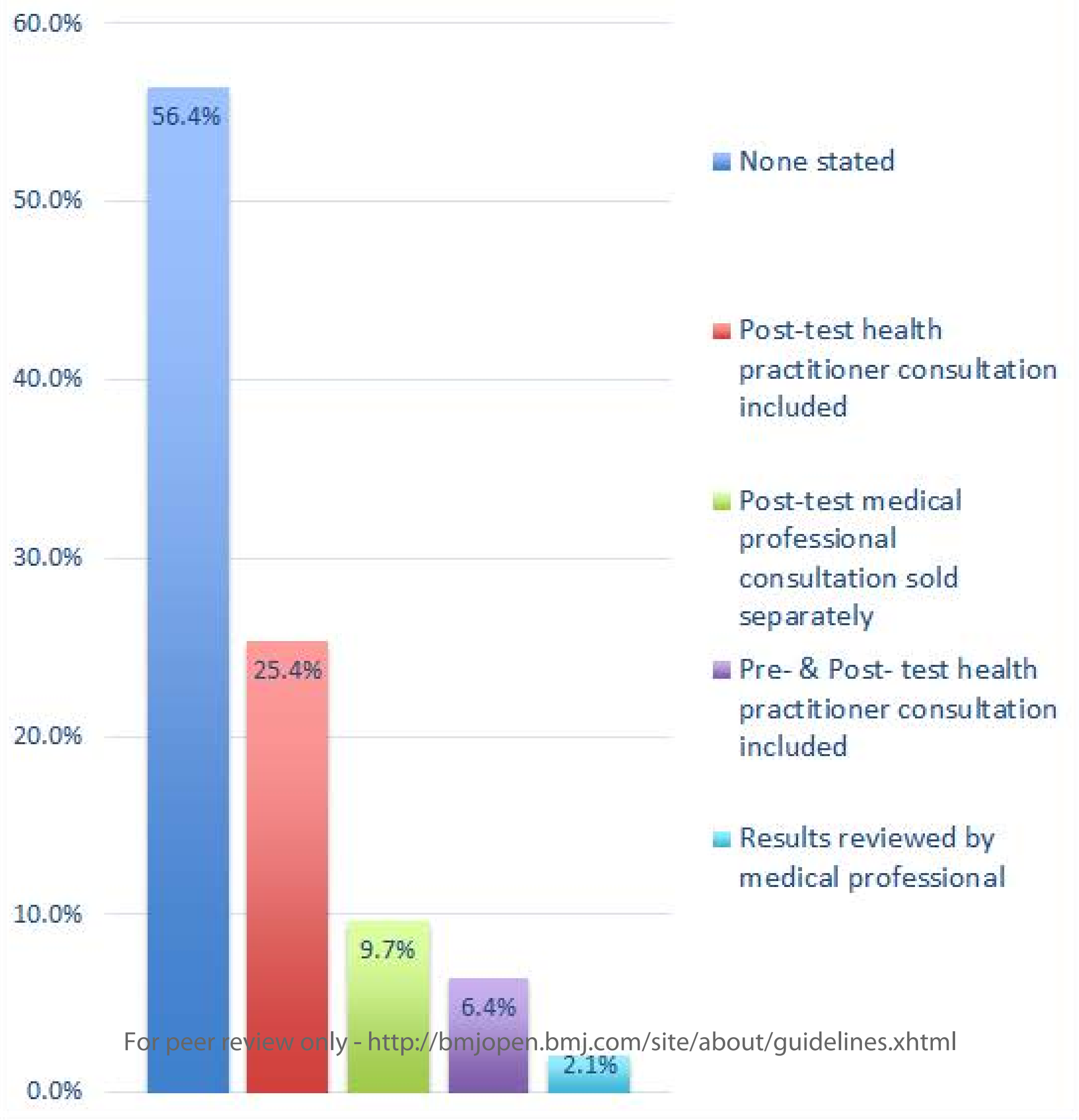

Supplemental Figure 3: Pre- & Post-test Consultation offered by Category of Clinical Utility

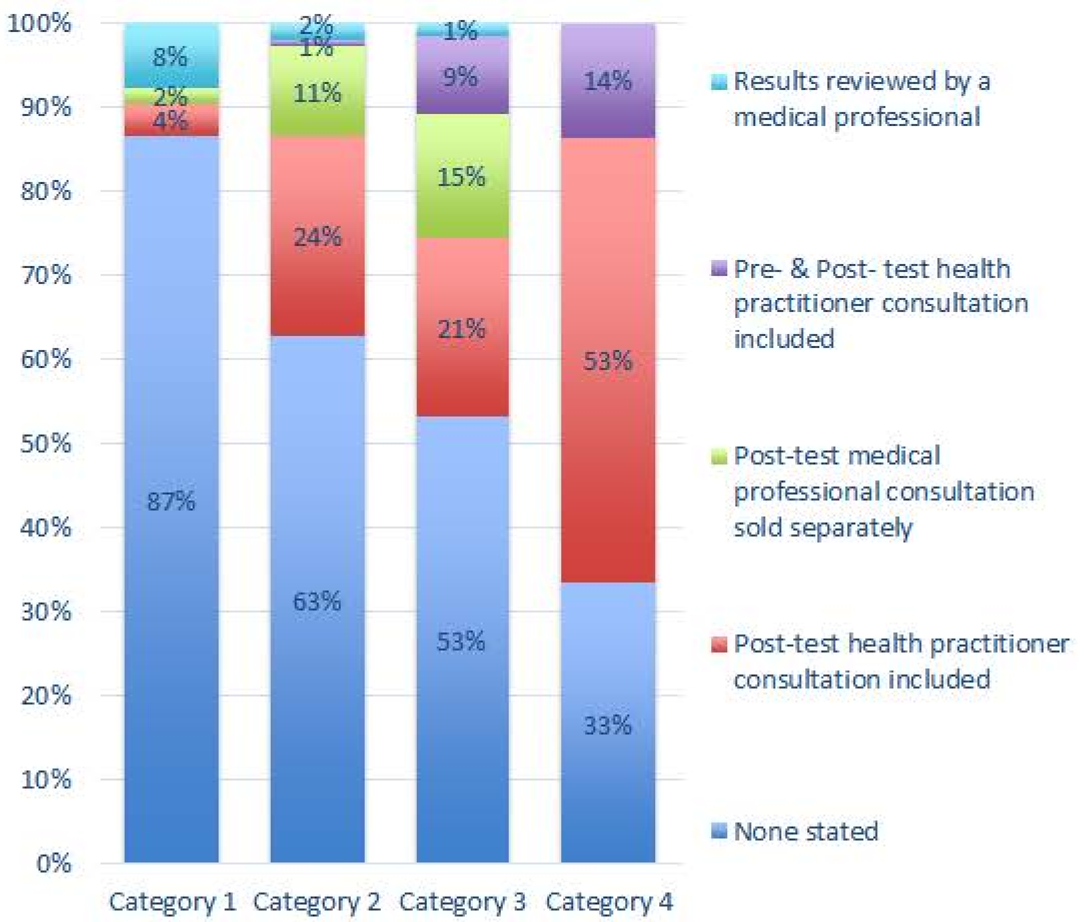

Supplemental Figure 4: Test Quality Assruance by Test Mode

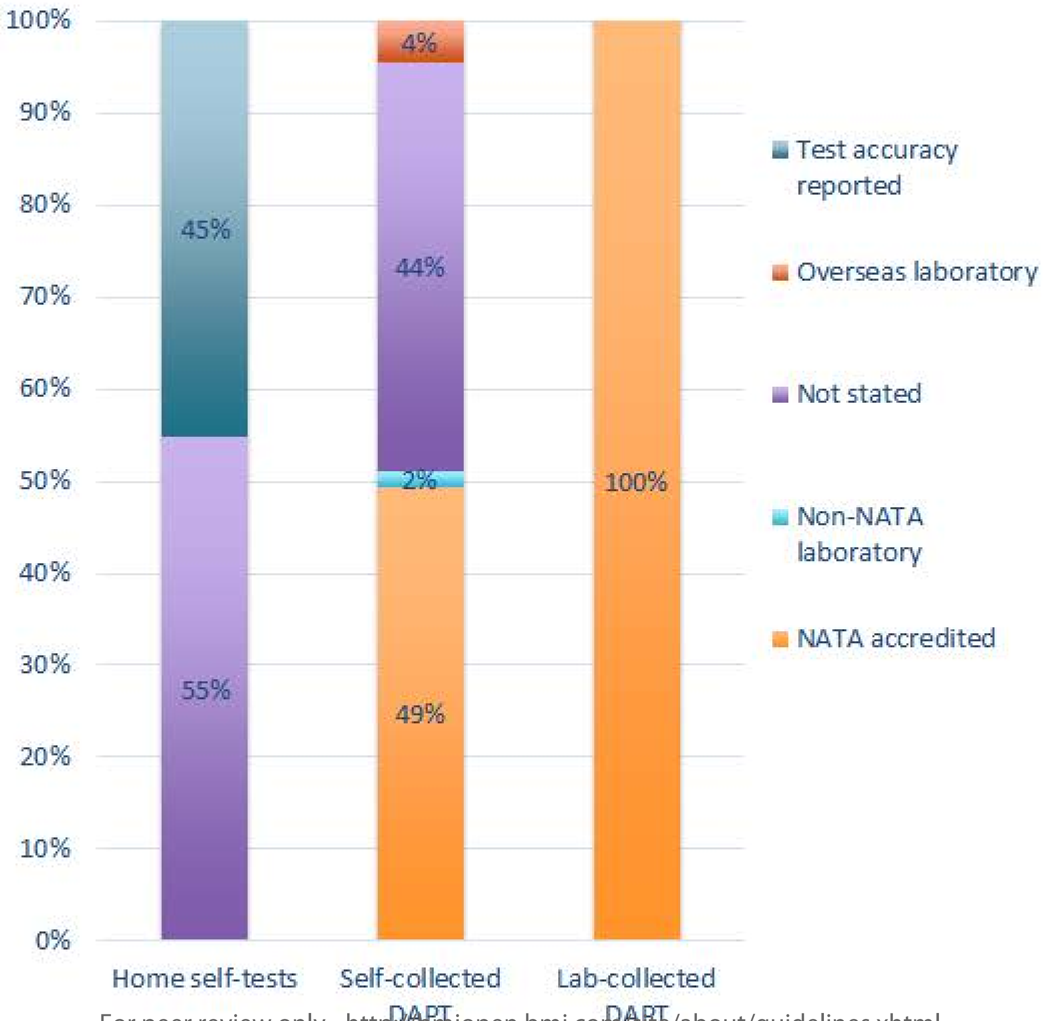

Supplement 1

The following table presents the evidence used to support the inclusion of the tests into respective Categories and Subgroups, and the search and inclusion strategy used.

1. Category 1: Tests with potential clinical utility

Evidence to support inclusion

The test is recommended for asymptomatic screening of specific population groups by a recognised professional medical organisation, determined by a reported evidence-based medicine (EBM) approach.

Search & inclusion strategy

1. For each test, a Google Advanced search using key words: (“*medical condition or test purpose*, *analyte and/or testing method – see column 1*”) AND Screen* OR test* AND Recommend* OR Guide* AND Australia.
2. The first 30 records were screened for relevance. We prefaced Australian and Australasian professional organisations first, and then international professional organisations. The country or region of origin of the recommendations/guidelines is reported in Column 2.

**Medical condition or test purpose; analyte and/or testing method**	**Reason for inclusion & Type of supporting evidence**	**Recommendation or summary of evidence**	**Reference**
Bowel cancer (faecal occult blood test)	Recommended as an asymptomatic screening test for certain population groups; Government organisation (Australia)	Bowel and colorectal cancer screening is recommended for Australians aged 50 - 74 by the National Bowel Cancer Screening Program (every two years).	Australian Government, Department of Health and Aged Care (2022) National Bowel Cancer Screening Program, https://www.health.gov.au/initiatives-and-programs/national-bowel-cancer-screening-program. Accessed 19 June 2022.
Cardiovascular disease risk factors (Lipoproteins)	Recommended as an asymptomatic screening test for certain population groups; Recognised professional group (Australasia)	Adults should have their blood lipids assessed every five years starting at 45 years of age. Lipid levels should be interpreted in the context of an absolute CVD risk assessment after 45 years of age (35 years of age for Aboriginal and Torres Strait Islander peoples). Aboriginal and Torres Strait Islander adults should have lipid tests performed every five years from 35 years of age.	Royal Australasian College of General Practitioners (RACGP) (2021) Guidelines for preventive activities in general practice https://www.racgp.org.au/getattachment/1ad1a26f-9c8b-4e3c-b45b-3237272b3a04/Guidelines-for-preventive-activities-in-general-practice.aspx. Accessed 12 June 2022.
Chlamydia (Chlamydia trachomatis antigen)	Recommended as an asymptomatic screening test for certain population groups; Recognised professional groups (Australasia)	Chlamydia testing should be included in a standard STI check-up (gonorrhoea, chlamydia, HIV, syphilis) for asymptomatic sexually active populations. Routine screening is also recommended for men who have sex with men, young (heterosexual) Aboriginal and Torres Strait Islander people living in remote and very	RACGP (2021) Guidelines for preventive activities in general practice https://www.racgp.org.au/clinical-resources/clinical-guidelines/key-racgp-guidelines/view-all-racgp-guidelines/guidelines-for-

		remote areas, and travellers returning from high prevalence areas overseas.	preventive-activities-in-general-pr/communicable-diseases/sexually-transmissible-infections. Accessed 12 June 2022. Australasian Society for HIV, Viral Hepatitis and Sexual Health Medicine (ASHM), Australian STI Management Guidelines (2022) https://sti.guidelines.org.au/standard-asymptomatic-checkup/ . Accessed 12 June 2022.
Cholesterol level (Blood Cholesterol)	Recommended as an asymptomatic screening test for certain population groups; Recognised professional group (Australasia)	Adults 45 and over have their blood cholesterol level assessed every five years. Lipid levels should be interpreted in the context of an absolute CVD risk assessment after 45 years of age (35 years of age for Aboriginal and Torres Strait Islander peoples; Aboriginal and Torres Strait Islander adults should have lipid tests performed every five years from 35 years of age. For those with established CHD, or those at high risk, screening should be yearly.	RACGP (2022) Guidelines for prevention activities in general practice, Cholesterol and other lipids, https://www.racgp.org.au/clinical-resources/clinical-guidelines/key-racgp-guidelines/view-all-racgp-guidelines/guidelines-for-preventive-activities-in-general-pr/prevention-of-vascular-and-metabolic-disease/cholesterol-and-other-lipids. Accessed 31 May 2022.
Diabetes (HbA1c, glucose)	Recommended as an asymptomatic screening test for certain population groups; Recognised professional group (Australasia)	Fasting glucose or glycated Hb is recommended every 3 years for people at high risk of Type 2 Diabetes (40+ and being overweight or obese, AUSDRISK score (questionnaire based risk calculator) of 12 or more. Consider screening for populations with increased risk, e.g. first-degree relative with diabetes, high-risk ethnicity, history of a previous cardiovascular event, women with history of gestational diabetes mellitus and polycystic ovary syndrome, patients on antipsychotic drugs); Those with impaired glucose tolerance test or impaired fasting glucose (not limited by age) should have fasting blood glucose tested every 12 months.	RACGP (2021) Guidelines for preventive activities in general practice https://www.racgp.org.au/getattachment/1ad1a26f-9c8b-4e3c-b45b-3237272b3a04/Guidelines-for-preventive-activities-in-general-practice.aspx. Accessed 12 June 2022.
Gonorrhoea (Neisseria gonorrhoea antigen)	Recommended as an asymptomatic screening test for certain population groups; Recognised professional group (Australasia)	Gonorrhoea should be included in a standard STI check-up (gonorrhoea, chlamydia, HIV, syphilis) for asymptomatic sexually active populations. Routine screening is also recommended for men who have sex with men, young (heterosexual) Aboriginal and Torres Strait Islander people living in remote and very remote areas, and travellers returning from high prevalence areas overseas.	RACGP (2021) Guidelines for preventive activities in general practice https://www.racgp.org.au/clinical-resources/clinical-guidelines/key-racgp-guidelines/view-all-racgp-guidelines/guidelines-for-preventive-activities-in-general-pr/communicable-diseases/sexually-transmissible-infections. Accessed 12 June

For peer review only

			2022.<br>Australasian Society for HIV, Viral Hepatitis and Sexual Health Medicine (ASHM), Australian STI Management Guidelines (2022)<br>https://sti.guidelines.org.au/standard-asymptomatic-checkup/. Accessed 12 June 2022.
Hepatitis B (Hepatitis B surface antigen)	Recommended as an asymptomatic screening test for certain population groups; Recognised professional group (Australasia)	Most people with chronic hepatitis B are asymptomatic unless their disease progresses (latent Hepatitis B). Opportunistic screening is recommended 1for high-risk groups (pp. 20) including people born in countries with a high prevalence, and Aboriginal and Torres Strait Island peoples.	ASHM, Testing Policy - Hepatitis B (2022) https://testingportal.ashm.org.au/files/ASHM_TestingPolicy_2020_HepatitisB_07_2.pdf. Accessed 12 June 2022.
Hepatitis C (Hepatitis C virus antibody)	Recommended as an asymptomatic screening test for certain population groups; Recognised professional group (Australasia)	Hepatitis C virus (HCV) testing should be performed as part of STI testing in people living with HIV, current HIV pre-exposure prophylaxis (PrEP) use, history of injecting drug use, anal sex with a partner with HCV infection, incarceration, non-professional tattoos or body piercings or receipt of organs or blood products before 1990. Asymptomatic screening is *not* recommended for other populations.	ASHM, Australian STI Management Guidelines (2022)<br>https://sti.guidelines.org.au/standard-asymptomatic-checkup/. Accessed 12 June 2022.
HIV (HIV-1 & HIV-2 antibody)	Recommended as an asymptomatic screening test for certain population groups; Recognised professional group (Australasia)	HIV should be included in a standard STI check-up (gonorrhoea, chlamydia, HIV, syphilis) for asymptomatic sexually active populations. Routine screening is also recommended for men who have sex with men, young (heterosexual) Aboriginal and Torres Strait Islander people living in remote and very remote areas, and travellers returning from high prevalence areas overseas.	RACGP (2021) Guidelines for preventive activities in general practice<br>https://www.racgp.org.au/clinical-resources/clinical-guidelines/key-racgp-guidelines/view-all-racgp-guidelines/guidelines-for-preventive-activities-in-general-pr/communicable-diseases/sexually-transmissible-infections. Accessed 12 June 2022.<br>Australasian Society for HIV, Viral Hepatitis and Sexual Health Medicine (ASHM), Australian STI Management Guidelines (2022)<br>https://sti.guidelines.org.au/standard-asymptomatic-checkup/. Accessed 12 June 2022.
Syphilis (Treponemal antibody)	Recommended as an asymptomatic screening test for certain population groups; Recognised professional group (Australasia)	Syphilis screening should be included in a standard STI check-up (gonorrhoea, chlamydia, HIV, syphilis) for asymptomatic sexually active populations. Routine screening is also recommended for men who have sex with men, young	RACGP (2021) Guidelines for preventive activities in general practice<br>https://www.racgp.org.au/clinical-resources/clinical-guidelines/key-racgp-

For peer review only - http://bmjopen.bmj.com/site/about/guidelines.xhtml

		(heterosexual) Aboriginal and Torres Strait Islander people living in remote and very remote areas, and travellers returning from high prevalence areas overseas.	guidelines/view-all-racgp-guidelines/guidelines-for-preventive-activities-in-general-pr/communicable-diseases/sexually-transmissible-infections. Accessed 12 June 2022.<br>Australasian Society for HIV, Viral Hepatitis and Sexual Health Medicine (ASHM), Australian STI Management Guidelines (2022)<br>https://sti.guidelines.org.au/standard-asymptomatic-checkup/. Accessed 12 June 2022.
Trichomoniasis infection (Trichomonas vaginalis lipophosphoglycan antibody)	Recommended as an asymptomatic screening test for certain population groups; Recognised professional group (Australasia)	Asymptomatic screening for trichomoniasis is only recommended in certain population groups and situations, e.g. in older people, people from regional and remote areas, especially Aboriginal and Torres Strait Islander people and street-based sex workers. Trichomoniasis is endemic in some Aboriginal and Torres Strait Islander populations in regional/remote areas and people should be tested according to local guidelines.	Australasian Society for HIV, Viral Hepatitis and Sexual Health Medicine (ASHM), Australian STI Management Guidelines (2022)<br>https://sti.guidelines.org.au/sexually-transmissible-infections/trichomoniasis/. Accessed 12 June 2022.

2. Category 2, Subgroup 2A: Tests with limited clinical utility - identified by experts as potentially contributing to medical overuse

Evidence to support inclusion

The test is identified as potentially contributing to medical overuse in certain populations by professionally recognised and EBM recommendations that aim to prevent medical overuse (e.g. Choosing Wisely, NICE 'Do Not Do' Recommendations, other recommendations against testing), as tests clinicians and consumers should question or avoid

Search & inclusion strategy:

1. Search terms for each test: "Do Not Do" OR "Choosing Wisely" AND recommend AND (*analyte and/or testing method - see column 1*) OR (*medical condition or test purpose - see column 1)*
2. Manual search of other known recommendations for and against screening (e.g. RACGP Guidelines for preventative activities in general practice)
3. The first 30 records were screened for relevance. We prefaced Australian and Australasian professional organisations first, and then international professional organisations. The country of origin of the recommendations/guidelines is reported in Column 2.

**Medical condition or test purpose; analyte and/or testing method**	**Reason for inclusion & Type of supporting evidence**	**Recommendation or summary of evidence**	**Reference**
Alzheimer's disease (Apolipoprotein E genotype)	Consumers advised to question the test by a Choosing Wisely Recommendation	Don't undertake genetic testing for methylenetetrahydrofolate reductase (MTHFR), apolipoprotein E (APOE) and other such tests where the clinical utility for	Choosing Wisely Recommendations, Human Genetics Society of Australasia (2022)

For peer review only - http://bmjopen.bmj.com/site/about/guidelines.xhtml

	(Australia)	diagnostic purposes is extremely low.	https://www.choosingwisely.org.au/recommendations/hgsa3. Accessed 23 May 2022.
Arthritis (Rheumatoid Factor)	Consumers advised to question the test by a Choosing Wisely Recommendation (United States (US))	Do not order rheumatoid factor (RF) alone, or as part of a 'panel' or 'cascade' in children to evaluate for rheumatologic disease such as juvenile idiopathic arthritis. due to musculoskeletal complaints. Do not let laboratory results guide referral.<br>The rheumatoid factor test is commonly used to help diagnose rheumatoid arthritis. However, a positive RF test does not always mean a person has rheumatoid arthritis, as there are several conditions that give positive rheumatoid factor results. Healthy people without rheumatoid arthritis can also test positive for rheumatoid factor, particularly older people. This does not mean they will develop the condition.	Choosing Wisely Recommendations, American Academy of Paediatrics (2022)<br>https://www.choosingwisely.org/clinician-lists/aap-sorh-rheumatoid-factor-to-evaluate-rheumatologic-disease/. Accessed 18 June 2022.<br>Arthritis Australia (2022)<br>https://arthritisaustralia.com.au/managing-arthritis/medical-management/blood-test-for-arthritis/. Accessed 18 June 2022.
Autoimmune disease (HLA-B27 antibody)	Consumers advised to question the test by a Choosing Wisely Recommendation (Canada)	Don't order an HLA-B27 unless spondyloarthritis is suspected based on specific signs or symptoms. HLA-B27 testing is not useful as a single diagnostic test in a patient with low back pain without further spondyloarthropathy, signs or symptoms because the diagnosis of spondyloarthropathy in these patients is of low probability. There is no clinical utility to ordering an HLA-B27 in the absence of positive imaging or the minimally required SpA signs or symptoms.	Choosing Wisely Recommendations, Canadian Rheumatology Association (2022) https://choosingwiselycanada.org/recommendation/rheumatology/. Accessed 18 June 2022.
Cancer markers (Carcino-embryonic antigen (CEA))	Consumers advised to question the test by a Choosing Wisely Recommendation (Australia)	Do not perform serum tumour marker tests except to evaluate or monitor a cancer known to produce these markers. In patients with non-specific symptoms, testing for a panel of tumour markers to try and diagnose an underlying cancer is not supported by evidence given the low sensitivity and specificity of these tests.	Choosing Wisely Recommendations, Medical Oncology Group of Australia (2022) https://www.choosingwisely.org.au/recommendations/moga4#. Accessed 23 May 2022.
Cancer risk factors (Glutathione S-transferases Genes test)	Consumers advised to question the test by a Choosing Wisely Recommendation (Australia)	Don't undertake genetic testing when clinical diagnostic criteria exist and there are no reproductive or predictive testing implications. Genetic tests do not have inherent utility. It is the adoption of therapeutic or preventive interventions that influences health outcomes.	Choosing Wisely Recommendations, Human Genetics Society of Australasia (2022) https://www.choosingwisely.org.au/recommendations/hgsa5. Accessed 25 August 2022.
Cardiovascular disease risk factors (Homocysteine test)	Consumers advised to question the test by a Choosing Wisely Recommendation (Australia)	Serum homocysteine as a screening test not recommended for low-risk populations. Its value as a risk factor for coronary heart disease is uncertain, and published RCTs show no evidence of benefit by lowering levels.	RCAGP Guidelines for preventative activities in general practice (2022) https://www.racgp.org.au/getattachment/1ad1a26f-9c8b-4e3c-b45b-3237272b3a04/Guidelines-for-preventive-activities-in-general-practice.aspx. Accessed 12 May

For peer review only - http://bmjopen.bmj.com/site/about/guidelines.xhtml

			2022.
Cardiovascular disease risk factors (MTHFR & APOE genetic test)	Consumers advised to question the test by a Choosing Wisely Recommendation (Australia)	Don't undertake genetic testing for methylenetetrahydrofolate reductase (MTHFR), apolipoprotein E (APOE) and other such tests where the clinical utility for diagnostic purposes is extremely low.	Choosing Wisely Recommendations, Human Genetics Society of Australasia (2022) https://www.choosingwisely.org.au/recommendations/hgsa3. Accessed 23 May 2022.
Coeliac disease (HLA gene typing)	Consumers advised to question the test by a Choosing Wisely Recommendation (Australia)	Do not undertake genetic testing for coeliac genes as a screening test for coeliac disease." The value of testing for coeliac genes is primarily as a negative test – if the gene test is negative then coeliac disease may be excluded. However as a coeliac gene can be found in approximately one third of the population, a positive result does not make coeliac disease a certainty. Serological testing, in a patient consuming an appropriate amount of gluten, is the appropriate first line screening test for coeliac disease. A small bowel biopsy is then required if serology is positive.	Choosing Wisely Recommendation, Gastroenterological Society of Australia (2022) https://www.choosingwisely.org.au/recommendations/gesa4#. Accessed 23 May 2022.
Food allergy (IgE)	Consumers advised to question the test by a Choosing Wisely Recommendation (Australia)	Food specific IgE testing should not be performed without a clinical history suggestive of IgE-mediated food allergy. While specific Immunoglobulin E (IgE) antibodies can be identified for a wide range of target allergens, the clinical significance of such tests is critically dependent on history. Since specific IgE antibodies are identifiable in many individuals with no evidence of allergic disease, the predictive value of these tests is strongly dependent on a history suggestive of underlying atopic disease with symptoms for which the detected IgE specificity is a plausible trigger.	Choosing Wisely Recommendation, Australasian Society of Clinical Immunology and Allergy (2022) https://www.choosingwisely.org.au/recommendations/ascia4. Accessed 12 May 2022. Australasian Society of Clinical Immunology and Allergy (2022) Laboratory Investigation of Allergic Diseases, https://www.allergy.org.au/hp/papers/tests-in-the-diagnosis-of-allergic-diseases. Accessed 12 May 2022.
Genital Herpes (antibody)	Consumers advised to question the test by a Choosing Wisely Recommendation (Australia)	Do not order herpes serology tests unless there is a clear clinical indication. Herpes serology is not an appropriate screening test in asymptomatic patients and does not accurately confirm whether the person is infected or is a transmission risk to others from asymptomatic shedding. Clinicians also need to consider whether test results will influence treatment or outcomes because, if they do not, then testing is a waste of finite health resources and is not indicated. Herpes serology tests only have good sensitivity and specificity in high prevalence populations. However, selective use of herpes serological tests may be justified for particular groups, such as those at high risk for STIs and human immunodeficiency virus (HIV) infection who are motivated to reduce their sexual risk behaviour; HIV-infected	Choosing Wisely Recommendations, Australasian Chapter of Sexual Health Medicine (2022) https://www.choosingwisely.org.au/recommendations/achshm1. Accessed 25 August 2022

For peer review only - http://bmjopen.bmj.com/site/about/guidelines.xhtml

		patients; patients with sexual partners with genital herpes; and in cases where a woman appears to have a first episode of herpes simplex virus (HSV) during pregnancy.	
Haemochromatosis (HFE gene mutation)	Consumers are advised to question the test by a Choosing Wisely Recommendation (US)	Don't order HFE genetic testing for a patient without iron overload or a family history of HFE-associated hereditary hemochromatosis. The majority of hereditary hemochromatosis is due to inheritance of HFE gene mutations. HFE gene mutations are common among individuals of European ancestry; however, only a small proportion of individuals with these mutations develop clinical disease. Other genetic and non-genetic factors contribute to disease expression. HFE genotyping should only be performed among individuals with iron overload or a known family history of HFE-associated hereditary hemochromatosis.	Choosing Wisely Recommendations, American College of Medical Genetics (2022) https://www.choosingwisely.org/clinician-lists/american-college-medical-genetics-genomics-hfe-genetic-testing/. Accessed 1 June 2022.
Heavy metal exposure (Heavy metal in blood)	Consumers are advised to question the test by a Choosing Wisely Recommendation (US)	Don't order heavy metal screening tests to assess non-specific symptoms in the absence of excessive exposure to metals. Individuals are constantly exposed to metals in the environment and often have detectable levels without being poisoned. Indiscriminate testing leads to needless concern when a test returns outside of a 'normal' range. Diagnosis of any metal poisoning requires an appropriate exposure history and clinical findings consistent with poisoning by that metal. A patient should only undergo specific metal testing if there is concern for a specific poisoning based on history and physical examination findings.	Choosing Wisely Recommendations, American College of Medical Toxicology and The American Academy of Clinical Toxicology (2022) https://www.choosingwisely.org/clinician-lists/american-college-academy-medical-toxicology-heavy-metal-screening-tests/. Accessed 10 October 2022
Immune disorders (Immunogenetics)	Consumers advised to question the test by a Choosing Wisely Recommendation (Australia)	Don't undertake genetic testing when clinical diagnostic criteria exist and there are no reproductive or predictive testing implications. Genetic tests do not have inherent utility. It is the adoption of therapeutic or preventive interventions that influences health outcomes.	Choosing Wisely Recommendations, Human Genetics Society of Australasia (2022) https://www.choosingwisely.org.au/recommendations/hgsa5. Accessed 25 August 2022
Insulin resistance (fasting glucose, fasting Insulin)	Consumers advised to question the test by a Choosing Wisely Recommendation (Australia)	Do not measure insulin concentration in the fasting state or during an oral glucose tolerance test to assess insulin sensitivity. Measurement of insulin either in the fasting state or during an oral glucose tolerance test is not a clinically useful method (and may be costly because of the insulin assay) to estimate insulin sensitivity.	Choosing Wisely Recommendations, The Endocrine Society of Australia (2022) https://www.choosingwisely.org.au/recommendations/esa3. Accessed 25 August 2022
Iron deficiency (Iron studies)	The test is not recommended by	Iron studies or serum iron should not be requested to diagnose iron deficiency:	Royal Children's Hospital Melbourne (2022) Clinical

For peer review only - http://bmjopen.bmj.com/site/about/guidelines.xhtml

	professional practice guidelines (Australia)	Serum iron reflects recent iron intake and does not provide a measure of the iron stores; Serum ferritin is an acute phase reactant and a normal result does not exclude iron deficiency in the presence of coexisting infection, inflammation or liver disease.	Practice Guidelines – Iron deficiency https://www.rch.org.au/clinicalguide/guideline_index/Iron_deficiency/. Accessed 11 October 2022.
Low-grade Inflammation (high sensitivity C-reactive protein (hsCRP))	Professional guidelines recommend against screening for this condition in certain populations (Australasia)	hsCRP test is a screening test not recommended by the RACGP Guideline for preventative practice, as there is insufficient evidence to support the role of hsCRP in preventive screening of asymptomatic patients.	RACGP (2021) Guidelines for preventive activities in general practice https://www.racgp.org.au/clinical-resources/clinical-guidelines/key-racgp-guidelines/view-all-racgp-guidelines/guidelines-for-preventive-activities-in-general-pr/communicable-diseases/sexually-transmissible-infections. Accessed 12 May 2022.
Neuro genetic disorders (Neurogenetic test)	Consumers advised to question the test by a Choosing Wisely Recommendation (Australia)	Don't undertake genetic testing when clinical diagnostic criteria exist and there are no reproductive or predictive testing implications. Like other screening or diagnostic tests, genetic tests do not have inherent utility. It is the adoption of therapeutic or preventive interventions that influences health outcomes. If clinical diagnostic criteria already exist for the condition in question and there are no reproductive or other predictive testing implications as a result of definitively identifying a genetic cause for the condition, then this renders genetic testing unnecessary.	Choosing Wisely Recommendations, Human Genetics Society of Australasia https://www.choosingwisely.org.au/recommendations/hgsa5. Accessed 25 August 2022
Prostate cancer (Prostate Specific Antigen (PSA))	Professional guidelines recommend against screening for this condition in certain populations (Australasia)	Do not perform PSA testing for prostate cancer screening in men with no symptoms and whose life expectancy is less than 7 years. Screening of asymptomatic (low-risk) men for prostate cancer by PSA testing is not recommended because the benefits have not clearly been shown to outweigh the harms. Pre-test discussion to address the benefits and harms (from overdiagnosis and overtreatment) of prostate cancer screening is recommended.	RACGP Guidelines for Preventative Activities in General Practice (2022) https://www.racgp.org.au/clinical-resources/clinical-guidelines/key-racgp-guidelines/view-all-racgp-guidelines/guidelines-for-preventive-activities-in-general-pr. Accessed 12 May 2022.
Thyroid disease (Thyroid hormones)	Consumers advised to question the test by a Choosing Wisely Recommendation (Australia)	Do not test thyroid function as population screening for asymptomatic patients. Clear evidence on the benefits and harms of screening is unavailable, and recommended against population-based screening. In the absence of evidence that early treatment reduces symptoms, lipid levels, or the risk of cardiovascular disease in patients with mild thyroid dysfunction detected by screening,	Choosing Wisely Recommendations, RACGP https://www.choosingwisely.org.au/recommendations/racgp10. Accessed 21 May 2022. RACGP Guidelines for Preventative Activities in General Practice (2022) https://www.racgp.org.au/clini

For peer review only - http://bmjopen.bmj.com/site/about/guidelines.xhtml

		screening for thyroid disease in asymptomatic populations is not recommended. This screening recommendation does not apply to people with symptoms suggestive of thyroid disease.	cal-resources/clinical-guidelines/key-racgp-guidelines/view-all-racgp-guidelines/guidelines-for-preventive-activities-in-general-pr. Accessed 12 May 2022.
Urinary tract infection (urine culture & microscopy)	Consumers advised to question the test by a Choosing Wisely Recommendation (US)	Don't obtain a urine culture unless there are clear signs and symptoms that localise to the urinary tract.	American Family Physician Choosing Wisely Recommendations (2022) https://www.aafp.org/afp/recommendations/viewRecommendation.htm?recommendationId=96. Accessed 21 May 2022.
Vitamin B deficiency (Vitamin B12, B6, B9)	Consumers advised to question the test by a NICE 'Do Not Do' Recommendation (United Kingdom (UK))	Tests for vitamin B12 deficiency & folate (B6) levels should not be carried out unless a full blood count and mean cell volume show a macrocytosis. Vitamin testing should be limited to patients with high-risk for deficiency, e.g. malabsorption or malnutrition	NICE 'Do Not Do Recommendation' (2022) https://www.nice.org.uk/donotdo/tests-for-vitamin-b12-deficiency-should-not-be-carried-out-unless-a-full-blood-count-and-mean-cell-volume-show-a-macrocytosis & https://www.nice.org.uk/donotdo/tests-for-folate-levels-should-not-be-carried-out-unless-a-full-blood-count-and-mean-cell-volume-show-a-macrocytosis. Accessed 31 May 2022.
Vitamin D deficiency (Vitamin D level)	Consumers advised to question the test by a Choosing Wisely Recommendation (Australia)	Do not perform population-based screening for Vitamin D deficiency; Clinicians should be aware that current vitamin D assay methods, may give different results and should understand the limitations of the method used in the laboratory that most of their patients attend.	Choosing Wisely Recommendation, Royal College of Pathologists Australasia (2022) https://www.choosingwisely.org.au/recommendations/rcpa3. Accessed 31 May 2022.
Vitamin K deficiency (Vitamin K level)	Consumers advised to question the test by a Choosing Wisely Recommendation (US)	Vitamin K deficiency is very rare. Measurements of the level of vitamin K in the blood are rarely used to determine if a deficiency exists. A diagnosis of Vitamin K deficiency is typically made by observing the prothrombin time correction following administration of vitamin K, plus the presence of clinical risk factors for vitamin K deficiency.	Choosing Wisely Recommendation, American Family Physician (2022) https://www.aafp.org/afp/recommendations/viewRecommendation.htm?recommendationId=244. Accessed 12 May 2022.

3. Category 2 Subgroup 2B: Tests with limited clinical utility - method has limited accuracy

Evidence to support inclusion

There is credible evidence to suggest the test or testing method has limited accuracy when used on its own

Search & inclusion strategy

For peer review only - http://bmjopen.bmj.com/site/about/guidelines.xhtml

1. PubMed and Google Advanced: (*"analyte and/or test method – see column 1")* AND diagnos* AND accuracy OR sensitivity OR specificity.
2. The first 30 records in each database were screened for relevance. i) We first prefaced EBM guidelines issued by Australian, Australasian and international professional or governmental organisations. The country or region of origin of the recommendations/guidelines is reported in Column 2. ii) If none were included, we included the peer-reviewed publication with the highest level of evidence (using Oxford Centre for Evidence-Based Medicine (CEBM): Levels of Evidence Grading[i]) were included. The type of evidence and CEBM grade level used is reported in Column 2. iii) If none were included, we then prefaced web-based consumer information issued by recognised expert health information organisations). A second or third publication may be included if the first publication included is insufficient to support the inclusion criteria.

**Medical condition or test purpose; analyte and/or testing method**	**Reason for inclusion & Type of supporting evidence**	**Recommendation or summary of evidence**	**Reference**
Blood clotting (Prothrombin Time)	Test has limited sensitivity or specificity (Peer-reviewed literature, CEBM Grade Level 1 – Systematic review)	The prothrombin time (PT) test measures the time it takes for blood to form a clot. It is usually used for monitoring patients already under clinical care, e.g. using warfarin and other Vitamin K antagonists to monitor treatment and adjust doses, and for preoperative testing. PT is limited by biological variability, insensitivity to other bleeding disorders, variability in results due to differences in reagents and coagulation. Routine preoperative haemostasis testing with PT is not useful in asymptomatic patients with no known risk factors. High rate of false-positive and false-negative results may lead to inappropriate precautionary measures or false reassurance.	Levy, J. H., Szlam, F., Wolberg, A. S., & Winkler, A. (2014). Clinical use of the activated partial thromboplastin time and prothrombin time for screening: a review of the literature and current guidelines for testing. Clinics in laboratory medicine, 34(3), 453-477.
Chronic fatigue (Iron & Vitamin B12, thyroid hormones)	Test method has limited accuracy when used on its own (Consumer information issued by recognised expert organisation)	Iron and vitamin B12 and thyroid hormones are important biomarkers of chronic fatigue and may play a role in treatment, diagnosis requires comprehensive assessment. Abnormal iron, Vitamin B12 and thyroid hormone levels may be an indicator of a range of other conditions, such as anaemia and thyroid disease.	Health Direct (2022) Chronic fatigue syndrome, https://www.healthdirect.gov.au/chronic-fatigue-syndrome-cfs. Accessed 12 May 2022
Coeliac disease (IgA, IgG antibody)	Test method has limited accuracy when used on its own (Peer-reviewed literature, CEBM Grade Level 5 – Expert opinion based on 'first principles')	Coeliac disease is an immune reaction to eating gluten, and creates inflammation that damages the small intestine's lining. Blood tests that check for antibodies (IgA & IgG) is typically the first step in diagnosing celiac disease, however, serological investigation alone is insufficient to make the diagnosis. A definitive diagnosis of coeliac disease	Lewis, D., Haridy, J., & Newnham, E. D. (2017). Testing for coeliac disease. *Australian prescriber*, *40*(3), 105

For peer review only - http://bmjopen.bmj.com/site/about/guidelines.xhtml

		requires gastroscopy and duodenal biopsy. Up to 5% of patients with coeliac disease can have negative serology.	
Gastrointestinal candida (IgG, IgM antibody)	Test method has limited accuracy when used on its own (Peer-reviewed literature, CEBM Grade Level 2 – level 2 evidence synthesis)	The candida fungus is part of the normal gastrointestinal track of healthy populations, and its presence is not sufficient to produce clinically significant candidiasis infection. The standard diagnostic test for gastrointestinal candidiasis is a blood culture. Antibody tests are nonculture diagnostic tests are non-invasive and have the potential to identify patients with currently unrecognised candidiasis and shorten the time to diagnosis. However, no nonculture *Candida* diagnostic tests have been shown to reduce mortality and morbidity, shorten hospital stays, or restrain the emergence of antifungal resistance.	Clancy, C. J., & Nguyen, M. H. (2018). Diagnosing invasive candidiasis. *Journal of Clinical Microbiology*, *56*(5), e01909-17.
Giardia (Rapid antigen test)	Test method has limited accuracy when used on its own (Peer-reviewed literature, CEBM Grade Level 3 – level 3 evidence synthesis)	Several immunodiagnostic tests of rapid diagnosis of giardiasis have been developed in decades, mainly based on the detection of Giardia antigens in faecal specimens. While many of these methods have high sensitivity, they should only serve as a complementary test to the gold standard traditional microscopy stool examination performed in the routine medical laboratory.	Hooshyar, H., Rostamkhani, P., Arbabi, M., & Delavari, M. (2019). Giardia lamblia infection: review of current diagnostic strategies. *Gastroenterology and hepatology from bed to bench*, *12*(1), 3–12.
Helicobacter pylori (Stool antigen)	Test method has limited accuracy when used on its own (Peer-reviewed literature, CEBM Grade Level 1 - Systematic review)	In people with no history of gastrectomy and those who have not recently had antibiotics or proton pump inhibitors, urea breath tests had high diagnostic accuracy while serology and stool antigen tests had lower accuracy to detect H pylori infection (Best et al, 2018).	Best, L. M., Takwoingi, Y., Siddique, S., Selladurai, A., Gandhi, A., Low, B., ... & Gurusamy, K. S. (2018). Non-invasive diagnostic tests for Helicobacter pylori infection. *Cochrane Database of Systematic Reviews*, (3).
Inflammatory bowel disease (Faecal calprotectin test)	The test should only be used based on assessing symptoms and medical history (Consumer information issued by recognised expert organisation and government organisation)	The faecal calprotectin test is used to distinguish between Inflammatory bowel disease (IBD) and irritable bowel syndrome (IBS), which is a functional condition of altered bowel habits with no inflammation. As a screening tool, faecal calprotectin tests reduce the need for invasive diagnostic procedures (such as colonoscopy), potentially leading to earlier diagnosis of IBD and IBS and subsequently improving patient management. However, these tests should only be limited to those necessary, based on symptoms and medical history.	Australian Government, Medical Services Advisory Committee (2022) Medical Measurement of Calprotectin as a marker of bowel inflammation http://www.msac.gov.au/internet/msac/publishing.nsf/Content/1353-public. Accessed 13 October 2022
Kidney disease (Creatinine	Test method has limited accuracy when used on its	Urine dipstick is an inexpensive, non-invasive and accessible test for	White, S. L., Yu, R., Craig, J. C., Polkinghorne, K. R., Atkins, R.

For peer review only - http://bmjopen.bmj.com/site/about/guidelines.xhtml

Albumin Ratio)	own (Peer-reviewed literature, CEBM Grade Level 3 – Cross sectional cohort study)	measuring albumin concentration in urine for the purpose of detecting kidney disease. However, this method has high false-positive rates, emphasising the need for laboratory confirmation of positive results. Laboratory Creatinine/Albumin Ratio is a recommended screening test for kidney disease, and is useful in managing diabetes and assessing the risk and progression of diabetic nephropathy. However, the tests should only be limited to those necessary, based on symptoms and medical history.	C., & Chadban, S. J. (2011). Diagnostic accuracy of urine dipsticks for detection of albuminuria in the general community. American Journal of Kidney Diseases, 58(1), 19-28.
Liver function (Bilirubin dipstick)	Test method has limited accuracy when used on its own (Peer-reviewed literature, CEBM Grade Level 4 - Retrospective review)	A urine dipstick measuring urine-bilirubin is a common and non-invasive measure of bilirubin, however, it is known to yield a high rate of false-positive results. In patients with an unexpected positive urine bilirubin test result, 85% had abnormal liver function test results after their positive urine bilirubin result. However, unexpected positive results amounted to only 0.13% of all test results. Urine bilirubin does not appear to add significant information toward the diagnosis of liver disease in most patients.	Foley, K. F., & Wasserman, J. (2014). Are unexpected positive dipstick urine bilirubin results clinically significant? A retrospective review. *Laboratory Medicine*, *45*(1), 59-61.
Measles, mumps, rubella serology (Antibodies)	Test method has limited accuracy when used on its own (Guideline issued by recognised professional organisation)	Routine serological testing for varicella does not provide a reliable measure of vaccine-induced immunity. Although the test can indicate whether natural immunity has occurred due to prior infection, it does not reliably guide diagnosis of clinically significant disease.	RACGP Guidelines for Preventative Activities in General Practice (2022) https://www.racgp.org.au/clinical-resources/clinical-guidelines/key-racgp-guidelines/view-all-racgp-guidelines/guidelines-for-preventive-activities-in-general-pr . Accessed 13 October 2022
Pancreatic insufficiency (pancreatic elastase 1)	Test method has limited accuracy when used on its own (Guideline issued by recognised professional organisation)	Pancreatic insufficiency is the inability of the pancreas to produce and/or transport enough digestive enzymes to break down food in the intestine and allow its absorption. The pancreatic elastase 1: test on its own may lead to false positives or misattribution of diagnosis.	Nikfarjam, M., Wilson, J. S., & Smith, R. C. (2017). Diagnosis and management of pancreatic exocrine insufficiency: Australasian Pancreatic Club Pancreatic Enzyme Replacement Therapy Guidelines Working Group. *Medical Journal of Australia*, *207*(4), 161-165.
Pancreatitis (lipase, amylase)	Test method has limited accuracy when used on its own (Peer-reviewed literature, CEBM Grade Level 3 – level 3 evidence synthesis)	Blood test for amylase and lipase rise within hours of an episode of acute pancreatitis are key components of the diagnostic criteria of acute pancreatitis. However, radiological findings are needed to confirm a diagnosis. Other conditions can also cause slightly	Basnayake C, Ratnam D. (2015) Blood tests for acute pancreatitis. Aust Prescr 2015;38:128-30.https://doi.org/10.18773/austprescr.043

For peer review only - http://bmjopen.bmj.com/site/about/guidelines.xhtml

		increased lipase levels. Using the test on its own may lead to false positives or misattribution of diagnosis.	
Urinary tract infection (Nitrite and leukocyte esterase dipstick)	The test method is not reliable on its own as a diagnostic test (Peer-reviewed literature, CEBM Grade Level 5 – Expert opinion based on 'first principles')	Urinary tract infections (UTI) or cystitis is a common bacterial infection. Nitrite and leukocyte esterase dipsticks are useful for ruling out and ruling in cystitis, but a positive urine culture with pyuria confirms the diagnosis.	Colgan R, Williams M. (2011) Diagnosis and treatment of acute uncomplicated cystitis. American Family Physician. 84(7):771-6.
Vaginal infection (pH)	The test has limited sensitivity or specificity (Peer-reviewed literature, CEBM Grade Level 3 – Cross sectional cohort study)	Vaginal bacterial infection is common among women of reproductive age. Vaginal pH determination with a swab is relatively sensitive, but less specific in detecting women with bacterial infection. The pH test may be suitable for home screening and direct patients towards the use of over-the-counter (OTC) antifungal medication, however, complicated vaginal infections should be diagnosed by healthcare providers.	Hemalatha, R., Ramalaxmi, B. A., Swetha, E., Balakrishna, N., & Mastromarino, P. (2013). Evaluation of vaginal pH for detection of bacterial vaginosis. *The Indian journal of medical research*, *138*(3), 354.

4. Category 2, Subgroup 2C: Tests with limited clinical utility – condition tested for has low pre-test probability

Evidence to support inclusion (2C)

There is credible evidence to suggests the condition tested for has low pre-test probability among the healthy general population.

Search & inclusion strategy

1. For each test: PubMed (with MeSH terms) and Google Advanced: (*"medical condition or test purpose" – see column 1)* OR (*"testing method and/or analyte" – see column 1*) AND Australia OR prevalence OR diagnos* OR test* OR screen*
2. The first 30 records in each database were screened for relevance. i) We first prefaced EBM guidelines issued by Australian, Australasian and international professional or governmental organisations. The country or region of origin of the recommendations/guidelines is reported in Column 2. ii) If none were included, we included the peer-reviewed publication with the highest level of evidence (using CEBM Grading) were included. The type of evidence and CEBM grade level used is reported in Column 2. iii) If none were included, we then prefaced web-based consumer information issued by recognised expert health information organisations).

Medical condition or test purpose; analyte and/or testing method	Reason for inclusion & Type of supporting evidence	Recommendation or summary of evidence	Reference
Cytomegalovirus (CMV) (IgC & IgM)	The test is for a condition with low pre-test probability in the general population (Consumer information issued by recognised health information organisation)	By the age of 20 years, around 50% of adults would have been infected with CMV, but it rarely causes symptoms. Most people with CMV infection may have a self-limiting flu-like or glandular fever-like illness. CMV tests are only needed to help diagnose a current,	MedlinePlus (2022) Cytomegalovirus (CMV) Tests, https://medlineplus.gov/lab-tests/cytomegalovirus-cmv-tests/ Accessed 31 May 2022

For peer review only - http://bmjopen.bmj.com/site/about/guidelines.xhtml

		reactivated, or past CMV infection in people at risk for health complications.	
Epstein-Barr virus (EBV) (EBV antibody)	The test is for a condition with low pre-test probability in the general population (Consumer information issued by recognised professional organisation & government organisation)	Approximately 95% of the world's adult population have been infected by EBV, but it rarely causes symptoms. An EBV antibody test may be needed in non-typical cases of mononucleosis or if another illness caused by EBV infection is suspected, or used to investigate flu-like symptoms in pregnancy.	Pathology Tests Explained (2022) Epstein Barr Virus Antibodies https://pathologytestsexplained.org.au/learning/test-index/epstein-barr-virus-antibodies. Accessed 21 May 2022<br>WebMD (2022) What to Know About the Epstein-Barr Virus Test https://www.webmd.com/a-to-z-guides/what-to-know-epstein-barr-virus-test. Accessed 21 May 2022
Glucose-6-Phosphate-Dehydrogenase deficiency (G6PD) (G6PD enzyme test)	The test is for a condition with low pre-test probability in the general population (Consumer information issued by recognised professional organisation, international)	G6PD deficiency is one of the most common forms of enzyme deficiency, affect more than 400 million people worldwide. However, the vast majority of people remain clinically asymptomatic throughout their lives. G6PD deficiency does not seem to affect life expectancy, quality of life, or the activity of affected individuals. Testing for G6PD deficiency should be considered when an acute haemolytic reaction triggered by exposure to a known oxidative drug, infection, or ingestion of fava beans; and prior to commencing treatment with certain medications such as primaquine, tafenoquine and rasburicase. It is not a routine test. Screening would only be justified for newborns in families with disease and in populations with higher risk for malaria.	Rare Diseases (2022) https://rarediseases.org/rare-diseases/glucose-6-phosphate-dehydrogenase-deficiency/. Accessed 21 October 2022
Lyme disease (Borrelia antibody)	The test is for a condition with low pre-test probability in the general population (Peer-reviewed literature, CEBM Grade Level 3 – level 3 evidence synthesis)	Lyme disease is caused by bacteria transmitted in a tick bite. The usefulness of the serological tests for Lyme disease depends on the pre-test probability and subsequent predictive values in the setting where the tests are being used. The types of tick that carry the bacteria are not native to Australia therefore it is unlikely to transmit Lyme disease. Treating false positive results, or treating patients with "Lyme disease-like illness" can lead to prolonged antibiotic therapy that is unjustified, and is likely to do much more harm than good.	Collignon, P. J., Lum, G. D., & Robson, J. M. (2016). Does Lyme disease exist in Australia? *Medical Journal of Australia*, *205*(9), 413-417.
Rotavirus (Rapid antigen test)	The test is for a condition with low pre-test probability in the general population (Consumer information	Rotavirus is an infectious disease most commonly among children under 5. The symptoms are diarrhoea, vomiting and stomach pains, and can cause	The Australian Government Department of Health (2022) Rotavirus Laboratory Case Definition (LCD)

For peer review only - http://bmjopen.bmj.com/site/about/guidelines.xhtml

	issued by government organisation)	severe gastroenteritis. There is no treatment for Rotavirus, but severe symptoms require hospitalisation. Commercially available rapid antigen detections kits are based on the VP6 antigen of group A rotaviruses, which means this is the only rotavirus serogroup detected. Recent experience in Australia has found a high false-positive rate for antigen detection tests compared with PCR, especially in vaccinated children. Confirmation of positive results should be done where there is a low pre-test probability that the person has rotavirus infection.	https://www1.health.gov.au/internet/main/publishing.nsf/Content/cda-phlncd-rotavirus.htm. Accessed 28/5/2022.

5. Category 3: Commercial 'health checks' with low potential clinical utility

Evidence to support inclusion

1. There is insufficient evidence to support clinical utility of the test as a routine assessment of health for low-risk and asymptomatic general practice populations; OR
2. There is literature to suggest the lack of clinical validity as a testing method for the named medical condition.

Search & inclusion strategy

1. For each test: a Google Advanced search using key words: (*"medical condition or test purpose - see column* 1) OR (*"analyte and/or testing method – see column 1"*) AND test* OR Recommend* OR Guide*. The first 30 records were screened for relevance. If no relevant sources were included, then PubMed (with MeSH terms): *(test or analyte – reported in column 1*) AND utility OR test* OR screen*
2. The first 30 records in each database were screened for relevance. i) We first prefaced EBM guidelines issued by Australian, Australasian and international professional or governmental organisations. The country or region of origin of the recommendations/guidelines is reported in Column 2. ii) If none were included, we included the peer-reviewed publication with the highest level of evidence (using CEBM Grading) were included. The type of evidence and CEBM grade level used is reported in Column 2. iii) If none were included, we then prefaced web-based consumer information issued by recognised expert health information organisations).

**Medical condition or test purpose; analyte and/or testing method**	**Reason for inclusion & Type of supporting evidence**	**Recommendation or summary of evidence**	**Reference**
Biological age (Glycans)	Insufficient evidence to support clinical utility of the test as an indicator of health status among healthy populations (Peer-reviewed literature, CEBM Grade Level 3 – level 3 evidence synthesis)	There is insufficient evidence to demonstrate clinical utility of measuring 'biological age', which is a hypothetical measure of the biological aging process, as opposed to chronological age as a marker of passage of time. There is still limited understanding about the mechanisms by which biological age predictors work. It remains unclear how best to	Jylhävä, J., Pedersen, N. L., & Hägg, S. (2017). Biological age predictors. EBioMedicine, 21, 29-36.<br>Nwanaji-Enwerem, J. C., & Mair, W. B. (2022). Redefining age-based screening and diagnostic guidelines: an opportunity for biological aging clocks in clinical medicine?. The Lancet Healthy

For peer review only - http://bmjopen.bmj.com/site/about/guidelines.xhtml

		incorporate measures of biological age into clinical medicine, and there is a need for trials that show modifying epigenetic age acceleration impacts human morbidity and mortality.	Longevity, 3(6), e376-e377.\
Bone health (serum alkaline phosphatase, calcium, potassium)	Insufficient evidence to support clinical utility of the test as an indicator of health status among healthy populations (Consumer information issued by recognised professional organisation, Australia)	An elevated level of bone markers may be seen in conditions such as osteoporosis or Paget disease. However, bone marker testing is typically only indicated in people who have been diagnosed with or are at risk of bone loss. The tests are not intended to be used to screen the general public.	Pathology Tests Explained (2022) Bone Markers, https://pathologytestsexplained.org.au/learning/test-index/bone-markers. Accessed 12 October 2022
Coenzyme Q10 (CoQ10) profile (Coenzyme Q10 level)	Insufficient evidence to support clinical utility of the test as an indicator of health status among healthy populations (Official information issued by government organisation, US)	CoQ10 has important functions in the body, people with some diseases may have reduced levels. However, low or high levels of CoQ10 do not necessarily indicate a problem in a person without symptoms of deficiency. Clinically significant coenzyme deficiency needs to be further diagnosed and not rely on blood plasma CoQ10 measures alone.	US Department of Health, National Centre for Complementary and Integrative Health (2022) Health Information – Coenzyme Q10 https://www.nccih.nih.gov/health/coenzyme-q10. Accessed 12 October 2022
Cytokine profile (Cytokine level)	Insufficient evidence to support clinical utility of the test as an indicator of health status among healthy populations (Guideline issued by recognised professional organisation, Australia)	Cytokines have been shown to regulate immunologic responses, hematopoietic development, and cell-to-cell communication as well as host responses to infectious agents and inflammatory stimuli. However, the clinical usefulness of measuring cytokine levels in plasma remains unclear. There is no definite diagnostic application for cytokine profile test at present.	RCPA Manual (2022) Cytokine Profile Test https://www.rcpa.edu.au/Manuals/RCPA-Manual/Pathology-Tests/C/Cytokine-profile-test. Accessed 6 October 2022
Fertility status - male (Semen analysis)	Insufficient evidence to support clinical utility of the test as an indicator of health status among healthy populations (Peer-reviewed literature, CEBM Grade Level 5 – Expert opinion based on 'first principles')	Semen analysis is subject to considerable interobserver variability and has been difficult to standardise, particularly with regard to the quantification of concentration and morphology. The results of semen analysis are often nuanced, making self-interpretation of results difficult without the guidance of a reproductive physician. Individual semen analysis parameters, even if abnormal, may be weak indicators of a man's fertility potential.	Stanczyk, F. Z., Mandelbaum, R. S., & Lobo, R. A. (2022). Potential pitfalls of reproductive direct-to-consumer testing. *F&S Reports*, *3*(1), 3-7.
Gastrointestinal function (Microbiome analysis)	Insufficient evidence to support clinical utility of the test as an indicator of health status among healthy populations (Guideline issued by recognised professional organisation,	The British Dieticians Association does not advise commercial gut microbiota testing and lifestyle change programs based on the test results. Research to date indicates there is no standard microbiome composition that can be used as a baseline for health and few	British Dieticians' Association, Commercial Gut Microbiome (2021) https://www.bda.uk.com/resource/commercial-gut-microbiome-testing.html. Accessed 11 July 2022.

For peer review only

For peer review only - http://bmjopen.bmj.com/site/about/guidelines.xhtml

	UK)	instances where there is a definitive association between any gut microbe and disease risk.	
Gastrointestinal function (Stool Analysis)	Insufficient evidence to support clinical utility of the test as an indicator of health status among healthy populations (Consumer information issued by recognised professional organisation, Australasia)	Laboratory analysis of stools can be useful , especially in gastroenterology. However, commercial stool analysis marketed as 'functional' assessment of gastrointestinal health may vary in protocol and the analysis, and do not have clinical utility for healthy populations.	Friends of Science In Medicine (FSM) Recommendations For Pathology Tests In Australia (2013) https://pathologytestsexplained.org.au/lto_au/media/images/FSM-pathologyrecommendations.pdf. Accessed 11 July 2022.
Health & Wellness profile (Panel consisting of tests for more than one of: full blood count, hormones, organ function, vitamins & minerals)	Insufficient evidence to support clinical utility of the test as an indicator of health status among healthy populations (Peer-reviewed literature, CEBM Grade Level 1 - Systematic review of level 1 studies)	General health checks are screening test for more than one disease or risk factor in more than one organ system in a person who does not feel ill. These aim to detect disease and risk factors for disease with the purpose of reducing morbidity and mortality. However, evidence does not suggest general health checks reduces morbidity or mortality, neither overall nor for cardiovascular or cancer causes, although the number of new diagnoses was increased. Important harmful outcomes, such as the number of follow-up diagnostic procedures or short-term psychological effects, were often not studied or reported and many trials had methodological problems.	Krogsbøll, L. T., Jørgensen, K. J., Larsen, C. G., & Gøtzsche, P. C. (2012). General health checks in adults for reducing morbidity and mortality from disease: Cochrane systematic review and meta-analysis. *BMJ*, *345*.
Hormone profile – female fertility status (Panel consisting of tests for more than one of: Follicle Stimulating Hormone, luteinising hormone, estradiol, progesterone, testosterone)	Insufficient evidence to support clinical utility of the test as an indicator of health status among healthy populations (Guideline issued by recognised professional organisation, Canada)	Tests for female reproductive hormone are used to investigate primary or secondary hypogonadism, infertility, prediction of ovulation, and for monitoring patients undergoing fertility treatment. The tests do not indicate the fertility status of healthy populations not under clinical care.	British Columbia Medical Association (2022) Hormone Testing – Indicators and Appropriate Use, https://www2.gov.bc.ca/gov/content/health/practitioner-professional-resources/bc-guidelines/special-endocrine-testing. Accessed 22 May 2022
Hormone profile – female health (Panel consisting of tests for more than one of: Follicle Stimulating Hormone, luteinising hormone, estradiol, progesterone,	Insufficient evidence to support clinical utility of the test as an indicator of health status among healthy populations (Guideline issued by recognised professional organisation, Canada)	Tests for female reproductive hormone are used to investigate primary or secondary hypogonadism, infertility, prediction of ovulation, and for monitoring patients undergoing fertility treatment. The tests do not indicate the general health status of healthy populations not under clinical care.	British Columbia Medical Association (2022) Hormone Testing – Indicators and Appropriate Use, https://www2.gov.bc.ca/gov/content/health/practitioner-professional-resources/bc-guidelines/special-endocrine-testing. Accessed 22 May 2022

For peer review only - http://bmjopen.bmj.com/site/about/guidelines.xhtml

testosterone)			
Hormone profile – general health (Panel consisting of tests for more than one of: adrenal, growth, thyroid & reproductive hormones)	Insufficient evidence to support clinical utility of the test as an indicator of health status among healthy populations (Guideline issued by recognised professional organisation, Canada)	Testosterone and luteinising hormone tests have indications for investigating primary or secondary hypogonadism. Sex hormone binding globulin (SHBG) is used to clarify borderline low total testosterone. Estradiaol, dehydroepiandrosterone (DHEA), androstenedione are not indicated in routine clinical practice. The tests do not indicate the general health status of healthy populations not under clinical care.	British Columbia Medical Association (2022) Hormone Testing – Indicators and Appropriate Use, https://www2.gov.bc.ca/gov/content/health/practitioner-professional-resources/bc-guidelines/special-endocrine-testing. Accessed 22 May 2022
Hormone profile – male health (Panel consisting of tests for more than one of: DHEA, estrone, estradiol, testosterone, SHBG, androstenedione)	Insufficient evidence to support clinical utility of the test as an indicator of health status among healthy populations (Guideline issued by recognised professional organisation, Canada)	Testosterone and luteinising hormone tests have indications for investigating primary or secondary hypogonadism. SHBG – clarification of borderline low total testosterone. Estradiaol, DHEA, androstenedione are not indicated in routine clinical practice. The tests do not indicate the general health status of healthy populations not under clinical care.	British Columbia Medical Association (2022) Hormone Testing – Indicators and Appropriate Use, https://www2.gov.bc.ca/gov/content/health/practitioner-professional-resources/bc-guidelines/special-endocrine-testing. Accessed 22 May 2022
Hormone profile – Menopause (Panel consisting of tests for more than one of: estradiol, estriol, estrone, DHEA)	Insufficient evidence to support clinical utility of the test as an indicator of health status among healthy populations (Consumer information issued by recognised professional organisation, US)	Testing hormone levels is not required to determine whether a woman has the "right amount" of hormones. The optimal hormone levels in postmenopausal women have not been established. How symptoms respond to a particular dose of hormones or non-hormonal menopause medication is the only reliable guide. Because hormone levels vary day to day as well as throughout the day, even a blood and saliva test cannot accurately reflect the body's hormone levels.	The North American Menopause Society (2022) What is hormone testing https://www.menopause.org/publications/clinical-practice-materials/bioidentical-hormone-therapy/what-is-hormone-testing-. Accessed 12 May 2022.
Hormone profile – ovarian reserve (Anti-Mullerian Hormone (AMH)	Insufficient evidence to support clinical utility of the test as an indicator of health status among healthy populations (Peer-reviewed literature, CEBM Grade Level 3 - cohort study)	AMH are used in the clinical setting for fertility treatments. In the general population and those not undergoing treatment for infertility, AMH does not predict future fertility potential, the likelihood of unassisted pregnancy, the time to pregnancy, or the timing of menopause.	Depmann M., Broer S.L., Eijkemans M.J.C., van Rooij I.A.J., Scheffer G.J., Heimensem J., et al. (2017) Anti-Müllerian hormone does not predict time to pregnancy: results of a prospective cohort study. Gynecol Endocrinol. ;33:644–648.
Hormone profile – sleep quality (Cortisol & Melatonin)	Insufficient evidence to support clinical utility of the test as an indicator of health status among healthy populations (Guideline issued by recognised professional organisation, Canada)	Cortisol (24-hour urine free) is used for targeted screening for Cushing Syndrome caused by endogenous cortisol excess only. Cortisol (serum/plasma) is not useful in the investigation of non-specific fatigue and lethargy unless accompanied by clinical syndromes of Addison's Disease or Cushing Syndrome.	British Columbia Medical Association (2022) Hormone Testing – Indicators and Appropriate Use, https://www2.gov.bc.ca/gov/content/health/practitioner-professional-resources/bc-guidelines/special-endocrine-

For peer review only - http://bmjopen.bmj.com/site/about/guidelines.xhtml

		Measurements of morning and afternoon cortisol (for diurnal variation) is not useful.	testing. Accessed 22 May 2022
Hormone profile – Sports & fitness profile (Growth & reproductive hormones)	Insufficient evidence to support clinical utility of the test as an indicator of health status among healthy populations (Guideline issued by recognised professional organisation, Canada)	Screening for growth hormone related disorders with insulin-like growth factor 1 (IGF1) is only indicated in patients demonstrating symptoms of growth hormone excess (acromegaly) or deficiency. The reference intervals are highly dependent on age. Tests for reproductive hormones are used to investigate primary or secondary hypogonadism and other endocrine disease or monitoring patients undergoing fertility treatment. Estradiaol, DHEA, androstenedione are not indicated in routine clinical practice. The tests do not indicate fitness or sports performance level in healthy populations.	British Columbia Medical Association (2022) Hormone Testing – Indicators and Appropriate Use, https://www2.gov.bc.ca/gov/content/health/practitioner-professional-resources/bc-guidelines/special-endocrine-testing. Accessed 22 May 2022.
Hormone profile – stress (salivary cortisol)	Insufficient evidence to support clinical utility of the test as an indicator of health status among healthy populations (Guideline issued by recognised professional organisation, Canada)	Salivary cortisol is used to investigate the cortisol response to stress in a research setting. In a clinical context, the utility of salivary hormone testing is limited to Cushing Syndrome screening using late night salivary cortisol.	Inder, W. J., Dimeski, G., & Russell, A. (2012). Measurement of salivary cortisol in 2012–laboratory techniques and clinical indications. *Clinical endocrinology*, *77*(5), 645-651.
Nutrigenomic profile (genetic)	Insufficient evidence to support clinical utility of the test as an indicator of health status among healthy populations (Guideline issued by recognised professional organisation, international)	The Consensus Report of the Academy of Nutrition and Dietetics suggest there is insufficient evidence regarding the effectiveness of incorporating nutrigenetic testing into nutrition counselling or care and reporting dietary or clinical outcomes at present. Research on the application of nutritional genomics to practice is in its infancy.	Braakhuis, A., Monnard, C. R., Ellis, A., & Rozga, M. (2021). Consensus report of the Academy of Nutrition and Dietetics: incorporating genetic testing into nutrition care. *Journal of the Academy of Nutrition and Dietetics*, *121*(3), 545-552.
Nutritional status (Amino acids)	Insufficient evidence to support clinical utility of the test as an indicator of health status among healthy populations (Guideline issued by recognised professional organisation, US.)	Assessing amino acid alone is not a reliable indication of nutritional status, because the reference range of amino acid concentrations vary with age. Interpretations of amino acid results are based upon relative amino acid levels, pattern recognition, and correlation of positive and negative findings, rather than on individual amino acids levels alone. Amino acid abnormalities or overall profiles should also be considered in the context of clinical findings and/or additional test results.	Sharer, J. D., De Biase, I., Matern, D., Young, S., Bennett, M. J., & Tolun, A. A. (2018). Laboratory analysis of amino acids, 2018 revision: a technical standard of the American College of Medical Genetics and Genomics (ACMG). *Genetics in Medicine*, *20*(12), 1499-1507.
Nutritional status (Ammonia)	Insufficient evidence to support clinical utility of the test as an indicator of health status among healthy	In healthy populations, there is a normal range of ammonia in the blood but this will vary depending on the age of a person. A test for ammonia is not	Tietze KJ. (2012) Review of Laboratory and Diagnostic Tests. In: Tietze KJ, ed. *Clinical Skills for Pharmacists (Third Edition).* Saint

For peer review only - http://bmjopen.bmj.com/site/about/guidelines.xhtml

	populations (Peer-reviewed literature, CEBM level 2 – level 2 evidence synthesis)	used as a routine screening test. It is used to diagnose and/or monitor conditions that cause high ammonia levels, such as liver disease, urea cycle disorders.	Louis: Mosby:86-122
Nutritional status (Chromium)	Insufficient evidence to support clinical utility of the test as an indicator of health status among healthy populations (Peer-reviewed literature,	Chromium deficiency has not been reported in healthy populations, and no definitive deficiency symptoms have been established. There is a lack of an accepted biomarker of chromium nutritional status.	Vincent JB, Lukaski HC. Chromium. (2018) Adv Nutr. Jul 1;9(4):505-506. doi: 10.1093/advances/nmx021. PMID: 30032219; PMCID: PMC6054252.
Nutritional status (Copper)	Insufficient evidence to support clinical utility of the test as an indicator of health status among healthy populations (Guideline issued by recognised professional organisation, Australasia)	Plasma copper is not a valid indicator of general health and nutritional status. The clinical purpose of a copper test is to diagnose Wilson's disease, Menkes disease and, occasionally, investigation of copper status in parenteral alimentation, and investigation of suspected copper toxicity.	RCPA Manual (2022) https://www.rcpa.edu.au/Manuals/RCPA-Manual/Pathology-Tests/C/Copper. Accessed 31 May 2022.
Nutritional Status (Essential Fatty Acids)	Insufficient evidence to support clinical utility as valid indicator of general health (Consumer information issued by recognised professional organisation)	Essential fatty acid testing or fatty acid profiling involves analysing specific types of fats in the blood stream. Commonly advertised are from fatty acids of the Omega-3 and Omega-6 groups.-Whilst there is good evidence that modifying diet to include more 'good' fats (i.e. omega-3) is beneficial, there is no evidence that testing fatty acids is able to provide any further information or health benefit.	LabTests Online UK (2022) https://labtestsonline.org.uk/tests/unvalidated-or-misleading-laboratory-tests. Accessed 31 May 2022
Nutritional status (Glutathione)	Insufficient evidence to support clinical utility as valid indicator of general health (Peer-reviewed literature, CEBM level 2 – level 2 evidence synthesis)	Measuring glutathione levels can help to determine if a person is deficient and may benefit from supplements or an increased diet of glutathione-rich foods. However, while optimizing glutathione levels has been proposed as a strategy for health promotion and disease prevention, clear, causal relationships between glutathione status and disease risk or treatment remain to be clarified.	Minich, D. M., & Brown, B. I. (2019) A review of dietary (phyto) nutrients for glutathione support. *Nutrients*, *11*(9), 2073.
Nutritional status (Iodine)	Insufficient evidence to prove clinical utility as valid indicator of general health (Peer-reviewed literature, CEBM level 3 – level 3 evidence synthesis)	There are limitations of assessing iodine status in the general population. Urine iodine is highly variable from day to day in a given patient and has low predictive value for iodine deficiency. Median urine iodine concentrations used in epidemiological studies should not be applied to individuals; if they are then iodine deficiency will be overdiagnosed. The need for multiple samples and a long sampling period severely limits the clinical utility of	Wainwright, P., & Cook, P. (2019). The assessment of iodine status–populations, individuals and limitations. *Annals of Clinical Biochemistry*, *56*(1), 7-14.

For peer review only - http://bmjopen.bmj.com/site/about/guidelines.xhtml

		such tests.	
Nutritional status (Magnesium test)	Insufficient evidence to prove clinical utility as valid indicator of general health (Peer-reviewed literature, CEBM level 3 – level 3 evidence synthesis)	There is no standardised laboratory test that accurately describes the status of magnesium. Due to the way in which magnesium is compartmentalised, typical blood and urine analytics may not provide an accurate proxy of magnesium status.	Workinger, Jayme L., Robert P. Doyle, and Jonathan Bortz. (2018) Challenges in the diagnosis of magnesium status. *Nutrients* 10, no. 9: 1202.
Nutritional status (Organic acids)	Insufficient evidence to prove clinical utility as valid indicator of general health (Guidelines issued a recognised professional organisation, Australia.	Nutritional status is not an indicator of an organic acid test. The normal range of organic acid levels in healthy asymptomatic individuals is varied. Further studies with well-defined patient groups exhibiting specific symptoms or diseases are warranted in order to discern between normal and pathological values.	RCPA Manual (2022) Organic acids https://www.rcpa.edu.au/Manuals/RCPA-Manual/Pathology-Tests/O/Organic-acids-urine. Accessed 31 May 2022.
Nutritional status (Selenium)	Insufficient evidence to prove clinical utility as valid indicator of general health (Guideline from a recognised professional organisation, Australia; consumer information from a recognised professional organisation, UK)	A selenium test is used for the detection of selenium deficiency in patients with dietary deficiency, especially patients on a non-supplemented synthetic amino acid diet, and the detection of selenium toxicity. Clinically significant selenium deficiency is manifest primarily as cardiomyopathy. This correlates poorly with plasma selenium levels and rarely occurs in selenium deficient patients on total parenteral nutrition. A nutrient test for selenium is useful but only in rare circumstances because clinically relevant deficiency is unlikely	RACP Manual (2022) Selenium https://www.rcpa.edu.au/Manuals/RCPA-Manual/Pathology-Tests/S/Selenium Accessed 11 October 2022. LabTests Online UK (2022) https://labtestsonline.org.uk/tests/unvalidated-or-misleading-laboratory-tests. Accessed 31 May 2022.
Nutritional status (Zinc)	Insufficient evidence to support clinical utility as valid indicator of general health (Guideline from professional and government organisation, Australia, New Zealand)	In a clinical setting, zinc plasma test is used to indicate zinc toxicity, or to assess people at risk of zinc deficiency (e.g. malnutrition, gastrointestinal disease). However, the zinc blood test is not very reliable for people with mild or low-level deficiency, because zinc is only present in small amounts in the body's cells.	RACP Manual (2022) Zinc https://www.rcpa.edu.au/Manuals/RCPA-Manual/Pathology-Tests/Z/Zinc. Accessed 11 October 2022. Auckland District Health Board (2022) Test Guide https://testguide.adhb.govt.nz/EGuide/. Accessed 11 October 2022.
Secretor status (Blood typing) (Antigens)	Insufficient evidence to prove clinical utility as valid indicator of general health (Peer-reviewed literature, CEBM level 1 - Systematic review)	Secretor status describes whether a person's blood type antigens (ABO blood type) are secreted in other body fluid such as saliva and mucus. Secretor status is determined by a FUT2 gene test. Secretor status test has been used to promote the 'Blood-Type Diet', based on the claim that each ABO blood type processes food differently, and adherence to a diet specific to an individual's ABO blood group could improve health, wellbeing, and energy levels and reduce risk of developing diseases such as cancer	Wang, J., García-Bailo, B., Nielsen, D. E., & El-Sohemy, A. (2014). ABO genotype,'blood-type'diet and cardiometabolic risk factors. *PloS one*, *9*(1), e84749.

For peer review only

		and cardiovascular disease. However, no evidence currently exists to validate the purported health benefits of blood type diets.	

6. Category 4, Subgroup 4A: Non-evidence-based tests with no potential clinical utility (Testing method is not recognised by the general medical community)

Description

The test method or condition tested for is not recognised by the general medical community. Current evidence does not support the validity of the test or the condition tested for. The results of non-evidence-based tests does not provide reliable clinical information to guide effective and beneficial management.

Evidence to support inclusion (4A):

There is insufficient evidence to support the clinical validity of the testing method for the condition it aims to test for.

Search & inclusion strategy

1. Google Advanced search terms: (*"test method and/or analyte" - see column 1)* AND diagnos* OR test* OR evidence*
2. PubMed and Google Advanced: (*"test method and/or analyte"- see column 1)* AND diagnos* OR test* OR evidence*

The first 30 records in each database were screened for relevance. i) We first prefaced EBM guidelines issued by Australian, Australasian and international professional or governmental organisations. The country or region of origin of the recommendations/guidelines is reported in Column 2. ii) If none were included, we included the peer-reviewed publication with the highest level of evidence (using CEBM Grading) were included. The type of evidence and CEBM grade level used is reported in Column 2. iii) If none were included, we then prefaced web-based consumer information issued by recognised expert health information organisations).

**Medical condition or test purpose; analyte and/or testing method**	**Reason for inclusion & Type of supporting evidence**	**Recommendation or summary of evidence**	**Reference**
Environmental toxins (Hair metal & mineral analysis)	Insufficient evidence to support the validity of the test method in the general medical community (Official information from government organisation, US)	A number of scientific issues need to be resolved before hair analysis can become a useful tool to understand environmental exposures. Although hair analysis may answer some questions about environmental exposure to certain substances, hair analysis often raises more questions than they answer. With very few exceptions, hair analysis results provide no insights as to whether an individual will develop health effects.	Agency for Toxic Substances and Disease Registry (2022) Analysis of Hair Samples: How Do Hair Sampling Results Relate to Environmental Exposures? https://www.atsdr.cdc.gov/HAC/hair_analysis/03-0330HairSampleTesting-Scientific.pdf. Accessed 10 October 2022.
Environmental toxins (Mycotoxin test)	Insufficient evidence to support the validity of the test method in the general medical community (Guideline issued by recognised intergovernmental organisation,	Mycotoxins are metabolites of some fungi that can cause illness primarily after ingestion of contaminated foods. Low levels of mycotoxins are found in many foods; therefore, mycotoxins are found in the urine of healthy persons. Mycotoxin levels that predict disease have not been established. Although	World Health Organizataion (2009) WHO guidelines for indoor air quality: Dampness and mould. http://www.who.int/indoorair/publications/7989289041683/en/. Accessed 10 October 2022.

For peer review only - http://bmjopen.bmj.com/site/about/guidelines.xhtml

	international)	mycotoxins can induce a wide range of adverse health effects in both animals and human beings, the evidence that they play a role in health problems related to indoor air is extremely weak	
Food allergy (Hair analysis, ALCAT, IgA, IgG)	Insufficient evidence to support the validity of the test method in the general medical community (Guideline issued by recognised professional organisation, Australasian)	Hair analysis, ALCAT, Immunoglobulin G (IgG) to foods are unproven, non evidence-based allergy 'tests' and 'treatments' that are provided by some unorthodox/alternative practitioners. There is currently no stringent government regulation of these methods. Consumers are strongly advised against the use of these tests.	Australasian Society of Clinical Immunology and Allergy (2021) Evidence-based vs Non-evidence based allergy tests and treatments https://www.allergy.org.au/images/pcc/ASCIA_PCC_Evidence-Based_vs_Non_Evidence-Based_Allergy_Tests-Treatments_FAQ_2021.pdf. Accessed 17 May 2022.
Hair loss (hormone tests)	Insufficient evidence to support the validity of the test method in the general medical community (Guideline issued by recognised professional organisation, Australia)	Hormone potentially contributes to hair health, but testing these analytes is not part of the diagnostic pathway for hair loss. Most cases of hair loss do not require pathology tests. Alopecia may be the result of a number of systemic or dermatological condition. Skin biopsy (from the active margin) may be helpful for lesions associated with inflammation or scarring.	RCPA Manual (2022) Alopecia https://www.rcpa.edu.au/Manuals/RCPA-Manual/Clinical-Problems/A/Alopecia. Accessed 17 May 2022.
Heavy metal chelation test (urine analysis)	Insufficient evidence to support the validity of the test method in the general medical community (Guideline issued by recognised professional organisation, US)	Post-chelator challenge urinary metal testing has not been scientifically validated, has no demonstrated benefit, and may be harmful when applied in the assessment and treatment of patients in whom there is concern for metal poisoning. The post-challenge urinary metal test and its application to assess of metal poisoning often leads to unwarranted and prolonged oral and/or intravenous administration of chelating agents. Chelation therapy based on such laboratory values, in addition to being of no benefit to patient outcome, may actually prove harmful.	American College of Medical Toxicology (2010). American College of Medical Toxicology position statement on post-chelator challenge urinary metal testing. *Journal of Medical Toxicology*, *6*(1), 74-75.
Histamine intolerance (diamine oxidase level)	Insufficient evidence to support the validity of the test method in the general medical community (Peer-reviewed literature, CEBM Grade Level 2 – level 2 evidence synthesis)	Histamine intolerance is a disorder associated with an impaired ability to metabolise ingested histamine, causing sensitive response to histamine in food. However, the symptoms are nonspecific an there is currently no consensus or validation of diagnostic tests for this condition. Currently available studies indicate an etiological relationship between diamine oxidase (DAO) deficiency and certain symptoms or disorders related to histamine intolerance. However, the evidence for the validity of blood DAO activity measurements for the diagnosis of histamine intolerance is neither	Comas-Basté, O., Sánchez-Pérez, S., Veciana-Nogués, M. T., Latorre-Moratalla, M., & Vidal-Carou, M. D. C. (2020). Histamine intolerance: The current state of the art. *Biomolecules*, *10*(8), 1181.

For peer review only - http://bmjopen.bmj.com/site/about/guidelines.xhtml

		abundant nor conclusive.	
Liver detoxification test (urine metabolites)	Insufficient evidence to support the validity of the test method among the general medical community (Guideline issued by recognised professional organisation, UK)	Liver detoxification tests, used mostly in complementary and alternative medicine (CAM), involves taking several common drugs e.g. paracetamol, aspirin and caffeine and measuring their metabolites in blood and urine afterwards. The amount and ratios of metabolites present are used as an indication of the amount of metabolism taking place in the liver. There is no evidence that the results of liver detoxification tests have any clinical utility in patients who do not have advanced liver disease. Genuine liver damage can be diagnosed using clinical examinations, imaging and other laboratory tests.	Lab Tests Online UK (2022) Unvalidated or misleading laboratory tests, https://labtestsonline.org.uk/tests/unvalidated-or-misleading-laboratory-tests. Accessed 21 May 2022
Mental health nutrition ('Pfeiffer Test')	Insufficient evidence to support the validity of the test method among the general medical community (Peer-reviewed literature, CEBM Grade Level 3 – level 3 evidence synthesis)	The Pfeiffer Test (to be distinguished from Pfeiffer disease) is a test used in CAM to analyse nutritional elements including zinc, copper and zinc-copper ratio in blood, promoted as an indicator of mental disorders such as attention deficit disorder (ADD), attention deficit hyperactivity disorder (ADHD), schizophrenia, Autism, learning disorders and mental disorders. While there is evidence to suggest that people with mental illness are likely to have lower or deficient levels of zinc or a copper-zinc imbalance, the causal link between low zinc and zinc-copper ratio imbalance to mental disorders is not established.	Robberecht, H., Verlaet, A. A., Breynaert, A., De Bruyne, T., & Hermans, N. (2020). Magnesium, iron, zinc, copper and selenium status in attention-deficit/hyperactivity disorder (ADHD). Molecules, 25(19), 4440.
Nagalase test	Insufficient evidence to support the validity of the test method among the general medical community (Guideline issued by recognised professional organisation, international & Australia)	Nagalase is an enzyme produced by cancerous cells. It is an indicator of early or potential cancer. A test for the Nagalase enzyme is most notably used as an indicator of a patients' response to a controversial cancer treatment using Gc Macrophage Activating Factor (GcMAF). There is no credible evidence to show that Gc-MAF can rebuild the immune system or eradicate early stage cancer. Studies that claim successful treatment using GcMAF have since been retracted. The Anticancer Fund and Cancer Council Australia have issued public warnings against using GcMAF as a cancer treatment	Cancer Council Australia (2022) Can GcMAF rebuild the immune system to eradicate early stage cancer? https://www.cancer.org.au/iheard/can-gcmaf-rebuild-the-immune-system-to-eradicate-early-stage-cancer. Accessed 17 May 2022<br>Ugarte, A., Bouche, G., & Meheus, L. [Anticancer Fund] (2014). Inconsistencies and questionable reliability of the publication "immunotherapy of metastatic colorectal cancer with vitamin D-binding protein-derived macrophages-activating, GcMAF" by Yamamoto et al. Cancer Immunology Immunotherapy, 63(12), 1347-8. doi: 10.1007/s00262-014-1587-y

For peer review only - http://bmjopen.bmj.com/site/about/guidelines.xhtml

For peer review only

Neurotransmitters urine test (Neurotransmitters in urine)	Insufficient evidence to support the validity of the test method among the general medical community (Peer-reviewed literature, CEBM Grade Level 2 – level 2 evidence synthesis)	Neurotransmitters excreted in the urine may have a place in clinical practice as a biomarker of nervous system function assess disturbances and monitor treatment efficacy for depression, ADHD, and inflammation. However, Urinary neurotransmitter assessment is yet to be accepted as diagnostic for depression or any particular disease or condition. There is variability of baseline measurements. The fraction of monoamines from the nervous system that appear in the urine, and under what conditions, is unclear.	Marc, D. T., Ailts, J. W., Campeau, D. C. A., Bull, M. J., & Olson, K. L. (2011). Neurotransmitters excreted in the urine as biomarkers of nervous system activity: validity and clinical applicability. *Neuroscience & Biobehavioral Reviews*, *35*(3), 635-644.
Small intestinal bacterial overgrowth (SIBO) - Hydrogen & methane breath test	Insufficient evidence to support the validity of the test method among the general medical community (Peer-reviewed literature, CEBM Grade Level 1 - Systematic review)	The lactulose hydrogen breath test is not a standard diagnostic test for SIBO. There is no validated breath test for the diagnosis of SIBO.	Khoshini, R., Dai, S. C., Lezcano, S., & Pimentel, M. (2008). A systematic review of diagnostic tests for small intestinal bacterial overgrowth. *Digestive diseases and sciences*, *53*(6), 1443-1454.

7. Category 4, Subgroup 4B: Non-evidence-based tests with no potential clinical utility (condition tested for is not recognised by the general medical community)

Evidence to support inclusion

There is insufficient evidence to support the condition as established and recognised by the general medical community

Search & inclusion strategy

1. Google Advanced search terms: (*condition - see column 1)* AND diagnos* OR test* OR evidence*

The first 30 records were screened for relevance.

2. PubMed and Google Advanced: (*condition - see column 1)* AND diagnos* OR test* OR evidence*

The first 30 records in each database were screened for relevance. i) We first prefaced EBM guidelines issued by Australian, Australasian and international professional or governmental organisations. The country or region of origin of the recommendations/guidelines is reported in Column 2. ii) If none were included, we included the peer-reviewed publication with the highest level of evidence (using CEBM Grading) were included. The type of evidence and CEBM grade level used is reported in Column 2. iii) If none were included, we then prefaced web-based consumer information issued by recognised expert health information organisations).

**Medical condition or test purpose; analyte and/or testing method**	**Reason for inclusion & Type of supporting evidence**	**Recommendation or summary of evidence**	**Reference**
Adrenal fatigue (DHEA & Cortisol)	Insufficient evidence to support the condition as established and recognised among the general medical community (Peer-reviewed literature, CEBM	The term ‘adrenal fatigue’ has been used by some doctors, healthcare providers, and the general media to describe an alleged condition caused by chronic exposure to stressful situations. Despite this, adrenal fatigue has not been recognised by any Endocrinology	Cadegiani, F. A., & Kater, C. E. (2016). Adrenal fatigue does not exist: a systematic review. *BMC endocrine disorders*, *16*(1), 1-16.

For peer review only - http://bmjopen.bmj.com/site/about/guidelines.xhtml

	Grade Level 1 - Systematic review)	society, as there is no sufficient evidence for its existence (Cadagiani & Kater, 2016). A systematic review by Cadagiani & Kater (2016) of 58 validated studies show that there is no substantiation that adrenal fatigue is an actual medical condition.	
Intestinal permeability (Lactulose & mannitol; stool analysis)	Insufficient evidence to support the condition as established and recognised among the general medical community (Peer-reviewed literature, CEBM Grade Level 3 – level 3 evidence synthesis)	Intestinal permeability (Leaky gut syndrome) describes a condition in which bacteria and toxins are able to pass through the intestinal wall into the bloodstream. Although barrier dysfunction has been described in a number of disorders, its role in their pathogenesis remains to be defined.	Quigley, E. M. (2016). Leaky gut–concept or clinical entity? *Current opinion in gastroenterology*, *32*(2), 74-79.
Paediatric autoimmune neuropsychiatric disorders (PANDAS) syndrome (Antibody test)	Insufficient evidence to support the condition as established and recognised among the general medical community (Peer-reviewed literature, CEBM Grade Level 2 – level 2 evidence synthesis)	Pediatric autoimmune neuropsychiatric disorders associated with streptococcal infections (PANDAS) is a hypothetical for a subset of children with rapid onset of obsessive-compulsive disorder (OCD) or tic disorders. Although there is a body of evidence that supports the existence of PANDAS and related conditions, it remains a controversial diagnosis. A number of studies investigated the PANDAS hypothesis do support the existence of these conditions. Further studies failed to identify significant differences in specific serum autoantibodies between PANDAS patients and healthy controls. Currently, none of the studied diagnostic approaches of PANDAS is sufficient to confirm the diagnosis.	Wilbur, C., Bitnun, A., Kronenberg, S., Laxer, R. M., Levy, D. M., Logan, W. J., ... & Yeh, E. A. (2019). PANDAS/PANS in childhood: Controversies and evidence. *Paediatrics & child health*, *24*(2), 85-91.
Pyrrole disorder (HPL & neurotoxin)	Insufficient evidence to support the condition as established and recognised among the general medical community (Peer-reviewed literature, CEBM Grade Level 1 - Systematic review)	Pyrrole disorder refers to a mood disorder attributed to elevated levels of pyrroles in the urine, also known as hydroxyhemepyrrolin-2-one (HPL). Hypothetically, the raised pyrrole levels lead to depletions in zinc and vitamin B_6, which, in turn, result in a range of psychiatric disorders, such as schizophrenia, anxiety, and depression.<br>However, elevated HPL is a clinically observed, but poorly researched biomarker with unclear associations with mental disorders.	Warren B, Sarris J, Mulder RT, Rucklidge JJ. Pyroluria: Fact or Fiction? J Altern Complement Med. 2021 May;27(5):407-415. doi: 10.1089/acm.2020.0151. Epub 2021 Apr 27. PMID: 33902305.

[i] Oxford Centre for Evidence-Based Medicine (CEBM): Levels of Evidence (2009) https://www.cebm.ox.ac.uk/resources/levels-of-evidence/oxford-centre-for-evidence-based-medicine-levels-of-evidence-march-2009 . Accessed 2 May 2022

For peer review only - http://bmjopen.bmj.com/site/about/guidelines.xhtml
